# Supplementary material for: Application of various mixtures of medicinal herbs in the diet of laying hens: Evaluating preventive approach of fatty liver syndrome
Source: PLoS One. 2025 Oct 24;20(10):e0330363. doi: 10.1371/journal.pone.0330363 (PMC12551855; doi:10.1371/journal.pone.0330363)
Supplement: S1 File — (DOCX) [file pone.0330363.s001.docx]

**Table 1**. Shallot (*Allium cepa*) oil composition determined by GC-MS

| **Component** | RT | % |
| --- | --- | --- |
| Carbonic acid, nonyl prop-1-en-2-yl ester | 6.6108 | 0.041 |
| Dimethyl trisulfide | 7.9546 | 12.65 |
| Disulfide, methyl (methylthio)methyl | 10.7297 | 22.553 |
| Ethane, 2-bromo-1,1-difluoro- | 12.2974 | 0.039 |
| 2,4,5,7-Tetrathiaoctane 2-oxide | 14.1913 | 7.002 |
| Caryophyllene | 14.9703 | 0.373 |
| (1R,4R,4aS,8aR)-4,7-Dimethyl-1-(prop-1-en-2-yl)-1,2,3,4,4a,5,6,8a-octahydronaphthalene | 15.462 | 0.058 |
| Benzene, 1-(1,5-dimethyl-4-hexenyl)-4-methyl- | 15.7054 | 0.468 |
| 1,3-Cyclohexadiene, 5-(1,5-dimethyl-4-hexenyl)-2-methyl-, [S-(R*,S*)]- | 15.8515 | 0.284 |
| 6.alpha-Cadina-4,9-diene, (-)- | 15.9683 | 0.165 |
| β -Bisabolene | 16.017 | 0.177 |
| 2 6-bis (11-dimethylethyl)-4-methyl-phenol") | 16.0657 | 0.042 |
| 2,4,5,7-Tetrathiaoctane | 16.1874 | 8.439 |
| Ar-tumerone | 17.984 | 0.044 |
| 2,3,5,7-Tetrathiaoctane 3,3-dioxide | 19.2547 | 0.168 |
| Hexadecanoic acid, ethyl ester | 21.2605 | 0.109 |
| 2-Methyltetracosane | 22.2099 | 0.065 |
| Linoleic acid ethyl ester | 22.8477 | 0.364 |
| Bis(2-ethylhexyl) phthalate | 25.652 | 0.12 |
| 1,2-Benzenedicarboxylic acid, bis(2-ethylhexyl) ester | 26.0464 | 46.839 |

RT= retention time

**Table 2**. Green tea (*Camellia sinensis*) oil composition determined by GC-MS

| Compound | RT | % |
| --- | --- | --- |
| α- Pinene | 7.25 | 0.074 |
| 2(10)-Pinene | 8.07 | 0.212 |
| 3-Carene | 8.69 | 0.098 |
| Eucalyptol | 9.07 | 0.068 |
| (2E)-Dodec-2-en-1-yl methyl ether | 10.42 | 0.057 |
| trans,trans-1,6-Dimethylspiro[4.5]decane | 12.29 | 0.047 |
| α- Terpinene | 13.82 | 0.059 |
| Copaene | 14.37 | 0.12 |
| Caryophyllene | 14.97 | 0.471 |
| (Z,Z)-alpha-Farnesene | 15.11 | 0.101 |
| (1R,4R,4aS,8aR)-4,7-Dimethyl-1-(prop-1-en-2-yl)-1,2,3,4,4a,5,6,8a-octahydronaphthalene | 15.46 | 0.187 |
| Benzene, 1-(1,5-dimethyl-4-hexenyl)-4-methyl- | 15.72 | 0.481 |
| 1,3-Cyclohexadiene, 5-(1,5-dimethyl-4-hexenyl)-2-methyl-, [S-(R*,S*)]- | 15.86 | 0.128 |
| Naphthalene, 1,2,3,4,4a,5,6,8a-octahydro-7-methyl-4-methylene-1-(1-methylethyl)-, (1α,4aβ,8aα)- | 15.96 | 0.146 |
| β- Bisabolene | 16.02 | 0.263 |
| cis-Verbenol | 17.02 | 0.047 |
| Cryptomeridiol | 17.83 | 0.097 |
| Carbonic acid, decyl prop-1-en-2-yl ester | 19.78 | 0.15 |
| 3-Aminomethyl-3,5,5-trimethylcyclohexanol, cis- | 22.44 | 0.067 |
| Bis(2-ethylhexyl) phthalate | 25.65 | 0.215 |
| Carvacrol, TBDMS derivative | 25.86 | 0.054 |
| Bis(2-ethylhexyl) phthalate | 26.03 | 96.298 |

RT= retention time

**Table 3**. Sumac (*Rhus coriaria*) oil composition determined by GC-MS

| **Component** | RT | % |
| --- | --- | --- |
| 2-Pinene | 7.25 | 0.257 |
| 2(10)-Pinene | 8.07 | 1.003 |
| 3-Carene | 8.69 | 0.782 |
| Tricyclo[3.2.1.0(1,5)]octane | 9.07 | 0.319 |
| 2-Cyclohexen-1-one, 2-methyl-5-(1-methylethenyl)- | 12.65 | 0.336 |
| Cyclohexene, 4-ethenyl-4-methyl-3-(1-methylethenyl)-1-(1-methylethyl)-, (3R-trans)- | 13.82 | 0.618 |
| Copaene | 14.36 | 1.508 |
| Cyclohexane, 1-ethenyl-1-methyl-2,4-bis(1-methylethenyl)-, [1S-(1α,2β,4β)]- | 14.56 | 0.604 |
| Caryophyllene | 14.97 | 7.785 |
| trans-alpha-Bergamotene | 15.11 | 2.364 |
| (3E,6E)-3,7,11-trimethyl-1,3,6,10-dodecatetraene | 15.34 | 0.198 |
| 1,4,7,-Cycloundecatriene, 1,5,9,9-tetramethyl-, Z,Z,Z- | 15.40 | 0.558 |
| (4S,6S)-6-isopropenyl-4-methyl-4,4a,5,6,7,8-hexahydro-2(3H)-naphthalenone | 15.46 | 0.249 |
| 1-(1,5-dimethyl-4-hexenyl)-4-methyl- Benzene | 15.71 | 3.219 |
| 1,3-Cyclohexadiene, 5-(1,5-dimethyl-4-hexenyl)-2-methyl-, [S-(R*,S*)]- | 15.85 | 1.915 |
| α -Himachalene | 15.93 | 1.054 |
| β- Bisabolene | 16.02 | 2.875 |
| Cyclohexene, 3-(1,5-dimethyl-4-hexenyl)-6-methylene-, [S-(R*,S*)]- | 16.22 | 2.154 |
| 7-Benzofuranol, 2,3-dihydro-2,2-dimethyl- | 16.64 | 0.243 |
| 3-Cyclohexen-1-carboxaldehyde, 3,4-dimethyl- | 17.02 | 0.415 |
| ar-Turmerone | 18.00 | 0.287 |
| ar-Turmerone | 18.05 | 0.263 |
| 1,3,6,10-Cyclotetradecatetraene, 3,7,11-trimethyl-14-(1-methylethyl)-, [S-(E,Z,E,E)]- | 20.77 | 0.445 |
| Bis(2-ethylhexyl) phthalate | 26.01 | 70.55 |

RT= retention time

**Table 4**. Fumitory (*Fumaria officinalis*) oil composition determined by GC-MS

| **Component** | RT | % |
| --- | --- | --- |
| Nonane | 6.58 | 0.058 |
| 2-Pinene | 7.25 | 0.103 |
| 2(10)-Pinene | 8.07 | 0.433 |
| 3-Carene | 8.68 | 0.333 |
| Cyclohexene, 1-methyl-4-(1-methylethenyl)-, (S)- | 9.02 | 0.088 |
| 2-Oxabicyclo[2.2.2]octane, 1,3,3-trimethyl- | 9.07 | 0.067 |
| Hexanal, 5-methyl- | 10.24 | 0.063 |
| Carbonic acid, nonyl prop-1-en-2-yl ester | 11.77 | 0.107 |
| Benzaldehyde, 4-(1-methylethyl)- | 12.52 | 0.501 |
| Bornyl acetate | 13.09 | 0.053 |
| Phenol, 5-methyl-2-(1-methylethyl)- | 13.17 | 1.764 |
| Carvacrol | 13.31 | 5.588 |
| delta-Elemene | 13.82 | 0.539 |
| 1H-Cyclopenta[1,3]cyclopropa[1,2]benzene, octahydro-7-methyl-3-methylene-4-(1-methylethyl)-, [3aS-(3aα,3bβ,4β,7α,7aS*)]- | 13.98 | 0.118 |
| 1,2,4-Metheno-1H-indene, octahydro-1,7a-dimethyl-5-(1-methylethyl)-, [1S-(1α,2α,3aβ,4α,5α,7aβ,8S*)]- | 14.25 | 0.089 |
| Copaene | 14.36 | 0.977 |
| 2-Buten-1-one, 1-(2,6,6-trimethyl-1,3-cyclohexadien-1-yl)-, (E)- | 14.44 | 0.154 |
| 2,4-Diisopropenyl-1-methyl-1-vinylcyclohexane | 14.56 | 0.347 |
| 6,8-Nonadien-2-one, 6-methyl-5-(1-methylethylidene)- | 14.84 | 0.063 |
| Caryophyllene | 14.97 | 6.802 |
| cis-alpha-Bergamotene | 15.11 | 0.109 |
| 1H-Cycloprop[e]azulene, decahydro-1,1,7-trimethyl-4-methylene-, (1aR,4aR,7R,7aR,7bS)-(+)- | 15.21 | 0.061 |
| Unknown from lime oil | 15.34 | 0.174 |
| Humulene | 15.40 | 0.292 |
| (1R,4R,4aS,8aR)-4,7-Dimethyl-1-(prop-1-en-2-yl)-1,2,3,4,4a,5,6,8a-octahydronaphthalene | 15.46 | 0.637 |
| Benzene, 1-(1,5-dimethyl-4-hexenyl)-4-methyl- | 15.70 | 3.822 |
| 1,3-Cyclohexadiene, 5-(1,5-dimethyl-4-hexenyl)-2-methyl-, [S-(R*,S*)]- | 15.85 | 3.075 |
| γ-Muurolene | 15.96 | 1.367 |
| β -Bisabolene | 16.01 | 1.527 |
| Naphthalene, 1,2,3,4,4a,5,6,8a-octahydro-7-methyl-4-methylene-1-(1-methylethyl)-, (1α,4aβ,8aα)- | 16.14 | 0.052 |
| β -Sesquiphellandrene | 16.21 | 2.188 |
| 1,1,7-Trimethyl-4-methylenedecahydro-1H-cyclopropa[e]azulen | 16.42 | 0.062 |
| 1-Isopropyl-4,7-Dimethyl-1,2-Dihydronaphthalene | 16.50 | 0.104 |

RT= retention time

**Table 4**. Fumitory (Fumaria officinalis) oil composition determined by GC-MS

| **Component** | RT | % |
| --- | --- | --- |
| Caryophyllenyl alcohol | 16.87 | 0.049 |
| Hexadecane | 17.02 | 0.506 |
| Alloaromadendrene | 17.51 | 0.167 |
| (E)-1-(6,10-Dimethylundec-5-en-2-yl)-4-methylbenzene | 17.82 | 0.068 |
| Ar-tumerone | 17.90 | 0.384 |
| Heptadecane | 18.15 | 0.09 |
| Curlone | 18.31 | 0.043 |
| 2-Propenoic acid, 2-(acetylamino)- | 18.46 | 0.047 |
| 1-[[Bis(methylthio)methylene]acetyl]-2-(4-(4-methoxyphenyl)-1,3-butadienyl)cyclopropane | 19.10 | 0.053 |
| Octadecane | 19.23 | 0.125 |
| Phosphorothioic acid, O,O-diethyl 2-isopropyl-6-methyl-4-pyrimidinyl ester | 19.39 | 0.092 |
| Isopropyl myristate | 19.51 | 0.128 |
| 2-Pentadecanone, 6,10,14-trimethyl- | 19.73 | 1.461 |
| Phthalic acid, 7-bromoheptyl isobutyl ester | 20.02 | 0.869 |
| Nonadecane | 20.26 | 0.047 |
| Hexadecanoic acid, methyl ester | 20.53 | 1.231 |
| Phytol | 20.76 | 0.129 |
| 1,2-Benzenedicarboxylic acid, butyl 2-methylpropyl ester | 20.97 | 0.215 |
| n-Hexadecanoic acid | 21.20 | 8.101 |
| 9,12-Octadecadienoic acid (Z,Z)-, methyl ester | 22.17 | 2.509 |
| 9-Octadecenoic acid, methyl ester, (E)- | 22.22 | 2.192 |
| 3-Eicosene, (E)- | 22.34 | 0.752 |
| Hexadecanoic acid | 22.44 | 0.112 |
| n-Hexadecanoic acid | 22.62 | 0.095 |
| 9,12-Octadecadienoic acid (Z,Z)- | 22.78 | 7.68 |
| 9-Octadecenoic acid (Z)- | 23.94 | 0.22 |
| Hexanedioic acid, bis(2-ethylhexyl) ester | 24.79 | 1.102 |
| Pentacosane | 25.56 | 0.227 |
| 9,12-Octadecadienoic acid (Z,Z)- | 25.64 | 0.115 |
| Bis(2-ethylhexyl) phthalate | 26.03 | 38.17 |
| Eicosane | 27.07 | 0.3 |
| Squalene | 28.04 | 0.565 |
| Octadecane | 28.48 | 0.249 |
| 4-Hydroxyphenyl pyrrolidinyl thione | 29.94 | 0.191 |

RT= retention time

**Table 5.** Milk thistle (*Silybum marianum*) oil composition determined by GC-MS

| **Component** | RT | % |
| --- | --- | --- |
| (1R)-2,6,6-Trimethylbicyclo[3.1.1]hept-2-ene | 7.25 | 0.329 |
| β- Pinene | 8.08 | 1.642 |
| 3- Carene | 8.69 | 1.864 |
| D-Limonene | 9.04 | 0.387 |
| Cyclohexene, 4-ethenyl-4-methyl-3-(1-methylethenyl)-1-(1-methylethyl)-, (3R-trans)- | 13.82 | 2.103 |
| 1H-Cyclopenta[1,3]cyclopropa[1,2]benzene, octahydro-7-methyl-3-methylene-4-(1-methylethyl)-, [3aS-(3aα,3bβ,4β,7α,7aS*)]- | 13.99 | 0.289 |
| α- ylangene | 14.26 | 0.122 |
| α- Copaene | 14.37 | 4.129 |
| β- Elemene | 14.57 | 0.995 |
| α- Gurjunene | 14.83 | 0.058 |
| Caryophyllene | 14.98 | 34.68 |
| trans-alpha-Bergamotene | 15.12 | 0.402 |
| 1,7-Octadien-3-ol, 3,7-dimethyl- | 15.22 | 0.051 |
| Aromandendrene | 15.34 | 0.098 |
| β -Selinene | 15.41 | 1.473 |
| (1R,4R,4aS,8aR)-4,7-Dimethyl-1-(prop-1-en-2-yl)-1,2,3,4,4a,5,6,8a-octahydronaphthalene | 15.46 | 0.308 |
| Bicyclo[4.1.0]heptan-3-ol, 4,7,7-trimethyl-, (1α,3β,4α,6α)- | 15.57 | 0.062 |
| Benzene, 1-(1,5-dimethyl-4-hexenyl)-4-methyl- | 15.71 | 1.617 |
| 1,3-Cyclohexadiene, 5-(1,5-dimethyl-4-hexenyl)-2-methyl-, [S-(R*,S*)]- | 15.86 | 1.421 |
| α- Gurjunene | 15.93 | 0.868 |
| β- Bisabolene | 16.03 | 0.966 |
| γ- Muurolene | 16.14 | 0.065 |
| Tricyclo[4.4.0.02,7]dec-3-ene, 1,3-dimethyl-8-(1-methylethyl)-, stereoisomer | 16.23 | 1.192 |
| 1,4-Methanobenzocyclodecene, 1,2,3,4,4a,5,8,9,12,12a-decahydro- | 16.93 | 0.074 |
| (-)-5-Oxatricyclo[8.2.0.0(4,6)]dodecane,12-trimethyl-9-methylene-, [1R-(1R*,4R*,6R*,10S*)]- | 17.03 | 0.836 |
| 9-(2',2'-Dimethylpropanoilhydrazono)-3,6-dichloro-2,7-bis-[2-(diethylamino)-ethoxy]fluorene | 25.66 | 0.089 |
| Bis(2-ethylhexyl) phthalate | 26.02 | 40.675 |
| 7a,9c-(Iminoethano)phenanthro[4,5-bcd]furan, 4aalpha,5-dihydro-3-methoxy-12-methyl- | 27.99 | 0.519 |
| 1,4-benzenediamine, 2,3,5,6-tetramethyl-N1,N4-diphenyl- | 28.49 | 2.687 |

RT= retention time

**Table 6**. Chicory (*Cichorium intybus L.*) oil composition determined by GC-MS

| **Component** | RT | % |
| --- | --- | --- |
| Octane, 4-methyl- | 5.84 | 0.025 |
| Nonane | 6.58 | 0.032 |
| 2-Pinene | 7.25 | 0.296 |
| 2,2-dimethyl-3-methylene-bicyclo [2.2.1]heptane | 7.53 | 0.028 |
| Bicyclo[3.1.1]heptane, 6,6-dimethyl-2-methylene-, (1S)- | 8.07 | 1.078 |
| Furan, 2-pentyl- | 8.36 | 0.249 |
| Decane | 8.49 | 0.029 |
| delta- 3-Carene | 8.68 | 1.137 |
| Benzene, 1-methyl-2-(1-methylethyl)- | 8.95 | 0.097 |
| Cyclohexene, 1-methyl-4-(1-methylethenyl)- | 9.02 | 0.49 |
| γ -Terpinene | 9.55 | 0.034 |
| Cyclohexene, 3-methyl-6-(1-methylethylidene)- | 10.05 | 0.054 |
| N-[3-[N-Aziridyl]propylidene]tetrahydrofurfurylamine | 10.23 | 0.036 |
| Nonanoic acid, isohexyl ester | 10.49 | 0.044 |
| 1-Cyclohexyl-2-methyl-prop-2-en-1-one | 10.99 | 0.029 |
| Bicyclo[2.2.1]heptan-2-ol, 1,7,7-trimethyl-, (1S-endo)- | 11.33 | 0.028 |
| 3-Cyclohexen-1-ol, 4-methyl-1-(1-methylethyl)- | 11.52 | 0.035 |
| Benzenemethanol, α,α,4-trimethyl- | 11.64 | 0.07 |
| 2-Carene | 11.71 | 0.074 |
| Dodecane | 11.76 | 0.107 |
| Fenchyl acetate | 12.13 | 1.287 |
| Fenchyl acetate | 12.33 | 0.053 |
| Benzene, 1-methoxy-4-methyl-2-(1-methylethyl)- | 12.46 | 0.213 |
| 6-Undecanone | 12.84 | 0.048 |
| Bornyl acetate | 13.09 | 0.955 |
| trans-Pinocarvyl acetate | 13.28 | 0.213 |
| 2,3-Diazabicyclo[2.2.1]hept-2-ene, 4-methyl-1-(pent-4-en-1-yl)- | 13.66 | 0.047 |
| 4(10)-Thujene | 13.71 | 0.14 |
| delta-Elemene | 13.82 | 0.14 |
| 2-Carene | 13.95 | 1.171 |
| Cycloheptane, 1-bromo-3-iodo- | 14.12 | 0.035 |
| 6-Dodecanone | 14.21 | 0.072 |
| 1,2,4-Metheno-1H-indene, octahydro-1,7a-dimethyl-5-(1-methylethyl)-, [1S-(1α,2α,3aβ,4α,5α,7aβ,8S*)]- | 14.26 | 0.578 |
| Copaene | 14.37 | 0.739 |
| 1H-Cycloprop[e]azulene, decahydro-1,1,7-trimethyl-4-methylene-, (1aR,4aS,7R,7aR,7bS)-(-)- | 14.47 | 0.262 |
| β -Elemene | 14.56 | 0.683 |

RT= retention time

**Table 6.** Chicory (*Cichorium intybus L.*) oil composition determined by GC-MS

| **Component** | RT | % |
| --- | --- | --- |
| 1,1,7-Trimethyl-4-methylenedecahydro-1H-cyclopropa[e]azulen | 14.68 | 0.185 |
| (+)-β -Funebrene | 14.91 | 0.932 |
| Caryophyllene | 14.97 | 2.019 |
| 1H-Cycloprop[e]azulene, decahydro-1,1,7-trimethyl-4-methylene-, (1aR,4aS,7R,7aR,7bS)-(-)- | 15.11 | 0.427 |
| 1,5,5-Trimethyl-6-methylene-cyclohexene | 15.18 | 0.029 |
| 1H-Cycloprop[e]azulene, decahydro-1,1,7-trimethyl-4-methylene-, (1aR,4aR,7R,7aR,7bS)-(+)- | 15.22 | 0.379 |
| (3aR,4R,8R,8aS)-3a,4,8a-Trimethyl-7-methylenedecahydro-4,8-methanoazulene-rel- | 15.31 | 2.068 |
| 1,4,7,-Cycloundecatriene, 1,5,9,9-tetramethyl-, Z,Z,Z- | 15.40 | 0.141 |
| 1-Methyl-4-(6-methylhept-5-en-2-yl)cyclohexa-1,3-diene | 15.46 | 0.231 |
| Cinnamaldehyde, alpha-pentyl- | 15.55 | 0.132 |
| Bicyclo[7.2.0]undec-4-ene, 4,11,11-trimethyl-8-methylene- | 15.58 | 0.072 |
| Benzene, 1-(1,5-dimethyl-4-hexenyl)-4-methyl- | 15.70 | 5.001 |
| 9-Isopropyl-1-methyl-2-methylene-5-oxatricyclo[5.4.0.0(3,8)]undecane | 15.78 | 0.08 |
| trans-alpha-Bergamotene | 15.85 | 2.793 |
| 1.xi.,6.xi.,7.xi.-Cadina-4,9-diene | 15.95 | 0.413 |
| 2H-3,9a-Methano-1-benzoxepin, octahydro-2,2,5a,9-tetramethyl-, [3R-(3α,5aα,9α,9aα)]- | 16.03 | 4.258 |
| Benzene, 1-methyl-4-(1,2,2-trimethylcyclopentyl)-, (R)- | 16.07 | 1.779 |
| Naphthalene, 1,2,3,4,4a,5,6,8a-octahydro-7-methyl-4-methylene-1-(1-methylethyl)-, (1α,4aβ,8aα)- | 16.14 | 1.305 |
| Cyclohexene, 3-(1,5-dimethyl-4-hexenyl)-6-methylene-, [S-(R*,S*)]- | 16.21 | 2.142 |
| 1H-Cycloprop[e]azulene, decahydro-1,1,7-trimethyl-4-methylene-, (1aR,4aR,7R,7aR,7bS)-(+)- | 16.42 | 0.339 |
| 4-Isopropyl-6-methyl-1-methylene-1,2,3,4-tetrahydronaphthalene | 16.50 | 0.134 |
| Cyclohexanemethanol, 4-ethenyl-α,α,4-trimethyl-3-(1-methylethenyl)-, [1R-(1α,3α,4β)]- | 16.56 | 0.94 |
| Bicyclo[7.2.0]undec-4-ene, 4,11,11-trimethyl-8-methylene-,[1R-(1R*,4Z,9S*)]- | 16.65 | 0.205 |
| Spiro[2.5]octane, 3,3-dimethyl-2-(1-buten-3-on-1-yl)- | 16.72 | 0.277 |
| 1H-Cycloprop[e]azulene, decahydro-1,1,7-trimethyl-4-methylene-, (1aR,4aS,7R,7aR,7bS)-(-)- | 16.85 | 0.116 |
| 3-Cyclohexene-1-methanol, 5-hydroxy-α,α,4-trimethyl, (1S-trans)- | 16.89 | 0.064 |
| 1H-Cycloprop[e]azulen-7-ol, decahydro-1,1,7-trimethyl-4-methylene-, [1ar-(1aα,4aα,7β,7aβ,7bα)]- | 16.95 | 0.52 |
| (-)-5-Oxatricyclo[8.2.0.0(4,6)]dodecane,,12-trimethyl-9-methylene-, [1R-(1R*,4R*,6R*,10S*)]- | 17.02 | 1.94 |
| Azulene, 1,2,3,3a,4,5,6,7-octahydro-1,4-dimethyl-7-(1-methylethenyl)-, [1R-(1α,3aβ,4α,7β)]- | 17.13 | 0.279 |

RT= retention time

**Table 6**. Chicory (*Cichorium intybus L.*) oil composition determined by GC-MS

| **Component** | RT | % |
| --- | --- | --- |
| (S,1Z,6Z)-8-Isopropyl-1-methyl-5-methylenecyclodeca-1,6-diene | 17.18 | 0.083 |
| 5-epi-7-epi-α-Eudesmol | 17.25 | 0.324 |
| (1R,3E,7E,11R)-1,5,5,8-Tetramethyl-12-oxabicyclo[9.1.0]dodeca-3,7-diene | 17.33 | 1.335 |
| 2-((2S,4aR)-4a,8-Dimethyl-1,2,3,4,4a,5,6,7-octahydronaphthalen-2-yl)propan-2-ol | 17.46 | 0.918 |
| β -Maaliene | 17.57 | 0.663 |
| delta-Cadinene | 17.66 | 0.124 |
| 10s,11s-Himachala-3(12),4-diene | 17.73 | 0.412 |
| β -Eudesmol | 17.81 | 5.118 |
| aR-Turmerone | 17.88 | 1.631 |
| Naphthalene, 1,6-dimethyl-4-(1-methylethyl)- | 18.05 | 0.05 |
| α -Bisabolol | 18.12 | 1.392 |
| 6,10-Dimethyl-3-(1-methylethylidene)-1-cyclodecene | 18.25 | 0.037 |
| 2-Methyl-6-(4-methylenecyclohex-2-en-1-yl)hept-2-en-4-one | 18.30 | 0.908 |
| Silane, ethenylmethylphenyl- | 18.39 | 0.107 |
| 4,6,6-Trimethyl-2-(3-methylbuta-1,3-dienyl)-3-oxatricyclo[5.1.0.0(2,4)]octane | 18.47 | 0.024 |
| Valerena-4,7(11)-diene | 18.51 | 0.154 |
| Alloaromadendrene | 18.60 | 0.063 |
| Alloaromadendrene | 18.75 | 0.08 |
| Vulgarol B | 18.86 | 0.051 |
| 3,7-Cyclodecadien-1-one, 3,7-dimethyl-10-(1-methylethylidene)-, (E,E)- | 19.10 | 0.353 |
| Isoaromadendrene epoxide | 19.23 | 0.108 |
| 1,3-Cyclopentadiene, 5,5-dimethyl-1,2-Dipropyl- | 19.35 | 0.243 |
| Alloaromadendrene oxide-(1) | 19.83 | 0.039 |
| Oxacyclohexadecan-2-one, 16-methyl-, (-)- | 19.94 | 0.714 |
| Hexadecanoic acid, methyl ester | 20.54 | 0.043 |
| Aromandendrene | 20.78 | 0.026 |
| 3-Octyne, 2,2,7-trimethyl- | 21.21 | 0.043 |
| Sulfurous acid, 2-propyl tridecyl ester | 24.77 | 0.027 |
| Bis(2-ethylhexyl) phthalate | 25.65 | 0.127 |
| 3-[N-(2'-Propen-1'-yl)carbamoyl]-1-(t-butyl)-5-nitropyridin-2(1H)-one | 26.07 | 44.83 |
| Eicosane | 26.33 | 0.042 |
| 3-Quinolinecarboxylic acid, 6,8-difluoro-4-hydroxy-, ethyl ester | 27.07 | 0.028 |
| 2,4,6-Cycloheptatrien-1-one, 3,5-bis-trimethylsilyl- | 27.78 | 0.029 |
| Cyclotrisiloxane, hexamethyl- | 28.48 | 0.034 |
| 3-Quinolinecarboxylic acid, 6,8-difluoro-4-hydroxy-, ethyl ester | 29.14 | 0.031 |

RT= retention time

**Table 7**. Artichoke (*Cynara scolimus*) oil composition determined by GC-MS

| **Component** | RT | % |
| --- | --- | --- |
| 2-Pinene | 7.25 | 0.136 |
| 2,2-dimethyl-3-methylene-bicyclo [2.2.1]heptane | 7.54 | 0.052 |
| 2(10)-Pinene | 8.07 | 0.642 |
| 3-Carene | 8.69 | 0.502 |
| 2-Oxabicyclo[2.2.2]octane, 1,3,3-trimethyl- | 9.06 | 0.265 |
| 1,3,3-trimethyl-2-nor-bornan-ol(without stereochemistry) | 10.49 | 0.057 |
| Bicyclo[2.2.1]heptan-2-ol, 1,7,7-trimethyl-, (1S-endo)- | 11.33 | 0.096 |
| γ -Terpinene | 11.74 | 0.163 |
| Cyclohexene, 4-ethenyl-4-methyl-3-(1-methylethenyl)-1-(1-methylethyl)-, (3R-trans)- | 13.82 | 0.197 |
| Tricyclo[4.4.0.02,7]dec-3-ene, 1,3-dimethyl-8-(1-methylethyl)-, stereoisomer | 13.99 | 0.052 |
| 1,2,4-Metheno-1H-indene, octahydro-1,7a-dimethyl-5-(1-methylethyl)-, [1S-(1α,2α,3aβ,4α,5α,7aβ,8S*)]- | 14.25 | 0.078 |
| Copaene | 14.36 | 0.412 |
| 2,4-Diisopropenyl-1-methyl-1-vinyl-cyclohexane | 14.56 | 0.274 |
| (1R,5R)-2-Methyl-5-((R)-6-methylhept-5-en-2-yl)bicyclo[3.1.0]hex-2-ene | 14.69 | 0.065 |
| cis-alpha-Bergamotene | 14.85 | 0.079 |
| Caryophyllene | 14.97 | 2.415 |
| trans-alpha-Bergamotene | 15.11 | 0.819 |
| 2,6,10-Dodecatrien-1-ol, 3,7,11-trimethyl-, (Z,E)- | 15.26 | 0.066 |
| (1R,4R,4aS,8aR)-4,7-Dimethyl-1-(prop-1-en-2-yl)-1,2,3,4,4a,5,6,8a-octahydronaphthalene | 15.34 | 0.275 |
| α -Humulene | 15.40 | 0.222 |
| (1R,4R,4aS,8aR)-4,7-Dimethyl-1-(prop-1-en-2-yl)-1,2,3,4,4a,5,6,8a-octahydronaphthalene | 15.46 | 0.957 |
| Benzene, 1-(1,5-dimethyl-4-hexenyl)-4-methyl- | 15.70 | 7.21 |
| 1,3-Cyclohexadiene, 5-(1,5-dimethyl-4-hexenyl)-2-methyl-, [S-(R*,S*)]- | 15.85 | 4.635 |
| α -Gurjunene | 15.92 | 0.802 |

RT= retention time

**Table 7**. Artichoke (*Cynara scolimus*) oil composition determined by GC-MS

| **Component** | RT | % |
| --- | --- | --- |
| γ -Muurolene | 16.50 | 0.135 |
| 3-Methyl-2-(2-oxopropyl)furan | 16.69 | 0.071 |
| 1H-3a,7-Methanoazulene, 2,3,4,7,8,8a-hexahydro-3,6,8,8-tetramethyl-, [3R-(3α,3aβ,7β,8aα)]- | 16.93 | 0.057 |
| Hexadecane | 17.02 | 0.356 |
| Bicyclo[3.1.1]hept-2-en-6-ol, 2,7,7-trimethyl-, acetate, [1S-(1α,5α,6β)]- | 17.25 | 0.045 |
| 1H-3a,7-Methanoazulene, octahydro-3,8,8-trimethyl-6-methylene-, [3R-(3α,3aβ,7β,8aα)]- | 17.49 | 0.061 |
| Hentriacontane | 17.58 | 0.155 |
| 2-Methyl-Z-4-tetradecene | 17.84 | 0.043 |
| ar-Turmerone | 17.89 | 0.576 |
| Heptadecane | 18.16 | 0.231 |
| Pentadecane, 2,6,10,14-tetramethyl- | 18.22 | 0.224 |
| Curlone | 18.31 | 0.469 |
| Octadecane | 19.24 | 0.112 |
| Pentacosane | 19.34 | 0.168 |
| Bis(2-ethylhexyl) phthalate | 25.65 | 0.159 |
| Glutaric acid, di(2-isopropoxyphenyl) ester | 25.86 | 0.042 |
| Bis(2-ethylhexyl) phthalate | 26.04 | 68.192 |

RT= retention time

**Table 8**. Ginger (*Zingiber officinale*) oil composition determined by GC-MS

| **Component** | RT | % |  | RT | % |
| --- | --- | --- | --- | --- | --- |
| Camphene | 4.83 | 0.88 | α-Cedro | 20.55 | 0.3 |
| Linalool | 8.11 | 1.31 | β-Atlantol | 20.78 | 0.21 |
| Borneol | 9.91 | 2.92 | 10-epi-γ-Eudesmol | 21.1 | 1.06 |
| α-Terpineol | 10.52 | 1.18 | γ-Eudesmol | 21.31 | 0.04 |
| Z-Citral | 11.8 | 1.11 | Valerianol | 21.42 | 0.03 |
| E-Citral | 12.57 | 1.66 | Hinesol | 21.49 | 1.2 |
| 2-Undecanone | 13.12 | 0.77 | Agarospirol | 21.56 | 0.09 |
| β-Caryophillene | 16.48 | 0.76 | β-Eudesmol | 22.01 | 0.5 |
| α-Curcumene | 18.11 | 15.23 | α-Eudesmol | 22.07 | 0.5 |
| β-Selinene | 18.16 | 0.41 | Khusinol | 22.25 | 0.08 |
| α-Zingiberene | 18.37 | 28.25 | α-Bisabolol | 22.36 | 0.08 |
| cis-γ-Cadinene | 18.50 | 5.24 | epi-α-Bisabolol | 22.78 | 0.2 |
| trans-γ-Cadinene | 18.7 | 11.88 | E-Nuciferal | 22.97 | 1.5 |
| β-Curcumene | 18.77 | 0.32 | Guaiol acetat | 23.06 | 0.32 |
| Zonarene | 18.83 | 0.48 | trans-Farnesol | 23.32 | 0.14 |
| β-Sesquiphellandrene | 19.13 | 15.65 | Z-Nuciferol | 23.45 | 0.1 |
| trans-γ-Bisabolene | 19.24 | 0.41 | cis-Lanceol | 23.63 | 0.1 |
| β-Vetivenene | 19.46 | 0.06 | β-Bisabolen-12-ol | 24.01 | 0.16 |
| Elemol | 19.64 | 0.05 | Cuparopheno | 24.11 | 0.85 |
| Z-Nerolidol | 19.69 | 0.07 | Benzyl salicylate | 26.63 | 0.15 |
| Spatulenol | 19.91 | 2.89 | Palmitic acid | 28.25 | 0.19 |
| Tumerol | 20.3 | 0.34 | α-Springene | 28.77 | 0.15 |
| Viridiflorol | 20.46 | 0.08 | Shogaol | 34.35 | 0.1 |

RT= retention time, [73]

**Table 9.** Turmeric (*Curcuma longa*) and garlic (*Allium sativum*) oil composition determined by GC-MS

| **Tumeric** | RT | % | **Garlic** | RT | % |
| --- | --- | --- | --- | --- | --- |
| α-Pinene | 3.34 | 1.5 | 1,2-Dithiolane | 7.97 | 0.25 |
| α-Phellandrene | 4.32 | 1.43 | Allyl sulfide | 8.40 | 6.65 |
| Limonene | 4.72 | 0.2 | Disulfide,methyl 2-propeny | 11.19 | 0.07 |
| Eucalyptol | 4.79 | 1 | Methyl allyl sulfide | 13.52 | 7.83 |
| Terpinolene | 5.83 | 0.26 | Diallyldisulphide | 19.72 | 41.33 |
| Terpinen-4-ol | 7.80 | 0.08 | 3-Vinyl-1,2-dithiocyclohex-4-ene | 25.44 | 1.66 |
| Benzenemethanol | 8 | 0.12 | 3-Vinyl-1,2-dithiocyclohex-5-ene | 26.77 | 3.74 |
| Terpineol | 8.11 | 0.14 | Diallyltrisulfide | 30.65 | 28.82 |
| Anethole | 10.38 | 0.31 | Terisulfide,di-2-propenyl | 30.83 | 0.33 |
| Phenol | 10.59 | 0.18 | 7-chloro-s-triazola(1,5-c)pyrimidin-5(1h)one | 34.24 | 2.18 |
| Caryophyllene | 13.68 | 1.7 | 2-(2-Thia-4-pentenyl)-1-thia-cyclohex-5-ene | 39.99 | 0.12 |
| Humulene | 14.48 | 0.7 | Diallyltetrasulphide | 40.99 | 3.93 |
| Benzene | 15.18 | 4.18 | 4,5-Dimethyl-thiazole | 49.15 | 0.61 |
| Zingiberene | 15.29 | 8.30 | 1,3-Butadiene,3-methyl-1,1-bis(methylthio) | 50.08 | 0.30 |
| Cyclohexadiene | 15.46 | 2.9 |  |  |  |
| β-Bisabolene | 16.25 | 0.91 |  |  |  |
| Tumerone | 17.37 | 40 |  |  |  |
| Curlon | 18.77 | 34 |  |  |  |

RT= retention time, [74, 75]

**Table 10.** Black pepper (*Piper nigrum*) oil composition determined by GC-MS

| **Component** | RT | % |  | RT | % |
| --- | --- | --- | --- | --- | --- |
| α-phellandrene | 2.31 | 0.40 | 4-terpineol | 6.36 | 0.15 |
| Camphene | 2.69 | 0.13 | Delta-Elemene | 9.20 | 0.65 |
| Sabinene | 2.75 | 2.98 | Copaene | 9.93 | 0.70 |
| β-pinene | 2.86 | 8.00 | β-Elemene | 10.14 | 1.15 |
| α-pinene | 2.97 | 6.32 | α-Bergamotene | 10.54 | 0.62 |
| 3-Carene | 3.18 | 7.08 | Caryophyllene | 10.75 | 19.12 |
| Trifluoromethanesulfenyl fluoride | 3.25 | 2.14 | α-Caryophyllene | 11.26 | 1.83 |
| (+)-Camphene | 3.30 | 8.44 | α-Curcumene | 11.56 | 0.41 |
| 0-Cymene | 3.37 | 0.63 | Cedrene | 11.64 | 0.64 |
| Limonene | 3.58 | 9.74 | β-Bisabolene | 11.96 | 1.65 |
| β-phellandrene | 3.62 | 0.94 | (+)-delta-Cadinene | 12.14 | 0.32 |
| γ -terpinene | 3.99 | 0.11 | Caryophyllene oxide | 13.16 | 1.78 |
| Terpinolene | 4.41 | 0.30 | Spathulenol | 13.72 | 0.44 |
| Terpinolene | 4.50 | 0.36 | α-Bisabolol | 14.49 | 0.41 |
| Linalol | 4.58 | 0.15 | 2-Undecanone | 12.64 | 0.10 |

RT= retention time, [76]
