# Supplementary material for: Application of various mixtures of medicinal herbs in the diet of laying hens: Evaluating preventive approach of fatty liver syndrome
Source: PLoS One. 2025 Oct 24;20(10):e0330363. doi: 10.1371/journal.pone.0330363 (PMC12551855; doi:10.1371/journal.pone.0330363)
Supplement: Attachment — Submitted filename: PONE-D-25-15950_reviewer.pdf [file pone.0330363.s004.pdf]

# PLOS ONE

## Various mixtures of medicinal herbs in the diet of laying hens: Evaluating preventive approach of fatty liver syndrome

--Manuscript Draft--

| | |
|---|---|
| **Manuscript Number:** | PONE-D-25-15950 |
| **Article Type:** | Research Article |
| **Full Title:** | Various mixtures of medicinal herbs in the diet of laying hens: Evaluating preventive approach of fatty liver syndrome |
| **Short Title:** | Dietary medicinal herbs to prevent fatty liver syndrome in laying hens |
| **Corresponding Author:** | Mehran Torki<br>Razi University<br>Kermanshah, IRAN, ISLAMIC REPUBLIC OF |
| **Keywords:** | Egg Production; Fatty Liver; Laying Hen; Medicinal Herb |
| **Abstract:** | Background<br><br>Fatty liver hemorrhagic syndrome (FLHS) is a major health issue in laying hens, which is associated with reduced productive performance and increased mortality. Herbal additives have been explored as a dietary intervention to prevent the occurrence of FLHS. In the present study, the effect of three herbal mixes have been examined in preventing FLHS in laying hens. The study involved 384 LSL-Lohmann laying hens from the age of 65 to 77 weeks. Hens were randomly distributed among 8 treatments, each consisted of 8 replicates (cages) of six birds in a 2 × 4 factorial layout. There were two basal diets including a standard diet (SD; having commercial norms of energy and protein) and a challenge diet (CD; high-energy and low-protein diet) as well as four dietary interventions (three mixes of herbs and a control with no herbal additives). Mix 1 consisted of turmeric, fumitory, green tea, and milk thistle. Mix 2 included lemon, black pepper, sumac, and chicory. Mix 3 contained garlic, artichoke, ginger, and shallot. Productive performance, blood variables and liver lesion scores were examined.<br><br>Results<br><br>The results demonstrated that the CD decreased feed intake (FI), egg production (EP), and egg weight (EW), whereas it increased feed conversion ratio (FCR) from Week 5 onward. Feeding the CD resulted in higher circulatory levels of aspartate aminotransferase (AST) and triglycerides (TG), and a higher frequency of severe livers scores. Inclusion of Mix 2 significantly restores these responses. Inclusion of herbal mixes while feeding the CD did not make a significant effect on the number of heterophiles and lymphocytes and hemoglobin (Hb) concentration.<br><br>Conclusion<br><br>Feeding a CD could result in poor productive performance and it was associated with a higher frequency of liver pathological lesions. Supplementing the CD with Mix 2 resulted in the most noticeable improvements on all parameters measured. The combination of lemon, black pepper, sumac, and chicory was found to be effective dietary intervention to prevent FLHS in laying hens. |
| **Order of Authors:** | Mohammadreza Khodaei, PhD |
| | Mehran Torki |
| | Fariborz Khajali |
| | Iraj Karimi, PhD |
| **Additional Information:** | |
| **Question** | **Response** |

Powered by Editorial Manager® and ProduXion Manager® from Aries Systems Corporation

| | |
|---|---|
| **Financial Disclosure**<br><br>Enter a financial disclosure statement that describes the sources of funding for the work included in this submission. Review the submission guidelines for detailed requirements. View published research articles from *PLOS ONE* for specific examples.<br><br>This statement is required for submission and **will appear in the published article** if the submission is accepted. Please make sure it is accurate.<br><br>**Funded studies**<br>Enter a statement with the following details:<br>• Initials of the authors who received each award<br>• Grant numbers awarded to each author<br>• The full name of each funder<br>• URL of each funder website<br>• Did the sponsors or funders play any role in the study design, data collection and analysis, decision to publish, or preparation of the manuscript?<br><br>Did you receive funding for this work? | The author(s) received no specific funding for this work. |
| **Competing Interests**<br><br>Use the instructions below to enter a competing interest statement for this submission. On behalf of all authors, disclose any competing interests that could be perceived to bias this work—acknowledging all financial support and any other relevant financial or non-financial competing interests.<br><br>This statement is required for submission and **will appear in the published article** if the submission is accepted. Please make sure it is accurate and that any funding sources listed in your Funding Information later in the submission form are also declared in your Financial Disclosure statement. | The authors have declared that no competing interests exist. |

| | |
|---|---|
| View published research articles from *PLOS ONE* for specific examples.<br><br>**NO authors have competing interests**<br><br>Enter: *The authors have declared that no competing interests exist.*<br><br>**Authors with competing interests**<br><br>Enter competing interest details beginning with this statement:<br><br>*I have read the journal's policy and the authors of this manuscript have the following competing interests: [insert competing interests here]*<br><br>* typeset | |
| **Ethics Statement**<br><br>Enter an ethics statement for this submission. This statement is required if the study involved:<br><br>• Human participants<br>• Human specimens or tissue<br>• Vertebrate animals or cephalopods<br>• Vertebrate embryos or tissues<br>• Field research<br><br>Write "N/A" if the submission does not require an ethics statement.<br><br>General guidance is provided below. Consult the submission guidelines for detailed instructions. **Make sure that all information entered here is included in the Methods section of the manuscript.** | The Animal Ethics Committee of Razi University, Kermanshah, Iran approved guidelines were followed for all experimental protocols (IR.RAZI.REC.1400.051). These guidelines adhered to the EU standards for the protection of animals and/or feed legislation |

| | |
|---|---|
| **Format for specific study types**<br><br>**Human Subject Research (involving human participants and/or tissue)**<br>• Give the name of the institutional review board or ethics committee that approved the study<br>• Include the approval number and/or a statement indicating approval of this research<br>• Indicate the form of consent obtained (written/oral) or the reason that consent was not obtained (e.g. the data were analyzed anonymously)<br><br>**Animal Research (involving vertebrate animals, embryos or tissues)**<br>• Provide the name of the Institutional Animal Care and Use Committee (IACUC) or other relevant ethics board that reviewed the study protocol, and indicate whether they approved this research or granted a formal waiver of ethical approval<br>• Include an approval number if one was obtained<br>• If the study involved *non-human primates*, add *additional details* about animal welfare and steps taken to ameliorate suffering<br>• If anesthesia, euthanasia, or any kind of animal sacrifice is part of the study, include briefly which substances and/or methods were applied<br><br>**Field Research**<br><br>Include the following details if this study involves the collection of plant, animal, or other materials from a natural setting:<br>• Field permit number<br>• Name of the institution or relevant body that granted permission | |
| **Data Availability**<br><br>Authors are required to make all data underlying the findings described fully available, without restriction, and from the time of publication. PLOS allows rare exceptions to address legal and ethical concerns. See the PLOS Data Policy and FAQ for detailed information. | Yes - all data are fully available without restriction |

| | |
|---|---|
| A Data Availability Statement describing where the data can be found is required at submission. Your answers to this question constitute the Data Availability Statement and **will be published in the article**, if accepted.<br><br>**Important:** Stating 'data available on request from the author' is not sufficient. If your data are only available upon request, select 'No' for the first question and explain your exceptional situation in the text box.<br><br>Do the authors confirm that all data underlying the findings described in their manuscript are fully available without restriction? | |
| **Describe where the data may be found in full sentences. If you are copying our sample text, replace any instances of XXX with the appropriate details.**<br><br>• If the data are **held or will be held in a public repository**, include URLs, accession numbers or DOIs. If this information will only be available after acceptance, indicate this by ticking the box below. For example: *All XXX files are available from the XXX database (accession number(s) XXX, XXX.).*<br>• If the data are all contained **within the manuscript and/or Supporting Information files**, enter the following: *All relevant data are within the manuscript and its Supporting Information files.*<br>• If neither of these applies but you are able to provide **details of access elsewhere**, with or without limitations, please do so. For example:<br><br>*Data cannot be shared publicly because of [XXX]. Data are available from the XXX Institutional Data Access / Ethics Committee (contact via XXX) for researchers who meet the criteria for access to confidential data.*<br><br>*The data underlying the results presented in the study are available from (include the name of the third party* | All relevant data are within the manuscript and its Supporting Information files. |

| | |
|---|---|
| *and contact information or URL).*<br>• This text is appropriate if the data are owned by a third party and authors do not have permission to share the data.<br><br>* typeset | |
| Additional data availability information: | |

Manuscript Click here to access/download;Manuscript;Manuscript.docx



# Various mixtures of medicinal herbs in the diet of laying hens: Evaluating preventive approach of fatty liver syndrome

Mohammadreza Khodaei[1], Mehran Torki[1*], Fariborz Khajali[2], Iraj Karimi[3]

[1] Department of Animal Science, College of Agriculture and Natural Resources, Razi University, Kermanshah, Iran, Postal Code: 67156-85423

[2] Department of Animal Science, Shahrekord University, Shahrekord, Iran, Postal Code: 88186-34141

[3] Department of Pathology, Faculty of Veterinary Medicine, Shahrekord University, Shahrekord, Iran

[*] Corresponding author: torki@razi.ac.ir

**Running title:** Dietary medicinal herbs to prevent fatty liver syndrome in laying hens

## Abstract

**Background**: Fatty liver hemorrhagic syndrome (FLHS) is a major health issue in laying hens, which is associated with reduced productive performance and increased mortality. Herbal additives have been explored as a dietary intervention to prevent the occurrence of FLHS. In the present study, the effect of three herbal mixes have been examined in preventing FLHS in laying hens. The study involved 384 LSL-Lohmann laying hens from the age of 65 to 77 weeks. Hens were randomly distributed among 8 treatments, each consisted of 8 replicates (cages) of six birds in a 2 × 4 factorial layout. There were two basal diets including a standard diet (SD; having commercial norms of energy and protein) and a challenge diet (CD; high-energy and low-protein diet) as well as four dietary interventions (three mixes of herbs and a control with no herbal additives). Mix 1 consisted of turmeric, fumitory, green tea, and milk thistle. Mix 2 included lemon, black pepper, sumac, and chicory. Mix 3 contained garlic, artichoke, ginger, and shallot. Productive performance, blood variables and liver lesion scores were examined.

**Results**: The results demonstrated that the CD decreased feed intake (FI), egg production (EP), and egg weight (EW), whereas it increased feed conversion ratio (FCR) from Week 5 onward. Feeding the CD resulted in higher circulatory levels of aspartate aminotransferase (AST) and triglycerides (TG), and a higher frequency of severe livers scores. Inclusion of Mix 2 significantly restores these responses. Inclusion of herbal mixes while feeding the CD did not make a significant effect on the number of heterophiles and lymphocytes and hemoglobin (Hb) concentration.

**Conclusion**: Feeding a CD could result in poor productive performance and it was associated with a higher frequency of liver pathological lesions. Supplementing the CD with Mix 2 resulted in the most noticeable improvements on all parameters measured. The combination of lemon, black pepper, sumac, and chicory was found to be effective dietary intervention to prevent FLHS in laying hens.

**Keywords:** Egg Production; Fatty Liver; Laying Hen; Medicinal Herb

## Background

In birds, lipids are primarily synthesized in the liver. A typical layer diet contains only 2-4% lipids, while body weight gain and egg formation require much higher lipids to be formed [1]. The liver accounts for supplying lipids for these vital functions. Consequently, the liver of laying hens is naturally dealt with an enormous amount of lipids [2, 3]. This situation predisposes laying hens to fatty liver hemorrhagic syndrome (FLHS).

FLHS is a non-infectious disease characterized by excessive fat build-up in the liver and abdominal cavity. This fat accumulation can lead to the rupture of hepatocytes, internal bleeding, and sudden death in birds [4, 5]. The syndrome is more commonly observed in laying hens housed in cage systems [5, 6]. Flocks affected by FLHS experience significant economic losses due to decreased egg production and increased mortality rates [4, 7]. During necropsy, birds that died from FLHS exhibit enlarged, soft, yellow, and fatty livers. This compromised state makes the liver more susceptible to tearing and bleeding [3, 5].

The exact cause of FLHS remains unclear, but several factors are thought to be involved including nutritional factors (excess dietary energy, fungal toxins, and anti-nutritional substances) [2, 8, 9], genetic

predisposition, and environmental factors (ambient temperature and rearing system) [5, 10]. It is likely that a combination of these factors may contribute to the development of FLHS. Several nutritional strategies have been investigated to reduce hepatic lipid biosynthesis and prevent FLHS. One promising approach involves the use of medicinal plants.

Medicinal plants contain bioactive compounds with various health attributes. Sumac (*Rhus coriaria*) is rich in phenolic compounds (flavonoids and anthocyanins) [11]. Lemon (*Citrus limon*) contains vitamin C, flavonoids, and limonoids [12]. Black pepper (*Piper nigrum*) is known for its piperine alkaloids [13]. Chicory (*Cichorium intybus L.*) is rich in inulin and sesquiterpenes [14]. Milk thistle *(Silybum marianum*) contains a considerable content of silymarin, a flavonoid [15]. Garlic and shallot (*Allium sativum, Allium cepa*) possess allicin, an organosulfur compound [16]. Artichoke (*Cynara scolimus*) is known for its inulin and oligofructose content, which are beneficial fibers [17]. Green tea (*Camellia sinensis*) contains catechins, a group of flavonoids [18]. Turmeric (*Curcuma longa*) bioactive compound is curcumin [19] and bioactive compounds in fumitory (*Fumaria officinalis*) are protopine and fumaric acid [20]. Ginger (*Zingiber officinale*) consists of gingerol, gingerdiol, and gingerdione [21].

Bioactive compounds in these medicinal plants have shown antioxidant, anti-inflammatory, and lipid-lowering properties [22]. Although the use of individual medicinal herbs has been examined in laying hens, combinations of them have not been studied particularly with regard to FLHS. Thus, the objectives of the present study were to develop an appropriate formulation of herbal additives in laying hen diets to evaluate productive responses, blood parameters, and the development of FLHS.

## Methods

### Experimental design and dietary treatments

The Animal Ethics Committee of Razi University, Kermanshah, Iran approved guidelines were followed for all experimental protocols (IR.RAZI.REC.1400.051). These guidelines adhered to the EU standards for the protection of animals and/or feed legislation. The total number of one thousand fertile eggs were bought from a local hatchery company (Toyor-Barekat), and set in the laboratory incubator in Animal Science Department. The hatched chicks were then reared until the starting of the experimental trail based on the

company catalogue. Finally, at the age of 65 weeks, the total number of 384 Lohmann LSL-Lite laying hens were randomly selected from this larger herd and distributed between 64 wire cages with dimensions of 45 × 45 × 45 cm, with six birds per cage. A 16-hour light/8-hour dark lighting schedule was implemented. The housing temperature was set at 24°C ± 1°C, and the relative humidity was kept at about 40%. Hens had free access to feed and water throughout the experiment. Two basal diets were formulated for the experiment: a standard diet (SD) formulated according to the strain's recommended guidelines (2500 kcal/kg AME and 15% crude protein) and a challenge diet (CD) designed to induce FLHS included high energy and low protein contents (3100 kcal/kg AME and 12% crude protein). Within each basal diet, there were four dietary interventions including three herbal mixes along with a control with no herbal additives. Mix 1 included turmeric, fumitory, green tea, and milk thistle. Mix 2 contained lemon, black pepper, sumac, and chicory. Mix 3 comprised garlic, artichoke, ginger, and shallot. The experimental diets and their chemical composition are presented in Table 1. The experimental diets were fed to laying hens for two weeks in order to adapt to new diets before running into the experimental trial. The adaptation period was considered to minimize the error effect caused by the changes in the diets. The experimental trial lasted for 12 weeks (weeks 65 through 77).

## Measurements

### Gas chromatography-mass spectrometry (GC-MS)

The volatile compounds within the plant samples were identified using a headspace (HS) sampling technique followed by GC-MS analysis. A qualitative GC-MS analysis was performed with an HP 6890 gas chromatograph interfaced with an HP 5975C Mass Selective Detector (Agilent Technologies, Foster City, CA, USA). The analysis employed electron ionization (EI) mode at 70 eV and followed the procedure described by Naghneh et al. [23]. The composition of herbs has shown in Additional file 1.

### Productive performance

Egg production and egg weight were recorded on a daily basis, and the data were used to calculate hen-day egg production (EP) and egg weight (EW). Moreover, feed conversion ratio (FCR) was calculated based on feed intake (FI) and egg performance data.

**Blood parameters**

At weeks 65 (the start), 70 (the middle) and 77 (the end of trial), two laying hen from each replicate (16 per treatment) was randomly selected and 3 ml of blood was collected in a test tube without any anticoagulant. The blood samples were centrifuged at 3000 rpm for 10 minutes and the resulting sera were stored at -20°C until laboratory tests. The following parameters were determined according to the manufacturer's guidelines: aspartate aminotransferase (AST), total protein (TP), total triglyceride (TG), total cholesterol (TC), low-density cholesterol (LDL), high-density cholesterol (HDL), uric acid (UA), and hemoglobin (Hb). In addition, a smear of blood was prepared from each blood sample and sent to the laboratory to count heterophiles and lymphocytes.

**Hepatic histopathology and scoring system**

During the experimental period, all mortality was weighed and necropsied, and the incidence of FLHS was assessed in all treatment groups. At the end of trial (Week 77), a single bird from each cage (8 per treatment) were randomly selected and the selected laying hens were anaesthetised via inhalation of 60% CO2 and then sacrificed by cervical dislocation. $CO_2$ was delivered from compressed gas canister and delivered using a gradual fill method with a displacement rate of 60% via the chamber volume per minute using of a flowmeter. Their livers were harvested and dissected for further analysis. To assess histological examinations, liver tissues were fixed in 10% buffered formalin and processed by a microtome. These sections were then stained with hematoxylin and eosin (H&E) and observed under a light microscope [24]. A four-point scoring system was employed to evaluate the severity of fatty liver FLHS in the chicken livers, based on the presence and extent of acentric nuclei within hepatocytes. Acentric nuclei are displaced from their central position due to the accumulation of fat droplets within the cells. Scores ranged from 1

(indicating normal liver pathology with centrally located nuclei) to 4 (representing severe FLHS with extensive acentric nuclei), with 2 and 3 signifying mild and moderate degrees of acentricity, respectively (Figure 1).

**Statistical analysis**

The study was conducted in a completely randomized design using a 2×4 factorial layout. Data were subjected to the GLM procedures of SAS 9.4 software [25] according to the following model:

$$Y_{ijk} = \mu + A_i + B_j + ABij + e_{ijk}$$

where $Y_{ijk}$ = observed value for a particular trait, μ= overall mean, $A_i$ = effect of the basal diet (SD and CD), $B_j$ = effect of the herbal mixture, $AB_{ij}$ = the respective interaction of ith and jth levels of dietary SDCD and herbal additives, and $e_{ijk}$ = random error associated with the ijkth recording. The treatment means were separated by Duncan's multiple-range test at $P \leq 0.05$ statistical level. The effects of the main factors were not considered, whenever the interaction was significant. The non-parametric method of Kruskal-Wallis test was used to analyze liver histopathology scores [25].

## Results

**Laying performance**

Tables 2, 3, 4, and 5 present the effects of dietary interventions on FI, EP, EW, and FCR of laying hens, respectively. Although the difference between the treatments was not significant with respect to FI during the first 4 weeks of the experiment, the main effects of SD and CD, as well as the main effects of herbal additives in weeks 5 and 6 were significant (Table 2). Feeding laying hens with the CD reduced FI as opposed to the SD ($P < 0.05$). Additionally, Mix 2 had the highest FI compared to other groups ($P < 0.05$). From week 7 through 12, a notable interaction effect of the experimental treatments was observed for FI of laying hens ($P < 0.05$). Laying hens fed with SD with or without herbal Mixes had higher FI, followed by the CD + Mix 2, which were superior to other treatments (Table 2).

EP rate was not affected by the experimental treatments during the first 4 weeks of the trial (Table 3). From Week 5 onward, the interaction effects of SD and CD plus herbal mixes on EP were significant ($P < 0.05$). Throughout the trial, the CD and the CD with herbal mixes showed reduced EP compared to the SD and the SD with herbal mixes. The except was CD + Mix 2, which had no significant difference with SD ($P > 0.05$).

No significant difference was observed with respect to EW of laying hens ($P > 0.05$), except for the interaction effects of the first week and the main effect of herbal additives in week 3 ($P < 0.05$) (Table 4). In the first week, the group fed with SD had more EW compared to the SD + Mix 1, and in the third week Mix 2 had a higher EW relative to the control group ($P < 0.05$). In addition, the main effects of SD and CD were significant from weeks 5 through 12, so that the laying hens fed with CD had lower EW than SD ($P < 0.05$). In weeks 5 to 9, the main effects of herbal additives on EW were significant, and the diet contained Mix 2 and Mix 3 had higher EW than the control diet ($P < 0.05$).

FCR was not affected by the experimental treatments between weeks 1 to 4 and weeks 9 to 12 ($P > 0.05$) (Table 5). The interaction effects of SD and CD with herbal additives were significant during weeks 5 to 8 ($P < 0.05$). In week 5, the lowest FCR was related to the SD, SD + Mix 3, and CD + Mix 2, which were significantly different from CD, CD + Mix 1, and CD + Mix 3 ($P < 0.05$). In week 6, the lowest FCR belonged to the CD+ Mix 2 and SD diets, and the highest FCR was related to CD, CD + Mix 4, and CD + Mix 3. In week 7, the CD + Mix 2, SD, SD + Mix 3, and CD had the lowest FCR and the CD + Mix 1 and CD + Mix 3 had the highest. Moreover, CD + Mix 2 and CD + Mix 3 had the lowest and highest FCR, respectively, when obtained at week 8 ($P < 0.05$).

**Hematological criteria**

Tables 6, 7, and 8 show hematological criteria of laying hens fed with herbal additives in both SD and CD at the beginning, middle, and end of the experimental period. Feeding the CD resulted in elevated levels of AST compared to the standard diet at the middle and the end of trial. The interaction effects of dietary treatments on AST enzyme in the middle and the end of trial were significant ($P < 0.05$) so that the lowest

AST level was related to the laying hens fed with SD + Mix 3 and CD + Mix 2 and the highest was related to the CD, CD + Mix 1 (Table 6).

The interaction effects of CD and SD with herbal additives on the concentration of TP were significant at the end of trial ($P < 0.05$) (Table 6). In the middle of trial, the CD + Mix 2 had the highest TP concentration, and the CD and the CD + Mix 1 had the lowest TP concentration. The CD + Mix 2, SD + Mix 2, and SD + Mix 3 had the highest TP, and the SD, CD, and CD + Mix 1 had the lowest at the end of trial.

As for UA, in the middle and the end of experiment, the interaction effects of experimental treatments were significant ($P < 0.05$) (Table 6). In general, the SD without additives had the highest amount of blood UA, which was significantly different from SD + Mix 2, SD + Mix 3, and CD + Mix 2 in the middle of trial and SD + Mix 1, SD + Mix 2, SD + Mix 3, and CD + Mix 2, at the end of trial ($P < 0.05$). The lowest amount of UA was seen in the SD + Mix 2 and SD + Mix 3.

The main effects of SD and CD on TG concentration in the second sampling and its interaction effect in the third sampling were significant ($P < 0.05$) (Table 7). Based on the results, feeding CD to laying hens increased TG concentration when compared to the SD ($P < 0.05$). Likewise, in the third sampling, birds fed with CD, CD + Mix 1, and CD + Mix 3 had the highest amount of TG, and SD + Mix 1 had the lowest.

In the second and third sampling, the interaction effects of dietary treatments on TC concentration were significant ($P < 0.05$) (Table 7). The highest amount of TC in the second sampling was observed in layers fed with CD + Mix 3 and the lowest in the diet of laying hens fed with SD + Mix 3. In the third sampling, the highest concentration of TC was from feeding CD + Mix 1 and CD + Mix 3, and the lowest was observed by feeding SD+ Mix 3.

The effect of treatments on HDL concentration exhibited a statistically significant interaction effect at the second and third sampling, so that SD + Mix 3 had the highest HDL concentration compared to the other treatments ($P < 0.05$) (Table 7). As for LDL, the main effect of SD and CD in the second sampling was significant, and CD increased the concentration of LDL compared to the SD ($P < 0.05$) (Table 7).

The interaction effects of diets showed a significant effect on blood Hb concentration and heterophile and lymphocyte count in the second and third sampling ($P < 0.05$) (Table 8). In the second sampling, the CD +

Mix 2 had the highest, and SD + Mix 2 and SD+ Mix 3 had the lowest amount of Hb. In the third sampling, the highest Hb was seen in CD and CD + Mix 1 and the lowest in SD + Mix 2 and SD + Mix 3. In terms of the number of heterophiles in the second and third sampling, the highest number was seen in SD + Mix 1 and the lowest in SD + Mix 2 and SD + Mix 3. The highest number of lymphocytes in the second sampling was related to SD, SD + Mix 1, and all of the CDs, and the lowest was found in SD + Mix 2. Moreover, in the third sampling, CD + Mix 1 and SD + Mix 2 had the highest and lowest number of lymphocytes, respectively ($P < 0.05$).

**Hepatic histopathology**

Figure 2 illustrates the effects of various herbal mixes on the frequency of liver histopathology scores of laying hens. Statistical analysis revealed a significant difference ($P < 0.05$) in these scores between the treatment groups. Notably, hens fed diets containing SD + Mix 2 and CD + Mix2 exhibited significantly lower liver scores compared to those fed CD without additive. The difference between other experimental treatments was not significant ($P > 0.05$).

**Discussion**

This study investigated the effects of various medicinal plant mixtures on laying hen performance, blood chemistry, and liver health. These mixtures were provided alongside a SD or a CD. Among the tested mixtures, hens fed a CD supplemented with Mix 2 (containing lemon, black pepper, sumac, and chicory) exhibited significant improvements in EP and FCR, while also alleviating negative effects on the liver. The chemical composition of these herbal mixtures can vary depending on the plant species, growth conditions, and extraction methods. The GC-MS analysis of the herbal plant essential oils revealed a large portion of the identified compounds in each oil belonged to a group of phthalates. Phthalides are a class of secondary metabolites with a wide range of pharmacological activities including anti-inflammatory and antimicrobial activities [26]. Moreover, most essential oils contained terpenes, a well-studied group known for their anti-inflammatory and antioxidant effects [27]. Examples identified in this study include caryophyllene, beta-

bisabolene, limonene, α-copaene, and several others. Additionally, garlic and shallot displayed the presence of organosulfur compounds, another class of potentially beneficial natural products [16]. Interestingly, curcumin, a curcuminoid with recognized health benefits, was the dominant component in turmeric essential oil [19].

In this study the CD was formulated with high energy and low protein content to induce FLHS [28, 29]. Feeding the CD decreased FI, EW, and EP rate from week 5 onward with a concomitant increase in FCR as compared to feeding a SD. Similar to this study, a previous study documented a decline in EW, EP rate, and FI in hens fed a CD [29]. Notably, these reductions began in weeks 2, 3, and 4 for each measure, respectively, and affected both young and old hens [29]. Moreover, another study reported that hens fed a CD consumed less feed and had a lower EP rate than hens on a control diet [7]. It is assumed that energy consumption plays a key role in controlling FI [30]. When a CD with a high ratio of energy to protein is fed to hens, more fat is accumulated in the liver and body tissues. Body lipid reserves secret leptin, a peptide hormone, which reduces FI [29, 31]. Excessive lipid accumulation in the liver predisposes laying hens to FLHS. This condition triggers inflammation and discomfort, leading to a decline in EP and a rise in FCR. FLHS in laying hens can be developed through a combination of factors, including impaired lipid metabolism, hormonal imbalances, oxidative stress, disruption of the metabolism and utilization of nutrients, impaired vitamin D metabolism, inflammation, and reduced blood flow to the reproductive organs [4, 7, 32, 33]. In the current study, the CD + Mix 2 compensated for the damage caused by the CD to a large extent, and improved FI, EP and FCR of laying hens. The improvement is believed to be due to Mix 2's ability to inhibit fat production, enhance fat burning, reduce oxidative stress, and suppress liver inflammation [34]. Sumac, lemon, black pepper and chicory, all components of Mix 2, possess various bioactive compounds with antioxidant, anti-inflammatory, and lipid-lowering properties [11-14, 22]. Two theories have been proposed in the pathogenesis of FLHS. The first theory proposes that FLHS starts with the excessive deposition of triglyceride in the liver, which disrupt the homeostasis of lipid metabolism. The second theory proposes oxidative stress in FLHS induces excessive reactive oxygen species in the liver, which leads to the destruction of hepatic connective tissues, called reticulin [35]. The antioxidant and

antihyperlipidemic properties of secondary metabolites in the Mix 2 could have helped improving the liver function and overall health. It is worth noting that the simultaneous use of these herbal additives could have a synergistic effect compared to using them alone. Limited information is available about the role of medicinal plants in prevention of FLHS in laying hens. A previous study found that adding a mixture of chicory, turmeric, artichoke, and milk thistle powder to a high energy/low protein diet did not affect EP or FCR in laying hens. However, artichoke and thistle powder reduced FI while turmeric powder increased EW compared to the control group [36]. Another study observed that broiler chickens fed diets containing chicory root powder showed significant improvement in growth performance, likely due to increased food digestion and absorption caused by changes in jejunum histomorphometry [37]. Supplementation with 0.5% sumac in feed was found to slightly increase EW in another study, although FI, FCR, and EP were not significantly affected [38]. Research has shown that adding lemon essential oil to quail feed improved FCR and FI, possibly due to the active compounds, antioxidant, and antimicrobial properties of the oil [39]. A recent study demonstrated that incorporating garlic into laying hen diets could increase EW [40]. Additionally, another study reported increased EP, EW, and FI in groups treated with inulin [41]. Inconsistencies observed across these studies may be attributed to factors such as variations in dosage, limited absorption of the additives, and potential interactions between different herbal plants.

Laying hens fed on CD, with or without herbal additives, had higher blood AST compared to those on SD in the middle (second sampling) and the end (third sampling) of the experiment. In this situation, supplementing CD with the Mix 2 restored the response. AST is a liver enzyme released into the bloodstream when liver cells are damaged. Hence, a high level of AST in the blood is an indicator of liver damage or inflammation [42]. Studies have shown that feeding hens a high-energy, low-protein diet can increase AST levels [29, 43]. Although there is limited information on the effect of medicinal plants on FLHS in laying hens, the antioxidant and anti-inflammatory effects of these herbal additives can assist in improving liver health and decreasing AST concentration [11, 13, 44]. For example, one study found that feeding quails with lemon essential oil reduced AST levels, possibly due to the oil's protective effects on cells [39]. However, other studies have shown that sumac supplementation does not significantly affect

AST levels [45, 46]. Similarly, a recent study found that including chicory, turmeric, artichoke, and milk thistle powder in a high-energy, low-protein diet did not significantly impact AST concentration in laying hens [36]. In contrast, another study showed that adding a mixture containing milk thistle to the diet of hens with fatty liver reduced blood AST concentration [47]. These differing results may be due to factors such as the dosage of the herbal additives, interactions between different herbs, or the quality of the herbs used. All herbal mixtures used in the SD increased blood TP concentration compared to the control (SD without additives). Such an increase could be linked to improved utilization of dietary protein due to a better gut health and antioxidant status. It has been reported that ginger supplementation at a rate of 5 g/kg of diet increased TP concentrations in the serum of broilers, likely due to enhanced antioxidant status [48]. Additionally, Oleforuh-Okoleh et al. [49] showed that adding garlic to broiler diets increased blood TP compared to the control group. The only treatment in CD group with TP concentration similar to SD groups was CD + Mix 2. A positive correlation between dietary protein and plasma protein concentration has been established [50]. It is possible that low protein levels in CD groups may be caused by a decrease in protein utilization, as well as an increase in protein turnover. Studies have shown that blood TP concentration increased with the addition of lemon essential oil in quail's diets and that chicory, turmeric, artichoke, and thistle powder did not affect blood TP concentration in laying hens with fatty liver [36, 39]. Additionally, Mosayyeb Zadeh et al. [46] found that using 0, 0.25, and 0.5% sumac in the diet of laying hens had no effect on TP concentration. These findings differ from the present study. The potential synergistic effects among herbs in Mix 2 may have resulted in a more pronounced outcome compared to the use of single herbs. Research has shown that the synergistic effect is based on physicochemical interactions among the bioactive compounds in herbs [51].

Increased blood UA concentration in CD groups was not compensated by herbal additives. The lowest concentration of UA was observed in SD + Mix 2 and SD + Mix 3. UA is a byproduct of protein catabolism, which is eliminated from the body through urinary excretion. Research suggests that fatty liver increases circulatory levels of UA, and UA may be considered as an independent predictor of the risk for fatty liver [52]. In agreement with our results, a study found that dietary inclusion of chicory, turmeric, artichoke, and

thistle powder to a high-energy, low-protein diet did not significantly change UA concentration in laying hens [36]. Mosayyeb Zadeh et al. [46] indicated that dietary levels of 0.25, and 0.5% sumac did not influence the concentration of UA in laying hens.

Blood TG concentration of laying hens was increased in CD groups in the second samplings. However, in the third sampling, CD + Mix 2 attenuated this increase compared to other CD treatments. Excess fat could be accumulated as TG in the liver and body tissues and streamed in the blood due to inability of the liver to deal with the overloaded of lipids. Studies suggest that sumac and its polyphenols may lower TG by hindering fat digestion and absorption [46, 53]. Similarly, an herbal mix containing lemon was reported to reduce TG in overweight rats [54]. The mechanism might involve lemon's vitamin C and flavonoid content acting as antioxidants [44, 55]. The concentration of TC was increased under the influence of CD in the second and third samplings, and herbal additives were unable to restore these effects. It was suggested that TC and TG contents were increased in FLHS [6, 43]. Inability of herbal additives in reducing blood TC of birds in CD groups in the current study remained unexplained.

Factors that are thought to be involved in the occurrence of FLHS include duration of feeding the challenge diet, the composition of herbal mixes, and interactions among herbal additive. The lowest concentration of TC was observed in the SD + Mix 3. Garlic and shallot belong to the Allium specie, and several researchers have suggested that they had hypocholesterolemic effects in laying hens and quails [16, 56, 57]. Akbarian et al. [58] showed a decrease in blood TC of laying hens treated with ginger root powder (0.25, 0.5, and 0.75) for 8 weeks. One possible mechanism was the modulation of bile acids [48]. Moreover, artichoke decreased blood TC through increased biliary clearance [59].

In the present study, CD increased blood LDL concentration of laying hens compared to SD in the second sampling, and no other changes were seen in LDL concentration in laying hens fed by SD or CD with herbal additives. High blood LDL level is a major risk factor for heart disease [60]. In general, there was no decrease in blood HDL in CD groups with or without herbal additives compared to the SD in the present study. HDL is a type of lipoprotein that helps transport cholesterol from peripheral tissues to the liver for elimination from the body. It is often referred to as "good cholesterol" due to its positive impact on reducing

the risk of cardiovascular diseases [61]. The balance between LDL and HDL levels in the blood reflects the state of lipid metabolism in the body [62]. A study found an increase in LDL and a decrease in HDL in a high-energy, low-protein group compared to the control group [35]. Their finding was not consistent with our result. Fatty liver in laying hens have a negative effect on HDL levels, but it does not always do so. There are a number of factors that can influence the effect of fatty liver on HDL levels, including severity and duration of fatty liver, diet and nutrition, and genetic factors. In the present study, layers fed SD + Mix 3 showed the highest level of HDL in the second and third sampling compared to the other treatments. Studies have shown increased HDL cholesterol in laying quails when fed diets containing 1, 2, and 4% garlic powder [63]. Saki et al. [64] showed that serum HDL levels in laying hens were significantly increased when fed diets containing 8 or 12 g kg-1 herb mixture. Moreover, an increase in serum HDL cholesterol was reported in all treated groups with Ginger rhizome oil in Japanese quails, compared to the control group [65].

The concentration of Hb in different sampling times did not follow a specific trend, but in general, there was an elevation of Hb in laying hens fed CD with and without herbal additives compared to the SD + Mix 2 and SD + Mix 3. A previous study showed that a high-fat diet increased the amount of Hb in the blood of mice compared to the control diet with herbal additives [54]. It is important to keep in mind that the impact of fatty liver on Hb concentration in laying hens can be quite complex and may depend on various factors, including the severity and underlying cause of the fatty liver, as well as the metabolic pathways involved in erythropoietin production and red blood cell destruction. Another study indicated that patients with fatty liver had high circulating Hb level, which was related to the degree of hepatic steatosis, and this Hb may act as an antioxidant to neutralize the adverse effects of this disease [66].

In this study, the difference between the numbers of heterophiles in laying hens fed CD with and without additives was not significant, but the SD + Mix 1 showed the highest count of heterophiles in the second and third sampling. The number of lymphocytes were increased in CD groups and the lowest number was observed in diets SD + Mix 2 and SD + Mix 3. According to a previous study, a low count of white blood cells may indicate a disease-free condition or suppressed production in bone marrow [67]. It is presumed

that medicinal plants in these two mixes have anti-inflammatory, antimicrobial, and immune system-stimulating properties. It is presumed that alleviating the fatty liver symptoms could have reduced the number of lymphocytes. By adding 25 and 50 mg/kg garlic to the diet of broilers, a study found no significant effect on lymphocyte counts. However, the higher inclusion level (50mg/kg) significantly reduced heterophiles relative to the control [68]. In contrast to our results, another study reported that garlic increased heterophiles, lymphocytes, and the heterophile to lymphocyte ratio, and attributed these responses to a stimulatory effect of garlic on the immune system [69]. In our study, CD alone resulted in more lymphocytes compared to CD + Mix 2. It shows that the combination of herbal plants in this herbal mix may have attenuated CD stress. It is worth noting that other factors, such as age, breed, and environmental conditions, could also affect heterophile levels. Therefore, it is difficult to predict how fatty liver will affect heterophile levels in hens. A study reported that feeding a diet containing lemon and garlic decreased the number of white blood cells compared to the control diet with high fat [54].

Lower liver histopathology scores in hens fed the CD with Mix 2 compared to those without were observed in this study, suggesting a potential role for the herbs in Mix 2 against FLHS development. The precise mechanisms by which Mix 2 exerts this protective effect remain unclear. However, the individual components within the blend possess properties potentially contributing to FLHS prevention. For example, lemon and black pepper, known for their antioxidant and anti-inflammatory effects [12, 13, 70], could potentially combat oxidative stress and inflammation in the liver, key factors in FLHS pathogenesis. Sumac, shown to regulate lipid metabolism [11], could potentially aid in preventing excessive fat accumulation in the liver. Moreover, studies suggest that chicory in the diet may improve health and reduce liver histology changes [71]. This reduction might be due to isoflavones, polyphenols, and antioxidants present in chicory, which may mitigate liver injury [71]. The existing data on the impact of medicinal plants on liver pathology is mixed. While some research reported no significant effect of herbal essential oil mixtures on liver parameters [36, 72], others demonstrated the effectiveness of specific herbal mixtures in reducing liver fat accumulation and inflammation in laying hens [47]. These findings highlight the variability in responses. Trott et al. [4] noted varying amounts of hepatocellular vacuolization in FLHS chickens, indicating not all

have severe fat buildup. Additionally, Song et al. [28] reported that the severity of liver lesions can vary depending on the hens' age and diet composition. Further research is necessary to explore the individual and synergistic effects of these herbs on liver health and FLHS development.

## Conclusion

The present experiment showed that hens fed the CD had lower FI, EW, and EP rate, as well as higher blood levels of AST (indicating liver damage) and UA (indicating protein catabolism). Their livers also showed a higher histopathology score consistent with FLHS. Mix 2, containing sumac, lemon, black pepper, and chicory, improved performance (FI, EW, and EP rate) and AST content in hens fed the CD. Mix 2 also reduced liver histopathology scores compared to hens fed the CD alone. The effects of other medicinal plant mixtures were less consistent. Some improved certain parameters but not others, while others had no significant effects. Overall, this study suggests that some medicinal plant mixtures, particularly Mix 2, have the potential to mitigate the negative effects of FLHS in laying hens.

## List of abbreviations

**AST**: Aspartate aminotransferase

**CD:** Challenge diet

**EP**: Egg production

**EW**: Egg weight

**FCR**: Feed conversion ratio

**FI**: Feed intake

**FLHS**: Fatty liver hemorrhage syndrome

**Hb**: Hemoglobin

**HDL**: High-density cholesterol

**LDL**: Low-density cholesterol

**SD:** Standard diet

**TC**: Total cholesterol

**TG**: Triglycerides

**UA**: Uric acid

## Declarations

### Ethics approval and consent to participate

All experimental protocols adhered to the guidelines, which were approved by, the Animal Ethics Committee of the Razi University, Kermanshah, Iran, and were in accordance with the EU standards for the protection of animals and/or feed legislation.

### Consent for publication

Not applicable.

### Availability of data and materials

All data generated or analysed during this study are included in this published article [and its supplementary information files].

### Competing interests

The authors declare that they have no competing interests.

### Funding

No funding was received to assist with the preparation of this manuscript.

### Authors' contributions

Mohammadreza Khodaei conducted the experiment, gathered the data, created the result tables, and authored the initial version of the manuscript. Mehran Torki was responsible for the research design and

oversight, as well as for revising and submitting the draft manuscript. Fariborz Khajali conducted the statistical analysis of the experimental data and revised the manuscript. Iraj Karimi conducted a histopathological examination of the liver. All authors reviewed and endorsed the final manuscript.

## Acknowledgements

The authors appreciate Razi University for financially supporting the current study.

## References

1. Aydin, R. 2005. Type of fatty acids, lipoprotein secretion from liver and fatty liver syndrome in laying hens. Int J Poult Sci. 2005;4:917-919.

2. Butler EJ. Lipid metabolism in the fowl under normal and abnormal circumstances. Proc Nutr Soc. 1975;34:29-34. https://doi.org/10.1079/PNS19750007

3. Butler EJ. Fatty liver diseases in the domestic fowl—A review. Avian Pathol. 1976;5:1-4. https://doi.org/10.1080/03079457608418164

4. Trott KA, Giannitti F, Rimoldi G, Hill A, Woods L, Barr B, et al. Fatty liver hemorrhagic syndrome in the backyard chicken: a retrospective histopathologic case series. Vet Pathol. 2014;51:787-95. https://doi.org/10.1177/03009858135035

5. Shini A, Shini S, Bryden WL. Fatty liver haemorrhagic syndrome occurrence in laying hens: impact of production system. Avian Pathol. 2019;48:25-34. https://doi.org/10.1080/03079457.2018.1538550

6. Choi YI, Ahn HJ, Lee BK, Oh ST, An BK, Kang CW. Nutritional and hormonal induction of fatty liver syndrome and effects of dietary lipotropic factors in egg-type male chicks. Asian-Australas J Anim Sci. 2012;25:1145. https://doi.org/10.5713/ajas.2011.11418

7. Jiang S, Cheng HW, Cui LY, Zhou ZL, Hou JF. Changes of blood parameters associated with bone remodeling following experimentally induced fatty liver disorder in laying hens. Poult Sci. 2013;92:1443-53. https://doi.org/10.3382/ps.2012-02800

8. Squires EJ, Leeson S. Aetiology of fatty liver syndrome in laying hens. Br Vet J. 1988;144:602-609. https://doi.org/10.1016/0007-1935(88)90031-0

9. Pearson AW, Butler EJ, Curtis RF, Fenwick GR, Hobson-Frohock A, Land DG, et al. Effects of rapeseed meal on laying hens (Gallus domesticus) in relation to fatty liver-haemorrhagic syndrome and egg taint. Res Vet Sci. 1978;25:307-13. https://doi.org/10.1016/S0034-5288(18)32945-X

10. Couch JR. Fatty livers in laying hens—a condition which may occur as a result of increased strain. Feedstuffs. 1956;28(47):46.

11. Shariatmadari F, Shariatmadari R. Sumac (Rhus coriaria) supplementation in poultry diet. World's Poult Sci. J 2020;76:358-64. https://doi.org/10.1080/00439339.2020.1745724

12. Manners GD. Citrus limonoids: analysis, bioactivity, and biomedical prospects. J Agric Food Chem. 2007;55:8285-94. https://doi.org/10.1021/jf071797h

13. Zuzanna Bober Z, Agnieszka Stępień A, David Aebisher D, Łukasz Ożog Ł, Dorota Bartusik-Aebisher D. Medicinal benefits from the use of Black pepper, Curcuma and Ginger. Eur J Clin Exp Med. 2018;133-45. 10.15584/ejcem.2018.2.9

14. Nandagopal S, Kumari BR. Phytochemical and antibacterial studies of Chicory (Cichorium intybus L.)-A multipurpose medicinal plant. Adv Biol Res. 2007;1:17-21.

15. Khazaei R, Seidavi A, Bouyeh M. A review on the mechanisms of the effect of silymarin in milk thistle (Silybum marianum) on some laboratory animals. Vet Med Sci. 2022;8:289-301. https://doi.org/10.1002/vms3.641

16. Kothari D, Lee WD, Niu KM, Kim SK. The genus Allium as poultry feed additive: A review. Animals. 2019;9:1032. https://doi.org/10.3390/ani9121032

17. Abadjieva D, Grigorova S, Mladenova V, Shimkus A, Kistanova E. Effect of artichoke (Cynara scolimus L.) on the egg productivity and biochemical parameters in laying hens. Bulg J Agric Sci. 2020; 26: 1280-5.

18. Chung MY, Park HJ, Manautou JE, Koo SI, Bruno RS. Green tea extract protects against nonalcoholic steatohepatitis in ob/ob mice by decreasing oxidative and nitrative stress responses induced by proinflammatory enzymes. J Nutr Biochem. 2012;23:361-7. https://doi.org/10.1016/j.jnutbio.2011.01.001

19. Kermanshahi H, Riasi A. Effect of turmeric rhizome powder (Curcuma longa) and soluble NSP degrading enzyme on some blood parameters of laying hens. Int J Poult Sci. 2006;5:494-8.

20. Sharma UR, Prakash T, Surendra V, Roopakarki N, Rao NR, Divakar G. Hepatoprotective activity of Fumaria officinalis against CCl4-induced liver damage in rats. Pharmacology. 2012;3:9-14.

21. Khan RU, Naz S, Nikousefat Z, Tufarelli VI, Javdani M, Qureshi MS, et al. Potential applications of ginger (Zingiber officinale) in poultry diets. World's Poult Sci J. 2012;68:245-52. https://doi.org/10.1017/S004393391200030X

22. Li XL, He WL, Wang ZB, Xu TS. Effects of Chinese herbal mixture on performance, egg quality and blood biochemical parameters of laying hens. J Anim Physiol Anim Nutr. 2016;100:1041-9. https://doi.org/10.1111/jpn.12473

23. Naghneh NS, Rafiei F, Shahbazi E, Gheisari MM. Variation of volatile compounds in celery through different vegetative stages. Plant Physiol. 2023;28:476-80. https://doi.org/10.1007/s40502-023-00744-6

24. Cherian G, Hayat Z. Long-term effects of feeding flaxseeds on hepatic lipid characteristics and histopathology of laying hens. Poult Sci. 2009;88:2555-61. https://doi.org/10.3382/ps.2009-00425

25. SAS (Statistical Analyses System). 2015. SAS user's guide. Version 9.4. Cary, NC: SAS Inst.

26. Karmakar R, Pahari P, Mal D. Phthalides and Phthalans: Synthetic methodologies and their applications in the total synthesis. Chem Rev. 2014;114: 6213–84. https://doi.org/10.1021/cr400524q

27. Moses T, Pollier J, Thevelein J M, Goossens A. Bioengineering of plant (tri) terpenoids: From metabolic engineering of plants to synthetic biology in vivo and in vitro. New Phytologist. 2013;200: 27–43. https://doi.org/10.1111/nph.12325

28. Song Y, Ruan J, Luo J, Wang T, Yang F, Cao H, et al. Abnormal histopathology, fat percent and hepatic apolipoprotein AI and apolipoprotein B100 mRNA expression in fatty liver hemorrhagic syndrome and their improvement by soybean lecithin. Poult Sci. 2017;96:3559-63. https://doi.org/10.3382/ps/pex163

29. Rozenboim I, Mahato J, Cohen NA, Tirosh O. Low protein and high-energy diet: a possible natural cause of fatty liver hemorrhagic syndrome in caged White Leghorn laying hens. Poult Sci. 2016;95:612-21. https://doi.org/10.3382/ps/pev367

30. McNab JM, Boorman KN. Poultry feedstuffs: supply, composition and nutritive value. CABI publishing. 2002. https://doi.org/10.1079/9780851994642.0000

31. Richards MP. Genetic regulation of feed intake and energy balance in poultry. Poult Sci. 2003;82:907-16. https://doi.org/10.1093/ps/82.6.907

32. Shini S, Bryden WL. Occurrence and control of fatty liver haemorrhagic syndrome (FLHS) in caged hens. A report for the Australian Egg Corporation Limited AECL Publication No uq-105a AECL Project No UQ-105. 2009;1448-316.

33. Lee BK, Kim JS, Ahn HJ, Hwang JH, Kim JM, Lee HT, et al. Changes in hepatic lipid parameters and hepatic messenger ribonucleic acid expression following estradiol administration in laying hens (Gallus domesticus). Poult Sci. 2010;89:2660-7. https://doi.org/10.3382/ps.2010-00686

34. Panyod S, Sheen LY. Beneficial effects of Chinese herbs in the treatment of fatty liver diseases. J Tradit Complement Med. 2020;10:260-7. https://doi.org/10.1016/j.jtcme.2020.02.008

35. Miao YF, Gao XN, Xu DN, Li MC, Gao ZS, Tang ZH, et al. Protective effect of the new prepared Atractylodes macrocephala Koidz polysaccharide on fatty liver hemorrhagic syndrome in laying hens. Poult Sci. 2021;100:938-48. https://doi.org/10.1016/j.psj.2020.11.036

36. Assi Husain H, Sadeghi A, Karimi A. Effects of chicory, turmeric, artichoke and sage powder in high energy and low protein diets on yield, egg quality and fatty liver status in laying hens. Anim Sci. 2023;36:19-38. 10.22092/ASJ.2022.358220.2215

37. Izadi H, Arshami J, Golian A, Raji MR. Effects of chicory root powder on growth performance and histomorphometry of jejunum in broiler chicks. Vet Res Forum. 2013;4:169-174.

38.Arpášová H, Kačániová M, Gálik B, Čuboň J, Mellen M. The effect of oregano essential oil and Rhus coriaria L. on selected performance parameters of laying hens. Scientific Papers Anim Sci Biotech. 2014;47:12-12.

39. Alagawany M, El-Saadony MT, Elnesr SS, Farahat M, Attia G, Madkour M, et al. Use of lemongrass essential oil as a feed additive in quail's nutrition: its effect on growth, carcass, blood biochemistry,

antioxidant and immunological indices, digestive enzymes and intestinal microbiota. Poult Sci. 2021;100:101172. https://doi.org/10.1016/j.psj.2021.101172

40.Omer HA, Ahmed SM, Abdel-Magid SS, El-Mallah GM, Bakr AA, Abdel Fattah MM. Nutritional impact of inclusion of garlic (Allium sativum) and/or onion (Allium cepa L.) powder in laying hens' diets on their performance, egg quality, and some blood constituents. Bull Natl Res Cent. 2019;43:1-9. https://doi.org/10.1186/s42269-019-0061-6

41. Park SO, Park BS. Effect of feeding inulin oligosaccharides on cecum bacteria, egg quality and egg production in laying hens. Afric J Biotech. 2012;11:9516-21. https://doi.org/10.5897/AJB12.5250

42. Yousefi M, Shivazad M, Sohrabi-Haghdoost I. Effect of dietary factors on induction of fatty liver-hemorrhagic syndrome and its diagnosis methods with use of serum and liver parameters in laying hens. Int J Poult Sci. 2005;4:468-72.

43. Zhang J, Chen D, Yu B. Effect of different dietary energy sources on induction of fatty liver-hemorrhagic syndrome in laying hens. Int J Poult Sci. 2008;7:1232-6.

44. González-Molina E, Domínguez-Perles R, Moreno DA, García-Viguera C. Natural bioactive compounds of Citrus limon for food and health. J Pharm Biomed Anal. 2010;51:327-45. https://doi.org/10.1016/j.jpba.2009.07.027

45. Gumus H, Oguz MN, Bugdayci KE, Oguz FK. Effects of sumac and turmeric as feed additives on performance, egg quality traits, and blood parameters of laying hens. Rev Bras de Zootecn. 2018;47. https://doi.org/10.1590/rbz4720170114

46. Mosayyeb Zadeh A, Mirghelenj SA, Shakouri Alishah H, Hasanlou P. Effects of sumac fruit powder (Rhus coriaria L.) supplementation on productive performance, egg quality traits and serum biochemical

parameters in old laying hens. Ital J Anim Sci. 2021;20:1348-56. https://doi.org/10.1080/1828051X.2021.1965922

47. Zhu Y, Zhang X, Du P, Wang Z, Luo P, Huang Y, et al. Dietary herbaceous mixture supplementation reduced hepatic lipid deposition and improved hepatic health status in post-peak laying hens. Poult Sci. 2022;101:101870. https://doi.org/10.1016/j.psj.2022.101870

48. Zhang GF, Yang ZB, Wang Y, Yang WR, Jiang SZ, Gai GS. Effects of ginger root (Zingiber officinale) processed to different particle sizes on growth performance, antioxidant status, and serum metabolites of broiler chickens. Poult Sci. 2009;88:2159-66. https://doi.org/10.3382/ps.2009-00165

49. Oleforuh-Okoleh VU, Ndofor-Foleng HM, Olorunleke SO, Uguru JO. Evaluation of growth performance, haematological and serum biochemical response of broiler chickens to aqueous extract of ginger and garlic. J Agric Sci. 2015;7:167. https://doi.org/10.5539/jas.v7n4p167

50. Bush BM. Interpretation of laboratory results for small animal clinicians. Blackwell Scientific Publications Ltd. 1991;225-9.

51. Kiyohara H, Matsumoto T, Yamada H. Combination effects of herbs in a multi-herbal formula: expression of Juzen-taiho-to's immuno-modulatory activity on the intestinal immune system. J Evid Based Complementary Altern Med. 2004;1:83-91. https://doi.org/10.1093/ecam/neh004

52. Oral A, Sahin T, Turker F, Kocak E. Relationship between serum uric acid levels and nonalcoholic fatty liver disease in non-obese patients. Med. 2019;55:600. https://doi.org/10.3390/medicina55090600

53. Shishikura Y, Khokhar S, Murray BS. Effects of tea polyphenols on emulsification of olive oil in a small intestine model system. J Agric Food Chem. 2006;54:1906-13. https://doi.org/10.1021/jf051988p

54. Bassuony NI, Hafez LM, Ibrahim AF, El-Sebeay AS, Omer HA. Effect of using natural bioactive mixture composed of lemon, onion and garlic juices on weight and lipid profiles for overweight rats. Eur J Appl Sci. 2021;13:09-17.

55. Prabuseenivasan S, Jayakumar M, Ignacimuthu S. In vitro antibacterial activity of some plant essential oils. BMC Complement Altern Med. 2006;6:1-8. https://doi.org/10.1186/1472-6882-6-39

56. Chowdhury SR, Chowdhury SD, Smith TK. Effects of dietary garlic on cholesterol metabolism in laying hens. Poult Sci. 2002;81:1856-62. https://doi.org/10.1093/ps/81.12.1856

57. Mahmoud KZ, Gharaibeh SM, Zakaria HA, Qatramiz AM. Garlic (Allium sativum) supplementation: Influence on egg production, quality, and yolk cholesterol level in layer hens. Asian-Australas J Anim Sci. 2010;23:1503-9. https://doi.org/10.5713/ajas.2010.10124

58. Akbarian A, Golian A, Sheikh Ahmadi A, Moravej H. Effects of ginger root (Zingiber officinale) on egg yolk cholesterol, antioxidant status and performance of laying hens. J Appl Anim Res. 2011;39:19-21. https://doi.org/10.1080/09712119.2011.558612

59. Qiang Z, Lee SO, Ye Z, Wu X, Hendrich S. Artichoke extract lowered plasma cholesterol and increased fecal bile acids in Golden Syrian hamsters. Phytother Res. 2012;26:1048-52. https://doi.org/10.1002/ptr.3698

60. Ridker PM. LDL cholesterol: controversies and future therapeutic directions. Lancet. 2014;384:607-17. https://doi.org/10.1016/S0140-6736(14)61009-6

61. Hermansen K, Dinesen B, Hoie LH, Morgenstern E, Gruenwald J. Effects of soy and other natural products on LDL: HDL ratio and other lipid parameters: a literature review. Adv Ther. 2003;20:50-78. https://doi.org/10.1007/BF02850119

62. Zhang X, Han Z, Zhong H, Yin Q, Xiao J, Wang F, et al. Regulation of triglyceride synthesis by estradiol in the livers of hybrid tilapia (Oreochromis niloticus♀× O. aureus♂). Comparative Biochemistry and Physiology Part B: Biochem Mol Biol. 2019;238:110335. https://doi.org/10.1016/j.cbpb.2019.110335

63. Canogullari S, Baylan M, Erdogan Z, Duzguner V, Kucukgul A. The effects of dietary garlic powder on performance, egg yolk and serum cholesterol concentrations in laying quails. Czech J Anim Sci. 2010;55:286-93. https://doi.org/10.17221/126/2009-CJAS

64. Saki AA, Aliarabi H, Siyar SA, Salari J, Hashemi M. Effect of a phytogenic feed additive on performance, ovarian morphology, serum lipid parameters and egg sensory quality in laying hen. Vet Res Forum. 2014;5:287–293.

65. Herve T, Raphaël KJ, Ferdinand N, Victor Herman N, Willy Marvel NM, Cyril D'Alex T, et al. Effects of ginger (Zingiber officinale, Roscoe) essential oil on growth and laying performances, serum metabolites, and egg yolk antioxidant and cholesterol status in laying Japanese quail. J Vet Med. 2019;2019. https://doi.org/10.1155/2019/7857504

66. Juárez-Hernández E, Chávez-Tapia NC, Brizuela-Alcántara DC, Uribe M, Ramos-Ostos MH, Nuño-Lámbarri N. Association between serum hemoglobin levels and non alcoholic fatty liver disease in a mexican population. Ann Hepatol. 2018;17:577-84. https://doi.org/10.5604/01.3001.0012.0920

67. Medugu CI, Kwari ID, Igwebuike J, Nkama I, Mohammed ID, Hamaker B. Carcass and blood components of broiler chickens fed sorghum or millet as replacement for maize in the semi arid zone of Nigeria. Agric Biol J North Am. 2010;1:326-9.

68. El-katcha MI, Soltan MA, Sharaf MM, Hasen A. Growth performance, immune response, blood serum parameters, nutrient digestibility and carcass traits of broiler chicken as affected by dietary supplementation of garlic extract (allicin). Alex J Vet Sci. 2016 15;49. https://doi.org/10.5455/ajvs.219261

69. Eid KM, Iraqi MM. Effect of garlic powder on growth performance and immune response for newcastle and avian influenza virus diseases in broiler of chickens. Anim Biotechnol. 2014;1:7-13.

70. Li Y, Yang M, Lin H, Yan W, Deng G, Ye H, et al. Limonin alleviates non-alcoholic fatty liver disease by reducing lipid accumulation, suppressing inflammation and oxidative stress. Front Pharmacol. 2022;12:801730.

71. Jamzadfard M, Ebrahimi H. Herbal Medicine in the Treatment of Fatty Liver Disease: Evaluation of the therapeutic Effects of some herbs that constitute Magic Liver herbal distillate.

72. Mousavi A, Mahdavi AH, Riasi A, Soltani-Ghombavani MJ. Synergetic effects of essential oils mixture improved egg quality traits, oxidative stability and liver health indices in laying hens fed fish oil. Anim Feed Sci Technol. 2017;234:162-72.

73. Amiri HR, Mohammadi M, Sadatmand S, Taheri E. Study the chemical composition of essential oil of ginger (zingiber officinale) and antioxidant and cell toxicity. J Med Plants. 2016 ;15:89-98.

74. Majdi B, Mehrnia MA, Barzegar H, Alizadeh Behbahani B. Determination of the structure, chemical composition, antioxidant activity and the cytotoxic effect of Turmeric essential oil. Iran Food Sci Technol Res J. 2021;17(2):261-71. https://doi.org/10.22067/ifstrj.2020.39272

75. Khoshtinat K, Barzegar M, Sahari MA, Hamidi Z. Encapsulation of Iranian Garlic Oil with β-cyclodextrin: Optimization and its Characterization. J Agric Sci Technol. 2017;19: 97-111.

76. Morshed S, Hossain MD, Ahmad M, Junayed M. Physicochemical characteristics of essential oil of black pepper (Piper nigrum) cultivated in Chittagong, Bangladesh. J. Food Qual Hazards Control. 2017;4(3):66-9.

**Figure 1**. Representative liver histopathology under H&E staining at increasing grades of fatty change. (A) Normal liver shows uniform architecture with centrally located nuclei (score 1). (B) Mild fatty change (score 2) exhibits minimal fatty vacuoles (clear spaces) within hepatocytes. (C) Moderate fatty change (score 3) demonstrates increased cytoplasmic vacuolization. (D) Severe fatty change (score 4) displays extensive fatty infiltration obscuring most cytoplasm. Arrowhead highlights an acentric nucleus, a characteristic finding in steatosis.

**Figure 2**. The Effect of different combinations of herbal additives with standard and challenge diet on the liver histopathology score of laying hens. SD, standard diet; CD, challenge diet; Mix 1= turmeric, fumitory, green tea and milk thistle powder; Mix 2= lemon, black pepper, sumac and chicory powder; Mix 3= garlic, artichoke, ginger and shallot powder. Different letters indicate significant difference among different treatments at the 0.05 level

Click here to access/download;Figure;Figure 1.docx

**Figure 1.**

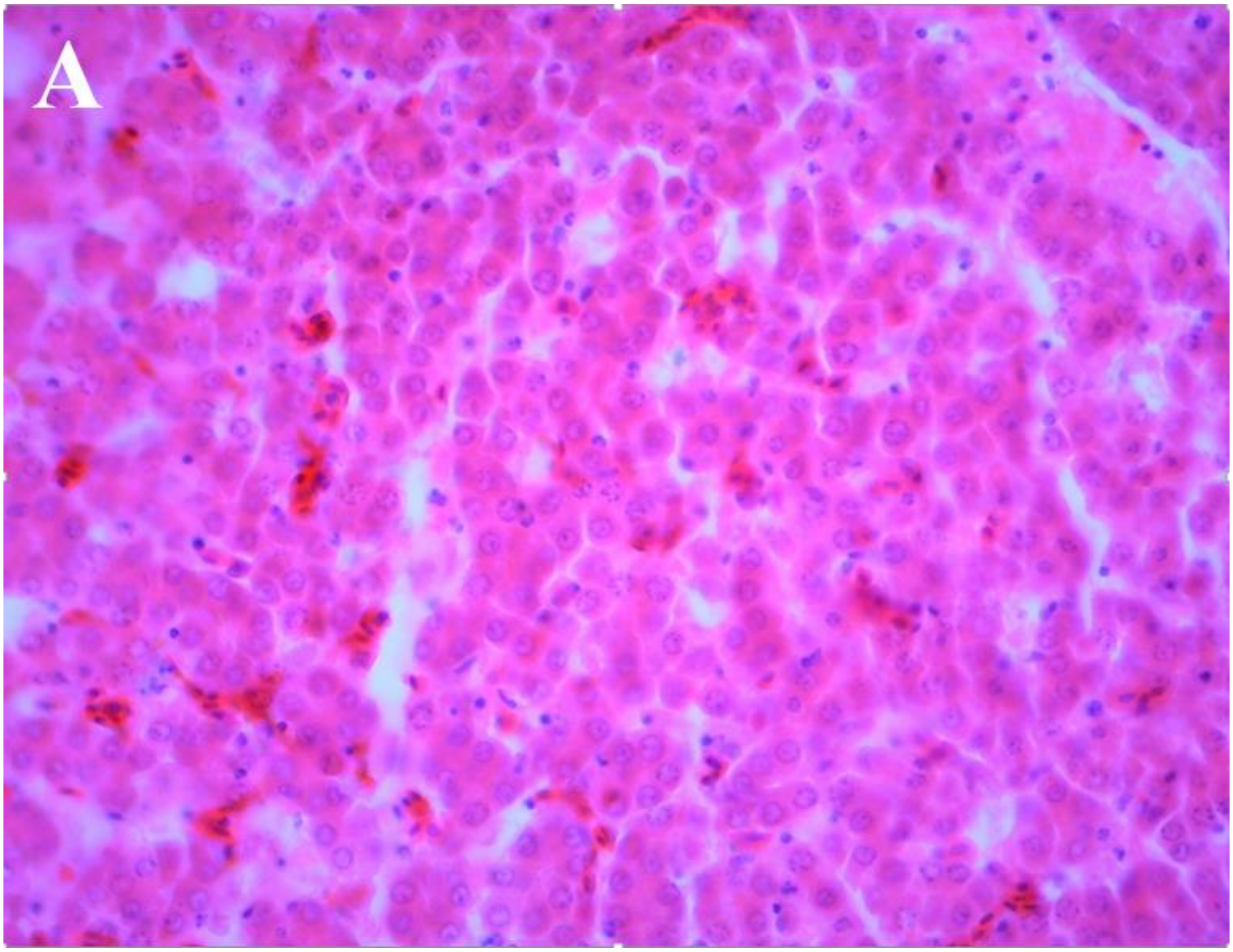

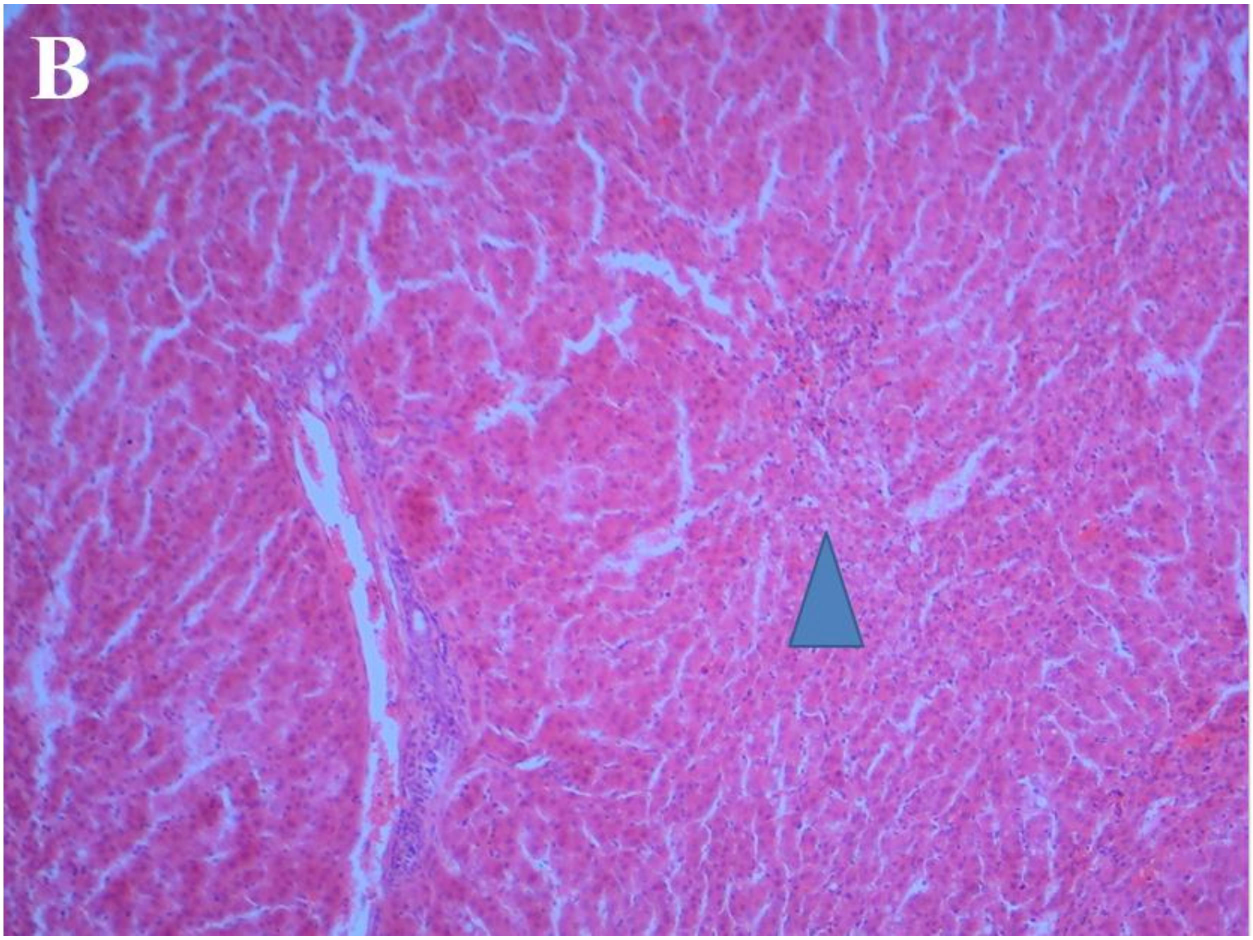
B

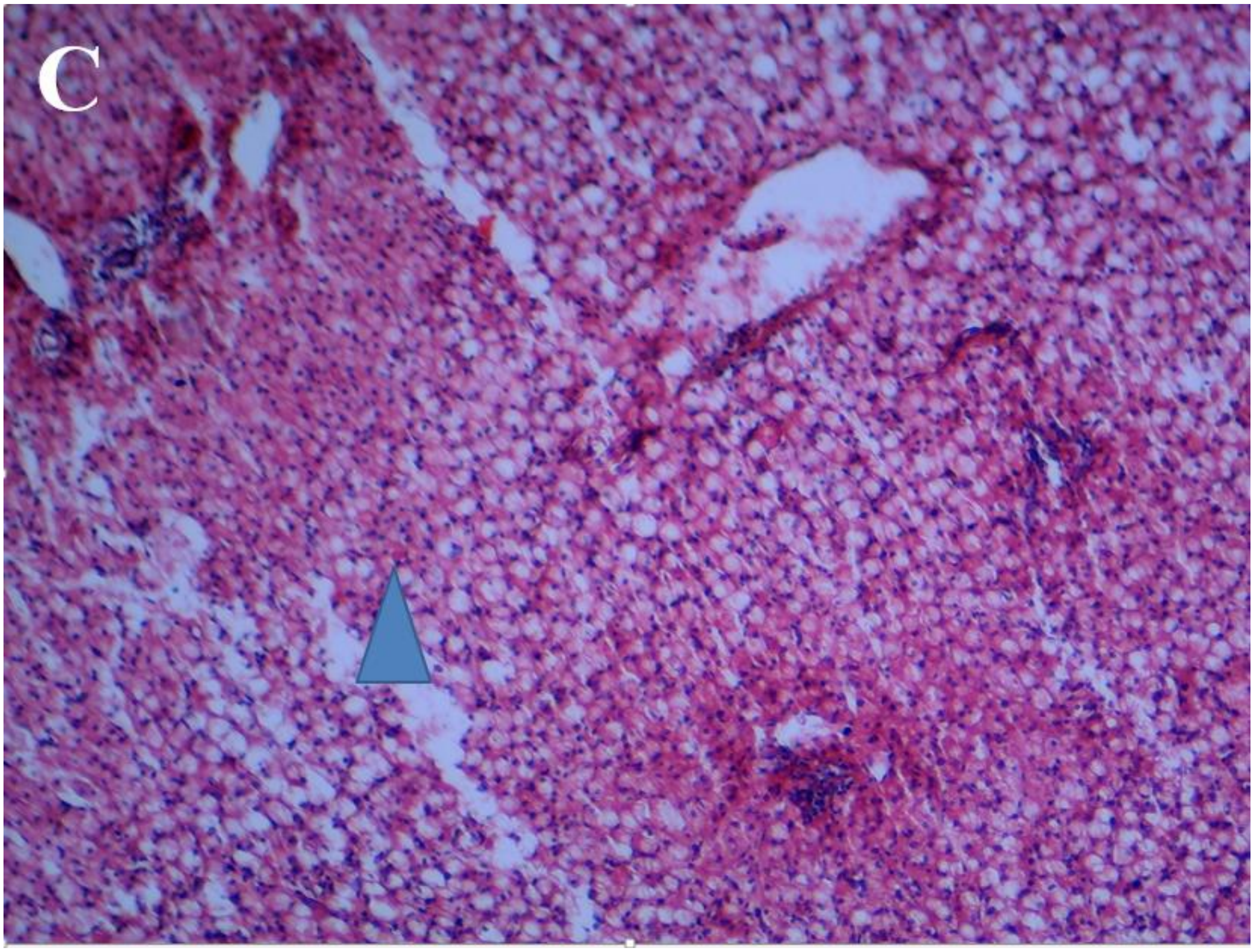
C

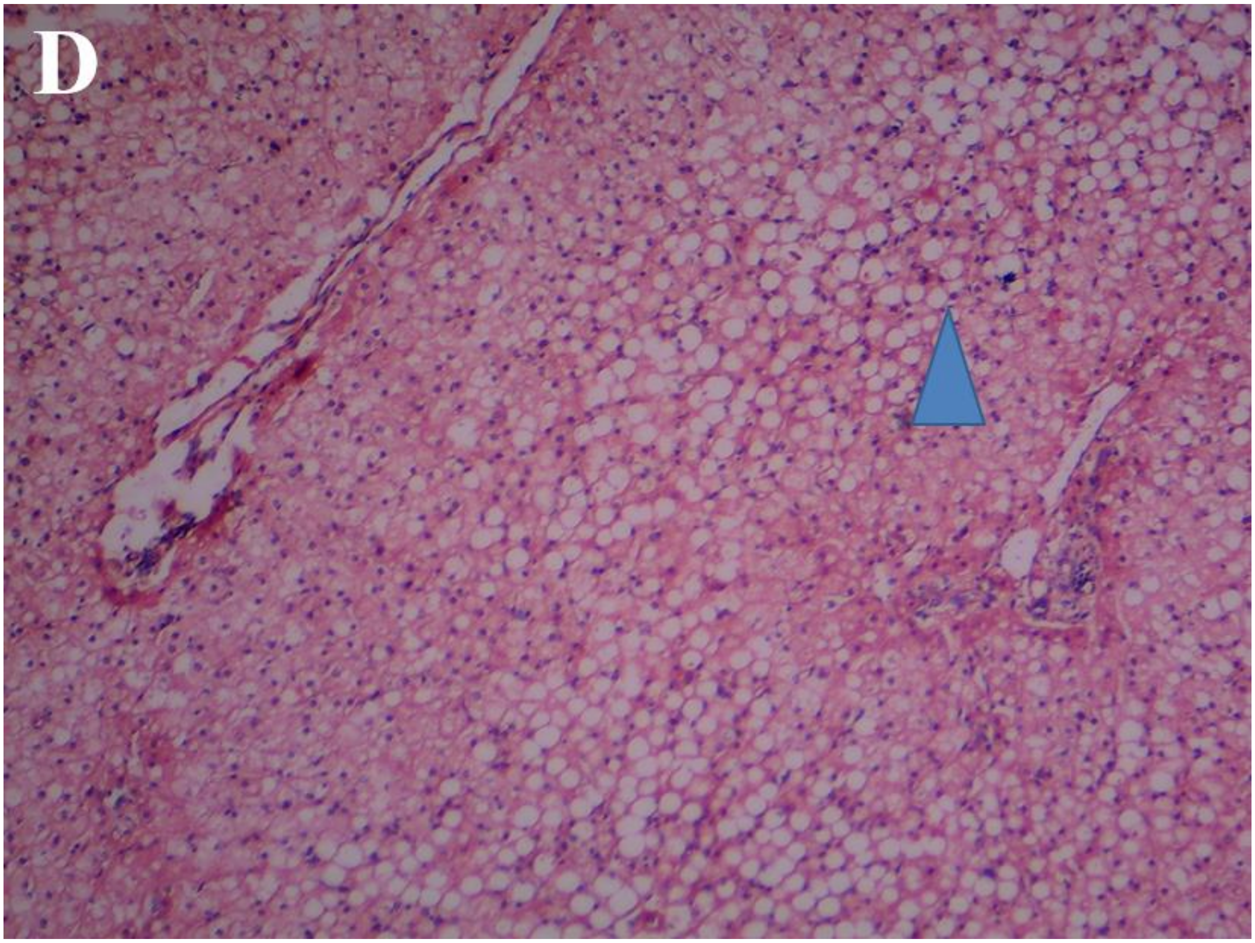
D

Figure

Click here to access/download;Figure;Figure 2.png

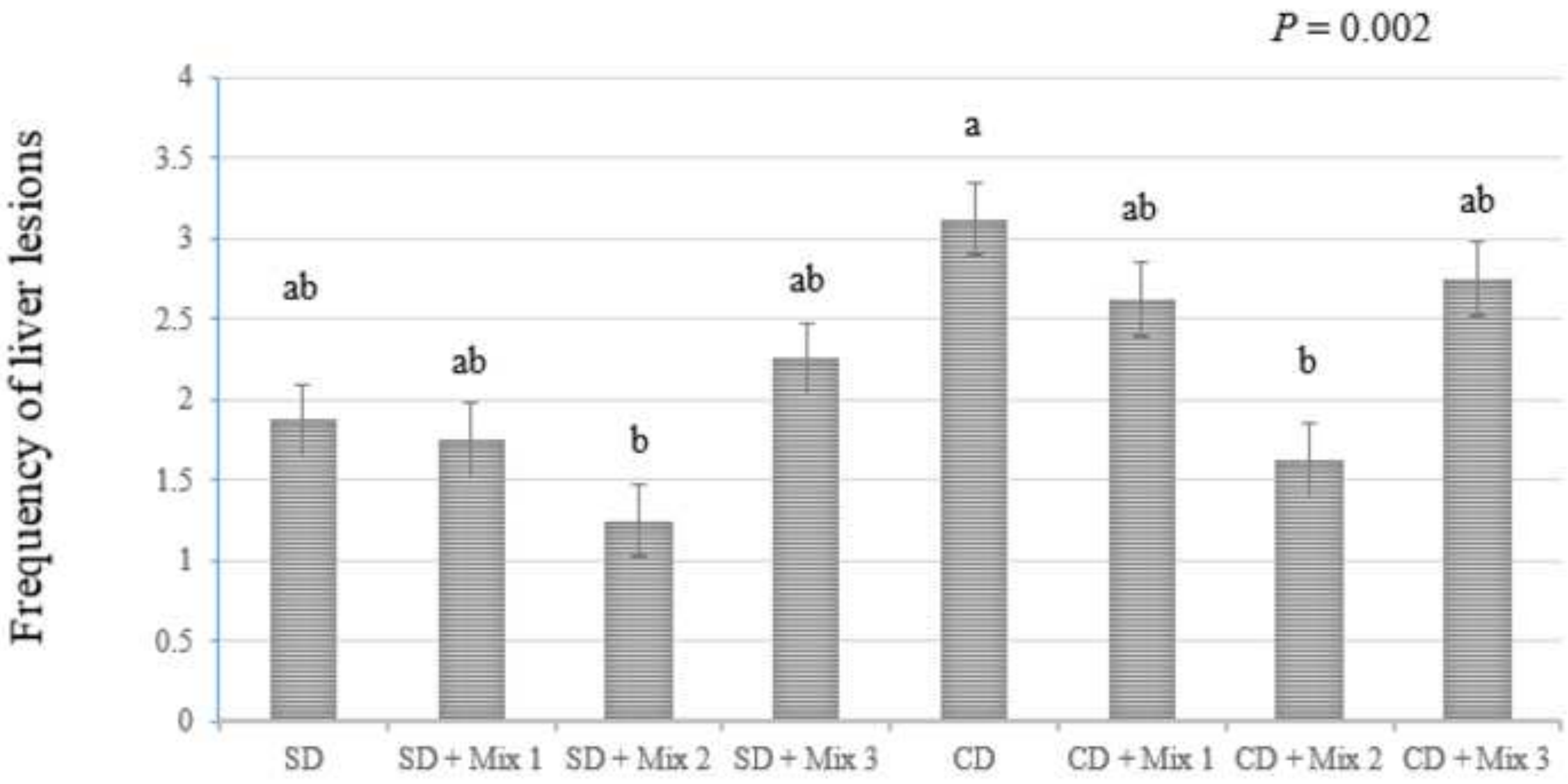

 Click here to access/download;Figure;Tables.docx

Table 1. Ingredients and composition of the treatment diets.

| **Ingredient (%)** | **SD** | **CD** |
|---|---|---|
| Corn | 57 | 66 |
| Soybean meal | 24 | 17 |
| Wheat bran | 6.4 | - |
| Soybean oil | 1 | 6 |
| Methionine | 0.15 | 0.15 |
| Salt | 0.2 | 0.2 |
| Sodium bicarbonate | 0.1 | 0.1 |
| Vitamin premix[a] | 0.25 | 0.25 |
| Mineral premix[b] | 0.25 | 0.25 |
| Oyster shell | 9.2 | 9.2 |
| Dicalcium phosphate | 1.5 | 1.5 |
| Multi enzyme containing phytase | 0.05 | 0.05 |
| **Nutrient composition** | | |
| ME (Kcal/kg) | 2500 | 3100 |
| Crude protein (%) | 15 | 12 |
| Crude fiber (%) | 2.1 | 2.1 |
| Calcium (%) | 3.7 | 3.7 |
| Sodium (%) | 0.13 | 0.13 |
| Available phosphorus (%) | 0.36 | 0.36 |

Treatment diets: SD (standard diet = normal protein and normal ME); CD (challenge diet = low protein and high ME)
[a] Vitamin mixture per 0.3 kg/100 kg of diet: Vitamin A, 7,700,000 IU; Vitamin D3, 3,300,000 IU; Vitamin E, 6600 mg; VitaminK3, 550 mg; thiamine,2200 mg; riboflavin, 4400 mg; Vitamin B6, 4400 mg; capantothenate, 550 mg; nicotinic acid, 200 mg; folic acid, 110 mg; choline chloride, 275,000 mg; biotin,55 mg; Vitamin B12, 8.8 mg. [b]Mineral mixture per 0.3 kg/100 kg of diet: Mn, 66,000 mg; Zn, 66,000 mg; Fe, 33,000 mg; Cu, 8,800 mg; Se, 300 mg.

Table 2. Effect of different combinations of herbal additives with standard and challenge diet on the feed intake of laying hens during weeks 1 to 12 of the trial (65 to 77 weeks of age)

| **Treatments** | Feed intake (g/hen/day) | | | | | | | | | | | |
|---|---|---|---|---|---|---|---|---|---|---|---|---|
| | Week1 | Week2 | Week3 | Week4 | Week5 | Week6 | Week7 | Week8 | Week9 | Week10 | Week11 | Week12 |
| **Main effects** | | | | | | | | | | | | |
| SD | 114.56 | 116.06 | 111.37 | 120.97 | 117.40$^{a}$ | 117.84$^{a}$ | 117.96$^{a}$ | 118.19$^{a}$ | 118.53$^{a}$ | 118.44$^{a}$ | 118.78$^{a}$ | 118.84$^{a}$ |
| CD | 114.40 | 116.40 | 110.69 | 118.62 | 105.34$^{b}$ | 103.62$^{b}$ | 102.84$^{b}$ | 103.31$^{b}$ | 96.87$^{b}$ | 96.87$^{b}$ | 96.87$^{b}$ | 96.87$^{b}$ |
| C | 113.44 | 114.31$^{b}$ | 110.75 | 117.00 | 110.44$^{b}$ | 108.81$^{b}$ | 107.19$^{c}$ | 107.62$^{c}$ | 103.50$^{c}$ | 103.31$^{c}$ | 104.00$^{c}$ | 104.12$^{b}$ |
| Mix 1 | 113.31 | 114.81$^{ab}$ | 110.31 | 119.62 | 109.50$^{b}$ | 109.50$^{b}$ | 109.50$^{bc}$ | 109.50$^{bc}$ | 104.37$^{c}$ | 104.37$^{bc}$ | 104.37$^{bc}$ | 104.37$^{b}$ |
| Mix 2 | 115.50 | 117.75$^{a}$ | 112.12 | 122.81 | 114.37$^{a}$ | 114.06$^{a}$ | 114.06$^{a}$ | 115.00$^{a}$ | 114.12$^{a}$ | 114.12$^{a}$ | 114.12$^{a}$ | 114.12$^{a}$ |
| Mix 3 | 115.69 | 118.06$^{a}$ | 110.94 | 119.75 | 111.19$^{b}$ | 110.56$^{b}$ | 110.87$^{b}$ | 110.87$^{b}$ | 108.81$^{b}$ | 108.81$^{b}$ | 108.81$^{b}$ | 108.81$^{b}$ |
| **SEM** | 12.01 | 20.13 | 22.21 | 55.17 | 15.20 | 11.81 | 17.36 | 18.07 | 37.24 | 39.97 | 40.07 | 40.23 |
| **Interactions** | | | | | | | | | | | | |
| SD | 113.13 | 112.13 | 110.88 | 116.25 | 115.13 | 116.88 | 117.38$^{a}$ | 118.25$^{a}$ | 117.13$^{a}$ | 116.75$^{a}$ | 118.13$^{a}$ | 118.38$^{a}$ |
| SD + Mix 1 | 113.75 | 116.63 | 110.38 | 123.25 | 116.50 | 116.50 | 116.50$^{a}$ | 116.50$^{a}$ | 116.88$^{a}$ | 116.88$^{a}$ | 116.88$^{a}$ | 116.88$^{a}$ |
| SD + Mix 2 | 115.00 | 116.88 | 112.38 | 121.75 | 119.38 | 119.38 | 119.38$^{a}$ | 119.38$^{a}$ | 120.00$^{a}$ | 120.00$^{a}$ | 120.00$^{a}$ | 120.00$^{a}$ |
| SD + Mix 3 | 116.38 | 118.63 | 111.88 | 122.63 | 118.63 | 118.63 | 118.63$^{a}$ | 118.63$^{a}$ | 120.13$^{a}$ | 120.13$^{a}$ | 120.13$^{a}$ | 120.13$^{a}$ |
| CD | 113.75 | 116.50 | 110.63 | 117.75 | 105.75 | 100.75 | 97.00$^{d}$ | 97.00$^{d}$ | 89.88$^{d}$ | 89.88$^{d}$ | 89.88$^{d}$ | 89.88$^{d}$ |
| CD + Mix 1 | 112.88 | 113.00 | 110.25 | 116.00 | 102.50 | 102.50 | 102.50$^{c}$ | 102.50$^{c}$ | 91.88$^{cd}$ | 91.88$^{cd}$ | 91.88$^{cd}$ | 91.88$^{cd}$ |
| CD + Mix 2 | 116.00 | 118.63 | 111.88 | 123.88 | 109.38 | 108.75 | 108.75$^{b}$ | 110.63$^{b}$ | 108.25$^{b}$ | 108.25$^{b}$ | 108.25$^{b}$ | 108.25$^{b}$ |
| CD + Mix 3 | 115.00 | 117.50 | 110.00 | 116.88 | 103.75 | 102.50 | 103.13$^{c}$ | 103.13$^{c}$ | 97.50$^{c}$ | 97.50$^{c}$ | 97.50$^{c}$ | 97.50$^{c}$ |
| **SEM** | 3.46 | 4.49 | 4.71 | 7.43 | 3.90 | 3.44 | 4.16 | 4.25 | 6.10 | 6.32 | 6.33 | 6.34 |
| **Significant levels** | | | | | | | | | | | | |
| SDCD | 0.8575 | 0.7604 | 0.5619 | 0.2121 | 0.0001 | 0.0001 | 0.0001 | 0.0001 | 0.0001 | 0.0001 | 0.0001 | 0.0001 |
| HA | 0.0985 | 0.0377 | 0.7306 | 0.1908 | 0.0053 | 0.0003 | 0.0002 | 0.0001 | 0.0001 | 0.0001 | 0.0001 | 0.0001 |
| SDCD*HA | 0.7264 | 0.0780 | 0.9496 | 0.1771 | 0.1202 | 0.0889 | 0.0153 | 0.0015 | 0.0036 | 0.0061 | 0.0034 | 0.0031 |

SD=standard diet; CD=challenge diet; C=control (without additive); Mix 1= turmeric, fumitory, green tea and milk thistle powder; Mix 2= lemon, black pepper, sumac and chicory powder; Mix 3= garlic, artichoke, ginger and shallot powder; HA= herbal additive; SDCD*HA= interaction effect of dietary treatments.

a–d Means with no common superscript within each column are significantly ($P < 0.05$) different.

SEM= standard error of the mean.

Table 3. Effect of different combinations of herbal additives with standard and challenge diet on the egg production ratio (%) of laying hens during weeks 1 to 12 of the trial (65 to 77 weeks of age)

| **Treatments** | **Hen-day egg production (%)** | | | | | | | | | | | |
|---|---|---|---|---|---|---|---|---|---|---|---|---|
| | Week1 | Week2 | Week3 | Week4 | Week5 | Week6 | Week7 | Week8 | Week9 | Week10 | Week11 | Week12 |
| **Main effects** | | | | | | | | | | | | |
| SD | 88.37 | 86.68 | 85.03 | 85.81 | 84.84[a] | 84.37[a] | 83.78[a] | 83.41[a] | 82.09[a] | 81.25[a] | 80.69[a] | 80.40[a] |
| CD | 88.58 | 86.86 | 85.05 | 85.68 | 75.59[b] | 75.50[b] | 75.50[b] | 75.53[b] | 71.66[b] | 71.28[b] | 70.34[b] | 70.34[b] |
| C | 88.44 | 87.20 | 85.53 | 86.42 | 80.87[b] | 79.87[b] | 79.75[b] | 79.25[b] | 75.12[bc] | 73.19[c] | 71.81[b] | 71.25[b] |
| Mix 1 | 88.67 | 86.69 | 85.03 | 85.61 | 78.56[c] | 78.56[b] | 78.31[bc] | 78.31[b] | 74.00[c] | 73.50[c] | 72.69[b] | 72.69[b] |
| Mix 2 | 88.00 | 86.64 | 84.84 | 85.64 | 83.31[a] | 83.25[a] | 82.87[a] | 82.81[a] | 80.69[a] | 80.69[a] | 80.31[a] | 80.31[a] |
| Mix 3 | 88.81 | 86.55 | 84.75 | 85.31 | 78.12[c] | 78.06[c] | 77.62[c] | 77.50[b] | 77.69[b] | 77.69[b] | 77.25[a] | 77.25[a] |
| **SEM** | 1.83 | 1.79 | 1.63 | 1.43 | 5.76 | 5.87 | 5.86 | 6.23 | 15.89 | 16.76 | 20.51 | 20.89 |
| **Interactions** | | | | | | | | | | | | |
| SD | 88.62 | 87.40 | 85.69 | 86.87 | 86.75[a] | 85.12[a] | 84.87[a] | 83.75[a] | 84.75[a] | 81.37[a] | 80.12[a] | 79.00[a] |
| SD + Mix 1 | 88.37 | 86.12 | 84.50 | 85.19 | 84.00[b] | 84.00[ab] | 83.50[a] | 83.50[a] | 81.12[a] | 81.12[a] | 80.50[a] | 80.50[a] |
| SD + Mix 2 | 87.75 | 86.31 | 85.06 | 85.94 | 84.25[ab] | 84.12[ab] | 83.37[a] | 83.25[a] | 81.00[a] | 81.00[a] | 80.75[a] | 80.75[a] |
| SD + Mix 3 | 88.75 | 86.87 | 84.87 | 85.26 | 84.37[ab] | 84.25[ab] | 83.37[a] | 83.12[a] | 81.50[a] | 81.50[a] | 81.37[a] | 81.37[a] |
| CD | 88.25 | 87.00 | 85.37 | 85.97 | 75.00[c] | 74.62[c] | 74.62[b] | 74.75[b] | 65.50[c] | 65.00[c] | 63.50[c] | 63.50[c] |
| CD + Mix 1 | 88.97 | 87.25 | 85.56 | 86.04 | 73.12[cd] | 73.12[cd] | 73.12[bc] | 73.12[bc] | 66.87[c] | 65.87[c] | 64.87[c] | 64.87[c] |
| CD + Mix 2 | 88.25 | 86.97 | 84.62 | 85.34 | 82.37[b] | 82.37[b] | 82.37[a] | 82.37[a] | 80.37[a] | 80.37[a] | 79.87[a] | 79.87[a] |
| CD + Mix 3 | 88.86 | 86.22 | 84.62 | 85.36 | 71.87[d] | 71.87[d] | 71.87[c] | 71.87[c] | 73.87[b] | 73.87[b] | 73.12[b] | 73.12[b] |
| **SEM** | 1.82 | 1.78 | 1.63 | 1.43 | 5.75 | 2.42 | 2.42 | 2.50 | 3.99 | 4.09 | 4.53 | 4.57 |
| **Significant levels** | | | | | | | | | | | | |
| SDCD | 0.5383 | 0.5835 | 0.9612 | 0.6477 | 0.0001 | 0.0001 | 0.0001 | 0.0001 | 0.0001 | 0.0001 | 0.0001 | 0.0001 |
| HA | 0.3576 | 0.5193 | 0.3221 | 0.0667 | 0.0001 | 0.0001 | 0.0001 | 0.0001 | 0.0001 | 0.0001 | 0.0001 | 0.0001 |
| SDCD*HA | 0.7334 | 0.1988 | 0.3151 | 0.1774 | 0.0001 | 0.0001 | 0.0001 | 0.0001 | 0.0001 | 0.0001 | 0.0001 | 0.0001 |

SD=standard diet; CD=challenge diet; C=control (without additive); Mix 1= turmeric, fumitory, green tea and milk thistle powder; Mix 2= lemon, black pepper, sumac and chicory powder; Mix 3= garlic, artichoke, ginger and shallot powder; HA= herbal additive; SDCD*HA= interaction effect of dietary treatments.

a–d Means with no common superscript within each column are significantly ($P < 0.05$) different.

SEM= standard error of the mean.

Table 4. Effect of different combinations of herbal additives with standard and challenge diet on the egg weight (g) of laying hens during weeks 1 to 12 of the trial (65 to 77 weeks of age)

| **Treatments** | **Egg weight (g)** | | | | | | | | | | | |
|---|---|---|---|---|---|---|---|---|---|---|---|---|
| | Week1 | Week2 | Week3 | Week4 | Week5 | Week6 | Week7 | Week8 | Week9 | Week10 | Week11 | Week12 |
| **Main effects** | | | | | | | | | | | | |
| SD | 62.56 | 63.62 | 63.17 | 64.69 | 64.37[a] | 64.37[a] | 64.43[a] | 64.39[a] | 64.51[a] | 64.56[a] | 64.65[a] | 64.75[a] |
| CD | 62.47 | 63.58 | 63.36 | 65.25 | 61.75[b] | 61.09[b] | 61.00[b] | 61.09[b] | 61.19[b] | 60.87[b] | 60.81[b] | 61.00[b] |
| C | 62.56 | 63.31 | 62.69[b] | 64.94 | 62.59[b] | 62.03[b] | 61.91[b] | 61.87[b] | 62.16[b] | 62.16[b] | 62.31 | 62.50 |
| Mix 1 | 62.31 | 63.42 | 63.37[ab] | 64.20 | 62.53[b] | 61.84[b] | 61.91[b] | 62.09[b] | 62.14[b] | 62.06[b] | 62.16 | 62.21 |
| Mix 2 | 62.62 | 63.81 | 63.81[a] | 65.49 | 63.44[a] | 63.43[a] | 63.44[a] | 63.41[a] | 63.37[a] | 63.06[ab] | 62.94 | 63.12 |
| Mix 3 | 62.56 | 63.87 | 63.19[ab] | 65.25 | 63.69[a] | 63.62[a] | 63.62[a] | 63.59[a] | 63.72[a] | 63.59[a] | 63.53 | 63.69 |
| **SEM** | 1.73 | 1.06 | 1.21 | 2.05 | 1.31 | 1.81 | 1.83 | 1.83 | 2.20 | 3.59 | 4.28 | 4.21 |
| **Interactions** | | | | | | | | | | | | |
| SD | 63.25[a] | 63.50 | 62.50 | 64.50 | 63.93 | 63.93 | 64.18 | 64.00 | 64.06 | 64.31 | 64.50 | 64.87 |
| SD + Mix 1 | 61.62[b] | 63.12 | 63.31 | 64.27 | 63.69 | 63.69 | 63.69 | 63.68 | 63.66 | 63.62 | 63.68 | 63.66 |
| SD + Mix 2 | 62.87[ab] | 63.87 | 64.00 | 65.11 | 64.50 | 64.50 | 64.50 | 64.43 | 64.87 | 64.87 | 64.87 | 64.87 |
| SD + Mix 3 | 62.50[ab] | 64.00 | 62.87 | 64.87 | 65.37 | 65.37 | 65.37 | 65.43 | 65.44 | 65.44 | 65.56 | 65.62 |
| CD | 61.87[ab] | 63.12 | 62.87 | 65.37 | 61.25 | 60.12 | 59.62 | 59.75 | 60.25 | 60.00 | 60.12 | 60.12 |
| CD + Mix 1 | 63.00[ab] | 63.71 | 63.44 | 64.12 | 61.37 | 60.00 | 60.12 | 60.50 | 60.62 | 60.50 | 60.62 | 60.75 |
| CD + Mix 2 | 62.37[ab] | 63.75 | 63.62 | 65.87 | 62.37 | 62.37 | 62.37 | 62.37 | 61.87 | 61.25 | 61.00 | 61.37 |
| CD + Mix 3 | 62.62[ab] | 63.75 | 63.50 | 65.62 | 62.00 | 61.87 | 61.87 | 61.75 | 62.00 | 61.75 | 61.50 | 61.75 |
| **SEM** | 1.31 | 1.03 | 1.10 | 1.43 | 1.14 | 1.34 | 1.33 | 1.35 | 1.48 | 1.89 | 2.07 | 2.05 |
| **Significant levels** | | | | | | | | | | | | |
| SDCD | 0.7766 | 0.8754 | 0.4983 | 0.1240 | 0.0001 | 0.0001 | 0.0001 | 0.0001 | 0.0001 | 0.0001 | 0.0001 | 0.0001 |
| HA | 0.9111 | 0.3239 | 0.0445 | 0.0722 | 0.0094 | 0.0002 | 0.0002 | 0.0005 | 0.0044 | 0.0752 | 0.2298 | 0.1865 |
| SDCD*HA | 0.0342 | 0.5568 | 0.6163 | 0.7240 | 0.4319 | 0.2669 | 0.0925 | 0.1424 | 0.8498 | 0.8509 | 0.8302 | 0.6418 |

SD=standard diet; CD=challenge diet; C=control (without additive); Mix 1= turmeric, fumitory, green tea and milk thistle powder; Mix 2= lemon, black pepper, sumac and chicory powder; Mix 3= garlic, artichoke, ginger and shallot powder; HA= herbal additive; SDCD*HA= interaction effect of dietary treatments.

a–d Means with no common superscript within each column are significantly ($P < 0.05$) different.

SEM= standard error of the mean.

**Table 5**. Effect of different combinations of herbal additives with standard and challenge diet on the feed conversion ratio of laying hens during weeks 1 to 12 of the trial (65 to 77 weeks of age)

| Treatments | Feed conversion ratio (g/g) | | | | | | | | | | | |
|---|---|---|---|---|---|---|---|---|---|---|---|---|
| | Week1 | Week2 | Week3 | Week4 | Week5 | Week6 | Week7 | Week8 | Week9 | Week10 | Week11 | Week12 |
| **Main effects** | | | | | | | | | | | | |
| SD | 2.07 | 2.10 | 2.07 | 2.17 | $2.15^{b}$ | $2.17^{b}$ | 2.19 | 2.20 | 2.23 | 2.26 | 2.27 | 2.28 |
| CD | 2.06 | 2.11 | 2.05 | 2.12 | $2.26^{a}$ | $2.25^{a}$ | 2.24 | 2.24 | 2.22 | 2.24 | 2.28 | 2.28 |
| C | 2.051 | 2.071 | 2.068 | 2.081 | 2.191 | $2.199^{ab}$ | $2.168^{b}$ | 2.187 | 2.226 | 2.277 | 2.331 | 2.350 |
| Mix 1 | 2.054 | 2.091 | 2.050 | 2.175 | 2.233 | $2.259^{a}$ | $2.275^{a}$ | 2.262 | 2.268 | 2.290 | 2.312 | 2.306 |
| Mix 2 | 2.096 | 2.130 | 2.062 | 2.193 | 2.164 | $2.160^{b}$ | $2.168^{b}$ | 2.187 | 2.229 | 2.241 | 2.250 | 2.250 |
| Mix 3 | 2.084 | 2.136 | 2.075 | 2.150 | 2.242 | $2.232^{ab}$ | $2.262^{ab}$ | 2.262 | 2.203 | 2.207 | 2.225 | 2.218 |
| **SEM** | 0.008 | 0.011 | 0.011 | 0.025 | 0.013 | 0.010 | 0.016 | 0.015 | 0.034 | 0.037 | 0.044 | 0.046 |
| **Interactions** | | | | | | | | | | | | |
| SD | 2.018 | 2.021 | 2.087 | 2.075 | $2.076^{d}$ | $2.147^{cd}$ | $2.150^{b}$ | $2.200^{abc}$ | 2.158 | 2.236 | 2.275 | 2.312 |
| SD + Mix 1 | 2.090 | 2.145 | 2.050 | 2.250 | $2.178^{cd}$ | $2.178^{cd}$ | $2.212^{ab}$ | $2.212^{abc}$ | 2.263 | 2.264 | 2.275 | 2.275 |
| SD + Mix 2 | 2.085 | 2.120 | 2.050 | 2.175 | $2.197^{bcd}$ | $2.200^{bcd}$ | $2.225^{ab}$ | $2.225^{abc}$ | 2.283 | 2.283 | 2.287 | 2.287 |
| SD + Mix 3 | 2.100 | 2.134 | 2.100 | 2.212 | $2.151^{d}$ | $2.154^{cd}$ | $2.187^{b}$ | $2.187^{bc}$ | 2.253 | 2.253 | 2.250 | 2.250 |
| CD | 2.084 | 2.121 | 2.050 | 2.087 | $2.306^{ab}$ | $2.251^{abc}$ | $2.187^{b}$ | $2.175^{bc}$ | 2.294 | 2.317 | 2.387 | 2.387 |
| CD + Mix 1 | 2.017 | 2.038 | 2.050 | 2.100 | $2.287^{abc}$ | $2.341^{a}$ | $2.337^{a}$ | $2.312^{ba}$ | 2.273 | 2.316 | 2.350 | 2.337 |
| CD + Mix 2 | 2.108 | 2.140 | 2.075 | 2.212 | $2.132^{d}$ | $2.119^{d}$ | $2.112^{b}$ | $2.150^{c}$ | 2.176 | 2.199 | 2.212 | 2.212 |
| CD + Mix 3 | 2.067 | 2.138 | 2.050 | 2.087 | $2.334^{a}$ | $2.310^{ab}$ | $2.337^{a}$ | $2.337^{a}$ | 2.153 | 2.161 | 2.200 | 2.187 |
| **SEM** | 0.090 | 0.106 | 0.107 | 0.158 | 0.115 | 0.104 | 0.128 | 0.125 | 0.186 | 0.193 | 0.210 | 0.215 |
| **Significant levels** | | | | | | | | | | | | |
| SDCD | 0.8502 | 0.8783 | 0.5623 | 0.1610 | 0.0002 | 0.0019 | 0.1258 | 0.2352 | 0.7386 | 0.8243 | 0.7674 | 1.000 |
| HA | 0.4149 | 0.2614 | 0.9237 | 0.2131 | 0.1978 | 0.0516 | 0.0286 | 0.1368 | 0.7966 | 0.6151 | 0.4384 | 0.3268 |
| SDCD*HA | 0.1536 | 0.0618 | 0.7448 | 0.2418 | 0.0035 | 0.0048 | 0.0239 | 0.0467 | 0.2226 | 0.4632 | 0.5175 | 0.6516 |

SD=standard diet; CD=challenge diet; C=control (without additive); Mix 1= turmeric, fumitory, green tea and milk thistle powder; Mix 2= lemon, black pepper, sumac and chicory powder; Mix 3= garlic, artichoke, ginger and shallot powder; HA= herbal additive; SDCD*HA= interaction effect of dietary treatments.

a–d Means with no common superscript within each column are significantly ($P < 0.05$) different.

SEM= standard error of the mean.

**Table 6**. Effect of different combinations of herbal additives with standard and challenge diet on the blood parameters of laying hens (Start, Middle, End)

| **Treatments** | **AST(U/L)** | | | **TP(G/dL)** | | | **UA(mg/dl)** | | |
|---|---|---|---|---|---|---|---|---|---|
| | Start (wk 65) | Middle (wk 70) | End (wk 77) | Start (wk 65) | Middle (wk 70) | End (wk 77) | Start (wk 65) | Middle (wk 70) | End (wk 77) |
| **Main effects** | | | | | | | | | |
| SD | 213.57 | 224.78$^{b}$ | 232.97$^{b}$ | 6.41 | 6.79$^{a}$ | 6.90$^{a}$ | 6.18 | 6.19$^{b}$ | 6.56$^{b}$ |
| CD | 213.55 | 259.84$^{a}$ | 295.50$^{a}$ | 6.19 | 6.24$^{b}$ | 6.44$^{b}$ | 6.30 | 6.53$^{a}$ | 7.28$^{a}$ |
| C | 214.13 | 253.62$^{a}$ | 288.56$^{a}$ | 6.21 | 5.97$^{b}$ | 5.99$^{c}$ | 6.19 | 6.79$^{a}$ | 7.56$^{a}$ |
| Mix 1 | 212.78 | 253.31$^{a}$ | 279.31$^{a}$ | 6.35 | 6.42$^{b}$ | 6.53$^{b}$ | 6.19 | 6.71$^{a}$ | 7.14$^{b}$ |
| Mix 2 | 214.59 | 220.31$^{b}$ | 232.06$^{c}$ | 6.28 | 7.28$^{a}$ | 7.40$^{a}$ | 6.26 | 5.91$^{b}$ | 6.33$^{c}$ |
| Mix 3 | 212.75 | 242.00$^{a}$ | 257.00$^{b}$ | 6.37 | 6.40$^{b}$ | 6.77$^{b}$ | 6.32 | 6.03$^{b}$ | 6.65$^{c}$ |
| **SEM** | 70.06 | 469.77 | 531.16 | 0.279 | 0.559 | 0.430 | 0.122 | 0.262 | 0.332 |
| **Interactions** | | | | | | | | | |
| SD | 214.25 | 234.75$^{b}$ | 253.87$^{b}$ | 6.37 | 6.35$^{bcd}$ | 6.12$^{c}$ | 6.12 | 6.89$^{a}$ | 7.69$^{a}$ |
| SD + Mix 1 | 209.46 | 232.87$^{b}$ | 235.87$^{bc}$ | 6.70 | 7.02$^{ab}$ | 7.06$^{ab}$ | 6.17 | 6.80$^{a}$ | 6.91$^{b}$ |
| SD + Mix 2 | 218.94 | 225.62$^{bc}$ | 239.37$^{bc}$ | 6.46 | 7.12$^{ab}$ | 7.30$^{a}$ | 6.31 | 5.62$^{c}$ | 5.72$^{c}$ |
| SD + Mix 3 | 211.65 | 205.87$^{c}$ | 202.75$^{d}$ | 6.12 | 6.68$^{abc}$ | 7.12$^{a}$ | 6.13 | 5.44$^{c}$ | 5.94$^{c}$ |
| CD | 214.01 | 272.50$^{a}$ | 323.25$^{a}$ | 6.05 | 5.60$^{d}$ | 5.85$^{c}$ | 6.26 | 6.70$^{ab}$ | 7.43$^{ab}$ |
| CD + Mix 1 | 216.10 | 273.75$^{a}$ | 322.75$^{a}$ | 6.00 | 5.81$^{d}$ | 6.00$^{c}$ | 6.21 | 6.62$^{ab}$ | 7.37$^{ab}$ |
| CD + Mix 2 | 210.23 | 215.00$^{bc}$ | 224.75$^{cd}$ | 6.10 | 7.43$^{a}$ | 7.50$^{a}$ | 6.21 | 6.19$^{b}$ | 6.94$^{b}$ |
| CD + Mix 3 | 213.86 | 278.12$^{a}$ | 311.25$^{a}$ | 6.61 | 6.12$^{cd}$ | 6.41$^{bc}$ | 6.52 | 6.62$^{ab}$ | 7.37$^{ab}$ |
| **SEM** | 8.37 | 21.67 | 23.05 | 0.528 | 0.747 | 0.656 | 0.350 | 0.513 | 0.576 |
| **Significant levels** | | | | | | | | | |
| SDCD | 0.9917 | 0.0001 | 0.0001 | 0.0943 | 0.0046 | 0.0065 | 0.1853 | 0.0090 | 0.0001 |
| HA | 0.8956 | 0.0001 | 0.0001 | 0.8325 | 0.0001 | 0.0001 | 0.6854 | 0.0001 | 0.0001 |
| SDCD*HA | 0.0799 | 0.0001 | 0.0001 | 0.0870 | 0.0428 | 0.0511 | 0.2647 | 0.0006 | 0.0004 |

SD=standard diet; CD=challenge diet; C=control (without additive); Mix 1= turmeric, fumitory, green tea and milk thistle powder; Mix 2= lemon, black pepper, sumac and chicory powder; Mix 3= garlic, artichoke, ginger and shallot powder; HA= herbal additive; SDCD*HA= interaction effect of dietary treatments; AST= aspartate aminotransferase; TP= total protein; UA= uric acid.

a–d Means with no common superscript within each column are significantly ($P < 0.05$) different.

SEM= standard error of the mean.

**Table 7**. Effect of different combinations of herbal additives with standard and challenge diet on the blood parameters of laying hens (Start, Middle, End)

| Treatments | TG (mg/dL) | | | TC (mg/dL) | | | HDL(mg/dl) | | | LDL(mg/dl) | | |
|---|---|---|---|---|---|---|---|---|---|---|---|---|
| | Start (wk 65) | Middle (wk 70) | End (wk 77) | Start (wk 65) | Middle (wk 70) | End (wk 77) | Start (wk 65) | Middle (wk 70) | End (wk 77) | Start (wk 65) | Middle (wk 70) | End (wk 77) |
| **Main effects** | | | | | | | | | | | | |
| SD | 1304.28 | $1410.63^{b}$ | $1485.47^{b}$ | 210.29 | $199.70^{b}$ | $200.00^{b}$ | 5.39 | 6.35 | 6.61 | 46.42 | $52.03^{b}$ | 55.88 |
| CD | 1434.28 | $1580.88^{a}$ | $2611.97^{a}$ | 214.59 | $239.71^{a}$ | $275.19^{a}$ | 5.71 | 6.51 | 6.25 | 46.63 | $54.44^{a}$ | 56.21 |
| C | 1382.19 | 1503.31 | $2257.8^{a}$ | 213.64 | $230.12^{a}$ | $255.42^{a}$ | 5.07 | $5.70^{b}$ | $5.30^{c}$ | $46.15^{b}$ | 52.88 | 57.10 |
| Mix 1 | 1373.44 | 1473.75 | $2195.8^{a}$ | 215.12 | $233.00^{a}$ | $258.62^{a}$ | 5.41 | $5.91^{b}$ | $5.91^{bc}$ | $46.29^{ab}$ | 53.88 | 55.59 |
| Mix 2 | 1388.25 | 1518.75 | $1682.9^{b}$ | 209.71 | $221.47^{a}$ | $230.75^{b}$ | 5.87 | $6.59^{ab}$ | $6.58^{b}$ | $47.11^{a}$ | 53.41 | 56.25 |
| Mix 3 | 1333.00 | 1487.19 | $2058.5^{a}$ | 211.30 | $194.25^{b}$ | $205.56^{c}$ | 5.85 | $7.53^{a}$ | $7.93^{a}$ | $46.56^{ab}$ | 52.78 | 55.25 |
| **SEM** | 127.64 | 272.29 | 120.5 | 108.43 | 423.49 | 531.16 | 2.43 | 2.12 | 1.60 | 1.38 | 3.31 | 9.01 |
| **Interactions** | | | | | | | | | | | | |
| SD | 1305.50 | 1462.50 | $1518.87^{bc}$ | 211.00 | $225.12^{b}$ | $235.87^{c}$ | 5.01 | $5.15^{b}$ | $5.11^{c}$ | 45.90 | 52.50 | 58.37 |
| SD + Mix 1 | 1327.12 | 1371.87 | $1397.75^{c}$ | 211.50 | $231.62^{ab}$ | $233.37^{c}$ | 4.63 | $5.32^{b}$ | $5.56^{bc}$ | 46.43 | 52.12 | 55.27 |
| SD + Mix 2 | 1338.37 | 1453.12 | $1564.50^{bc}$ | 208.93 | $204.19^{c}$ | $203.37^{d}$ | 5.60 | $6.43^{b}$ | $6.60^{b}$ | 47.15 | 51.93 | 55.75 |
| SD + Mix 3 | 1245.62 | 1355.00 | $1460.75^{bc}$ | 209.75 | $137.87^{d}$ | $127.37^{e}$ | 6.32 | $8.50^{a}$ | $9.18^{a}$ | 46.22 | 51.56 | 54.12 |
| CD | 1458.87 | 1544.12 | $2996.62^{a}$ | 216.27 | $235.12^{ab}$ | $275.00^{ab}$ | 5.14 | $6.25^{b}$ | $5.49^{bc}$ | 46.41 | 53.26 | 55.83 |
| CD + Mix 1 | 1419.75 | 1575.62 | $2993.75^{a}$ | 218.75 | $234.37^{ab}$ | $283.87^{a}$ | 6.19 | $6.50^{b}$ | $6.26^{bc}$ | 46.13 | 55.63 | 55.90 |
| CD + Mix 2 | 1438.12 | 1584.37 | $1801.25^{b}$ | 210.49 | $238.75^{ab}$ | $258.12^{b}$ | 6.15 | $6.75^{b}$ | $6.56^{b}$ | 47.07 | 54.87 | 56.75 |
| CD + Mix 3 | 1420.37 | 1619.37 | $2656.25^{a}$ | 212.86 | $250.62^{a}$ | $283.75^{a}$ | 5.37 | $6.56^{b}$ | $6.68^{b}$ | 46.90 | 54.00 | 56.37 |
| **SEM** | 113.00 | 165.20 | 346.79 | 10.41 | 20.58 | 16.86 | 1.56 | 1.45 | 1.26 | 1.17 | 1.82 | 3.00 |
| **Significant levels** | | | | | | | | | | | | |
| SDCD | 0.0823 | 0.0001 | 0.0001 | 0.1044 | 0.0001 | 0.0001 | 0.4174 | 0.6572 | 0.2548 | 0.4916 | 0.0001 | 0.6576 |
| HA | 0.5119 | 0.8797 | 0.0001 | 0.4703 | 0.0001 | 0.0001 | 0.4163 | 0.0033 | 0.0001 | 0.1122 | 0.2993 | 0.3221 |
| SDCD*HA | 0.6787 | 0.4248 | 0.0001 | 0.8758 | 0.0001 | 0.0001 | 0.1651 | 0.0122 | 0.0028 | 0.5998 | 0.1785 | 0.1501 |

SD=standard diet; CD=challenge diet; C=control (without additive); Mix 1= turmeric, fumitory, green tea and milk thistle powder; Mix 2= lemon, black pepper, sumac and chicory powder; Mix 3= garlic, artichoke, ginger and shallot powder; HA= herbal additive; SDCD*HA= interaction effect of dietary treatments; TG=triglyceride; TC=total cholesterol; HDL=high density lipoprotein; LDL=Low density lipoprotein.

a–d Means with no common superscript within each column are significantly ($P < 0.05$) different.

SEM= standard error of the mean.

**Table 8**. Effect of different combinations of herbal additives with standard and challenge diet on the blood parameters of laying hens (Start, Middle, End)

| Treatments | Hb(g/dl) | | | Heterophile | | | Lymphocyte | | |
|---|---|---|---|---|---|---|---|---|---|
| | Start (wk 65) | Middle (wk 70) | End (wk 77) | Start (wk 65) | Middle (wk 70) | End (wk 77) | Start (wk 65) | Middle (wk 70) | End (wk 77) |
| **Main effects** | | | | | | | | | |
| SD | 19.41 | 19.72$^{b}$ | 20.69$^{b}$ | 25.84 | 26.54 | 27.77 | 73.03 | 79.95$^{b}$ | 82.36$^{b}$ |
| CD | 18.67 | 24.09$^{a}$ | 28.59$^{a}$ | 25.63 | 26.40 | 27.76 | 72.83 | 83.23$^{a}$ | 90.09$^{a}$ |
| C | 19.00 | 23.27$^{a}$ | 27.42$^{a}$ | 25.41 | 27.03$^{b}$ | 29.37$^{a}$ | 72.70 | 84.09$^{a}$ | 90.62$^{a}$ |
| Mix 1 | 18.94 | 23.18$^{a}$ | 27.34$^{a}$ | 26.05 | 28.50$^{a}$ | 29.94$^{a}$ | 72.53 | 83.77$^{ab}$ | 91.65$^{a}$ |
| Mix 2 | 17.61 | 21.27$^{b}$ | 21.31$^{b}$ | 25.64 | 25.25$^{c}$ | 26.24$^{b}$ | 72.93 | 77.58$^{c}$ | 77.62$^{c}$ |
| Mix 3 | 20.61 | 19.91$^{b}$ | 22.50$^{b}$ | 25.83 | 25.12$^{c}$ | 25.50$^{b}$ | 73.56 | 80.94$^{b}$ | 85.00$^{b}$ |
| **SEM** | 6.054 | 4.99 | 10.507 | 0.718 | 2.133 | 5.16 | 1.69 | 16.60 | 22.89 |
| **Interactions** | | | | | | | | | |
| SD | 21.00 | 23.56$^{b}$ | 25.60$^{bc}$ | 25.65 | 27.56$^{b}$ | 30.44$^{ab}$ | 73.00 | 84.37$^{a}$ | 88.12$^{b}$ |
| SD + Mix 1 | 18.61 | 22.74$^{b}$ | 25.17$^{c}$ | 26.11 | 30.50$^{a}$ | 31.51$^{a}$ | 73.25 | 83.54$^{a}$ | 89.81$^{ab}$ |
| SD + Mix 2 | 17.30 | 16.28$^{c}$ | 15.74$^{d}$ | 25.92 | 24.50$^{c}$ | 24.22$^{d}$ | 72.77 | 73.41$^{c}$ | 74.37$^{d}$ |
| SD + Mix 3 | 20.75 | 16.31$^{c}$ | 16.25$^{d}$ | 25.67 | 23.62$^{c}$ | 24.87$^{d}$ | 73.09 | 78.50$^{b}$ | 77.12$^{cd}$ |
| CD | 17.01 | 22.99$^{b}$ | 29.25$^{a}$ | 25.17 | 26.50$^{b}$ | 28.31$^{bc}$ | 72.40 | 83.81$^{a}$ | 93.12$^{ab}$ |
| CD + Mix 1 | 19.26 | 23.62$^{b}$ | 29.50$^{a}$ | 25.99 | 26.50$^{b}$ | 28.37$^{bc}$ | 71.81 | 84.00$^{a}$ | 93.50$^{a}$ |
| CD + Mix 2 | 17.92 | 26.25$^{a}$ | 26.87$^{abc}$ | 25.36 | 26.00$^{b}$ | 28.25$^{bc}$ | 73.09 | 81.75$^{ab}$ | 80.87$^{c}$ |
| CD + Mix 3 | 20.47 | 23.50$^{b}$ | 28.75$^{ab}$ | 25.99 | 26.62$^{b}$ | 26.12$^{cd}$ | 74.02 | 83.37$^{a}$ | 92.87$^{ab}$ |
| **SEM** | 2.46 | 2.21 | 3.24 | 0.847 | 1.46 | 2.27 | 1.30 | 4.07 | 4.78 |
| **Significant levels** | | | | | | | | | |
| SDCD | 0.2298 | 0.0001 | 0.0001 | 0.3201 | 0.7016 | 0.9956 | 0.5429 | 0.0021 | 0.0001 |
| HA | 0.0721 | 0.0001 | 0.0001 | 0.1904 | 0.0001 | 0.0001 | 0.1391 | 0.0001 | 0.0001 |
| SDCD*HA | 0.0923 | 0.0001 | 0.0002 | 0.4563 | 0.0001 | 0.0001 | 0.0632 | 0.0108 | 0.0030 |

SD=standard diet; CD=challenge diet; C=control (without additive); Mix 1= turmeric, fumitory, green tea and milk thistle powder; Mix 2= lemon, black pepper, sumac and chicory powder; Mix 3= garlic, artichoke, ginger and shallot powder; HA= herbal additive; SDCD*HA= interaction effect of dietary treatments; Hb=hemoglobin.

a–d Means with no common superscript within each column are significantly ($P < 0.05$) different.

SEM= standard error of the mean.

Click here to access/download
**Supporting Information**
Additional file 1.docx
