# Supplementary material for: Application of various mixtures of medicinal herbs in the diet of laying hens: Evaluating preventive approach of fatty liver syndrome
Source: PLoS One. 2025 Oct 24;20(10):e0330363. doi: 10.1371/journal.pone.0330363 (PMC12551855; doi:10.1371/journal.pone.0330363)
Supplement: Attachment — Submitted filename: PONE-D-25-15950_R1_reviewer.pdf [file pone.0330363.s006.pdf]

PLOS ONE

Application of various mixtures of medicinal herbs in the diet of laying hens: Evaluating preventive approach of fatty liver syndrome

--Manuscript Draft--

**Manuscript Number:**	PONE-D-25-15950R1
**Article Type:**	Research Article
**Full Title:**	Application of various mixtures of medicinal herbs in the diet of laying hens: Evaluating preventive approach of fatty liver syndrome
**Short Title:**	Dietary medicinal herbs to prevent fatty liver syndrome in laying hens
**Corresponding Author:**	Mehran Torki, Professor<br>Razi University<br>Kermanshah, IRAN, ISLAMIC REPUBLIC OF
**Keywords:**	Egg production; fatty liver; Laying Hen; Medicinal Herb
**Abstract:**	Fatty liver hemorrhagic syndrome (FLHS) is a major health issue in laying hens, which is associated with reduced productive performance and increased mortality. This study investigated the efficacy of three herbal additive mixes as dietary interventions to prevent FLHS in a total of 384 LSL-Lohmann laying hens from 65 to 77 weeks of age. Hens were allocated to eight treatments in a 2×4 factorial design, comprising two basal diets—a standard diet (SD) and a high-energy, low-protein challenge diet (CD)—alongside four dietary interventions (three herbal mixes and a control). The herbal mixes were formulated as follows: Mix 1 (turmeric, fumitory, green tea, milk thistle), Mix 2 (lemon, black pepper, sumac, chicory), and Mix 3 (garlic, artichoke, ginger, shallot). Productive performance, blood variables, and liver lesion scores were carefully assessed. The results demonstrated that the CD decreased feed intake (FI; P = 0.0001), egg production (EP; P = 0.0001), and egg weight (EW; P = 0.0001) from week 5 onward. Birds received the CD had poorer feed conversion ratio (FCR) as opposed to the SD in weeks 5 and 6 of the trial. Feeding the CD resulted in higher circulatory levels of aspartate aminotransferase (AST; P = 0.0001) and triglycerides (TG; P = 0.0001), and a higher frequency of severe livers scores (P = 0.002). Notably, Mix 2 emerged as the most effective intervention, significantly reversing the negative impacts of the CD across all measured parameters. However, the herbal mixes did not significantly affect heterophil and lymphocyte counts or hemoglobin (Hb) concentration when fed with the CD. Overall, the CD significantly impaired productive performance and increased liver pathological lesions. However, supplementing the CD with Mix 2 (lemon, black pepper, sumac, and chicory) consistently led to the most significant improvements across all measured parameters, proving to be an effective dietary intervention to prevent FLHS in laying hens.
**Order of Authors:**	Mohammadreza Khodaei, PhD
	Mehran Torki
	Fariborz Khajali
	Iraj Karimi, PhD
**Response to Reviewers:**	Reviewer #1: While the manuscript presents a well-designed experiment exploring the use of herbal additives to prevent FLHS in laying hens, the overall quality of English language and expression requires significant improvement. Numerous grammatical errors, awkward phrasings, and formatting inconsistencies—especially in the introduction and methods sections—may hinder reader comprehension. For instance, several sentences are either incomplete or improperly constructed, such as line 76–77, and citation formatting is occasionally unclear or broken. I recommend that the authors undergo thorough professional English language editing to enhance clarity, readability, and overall presentation before further consideration.<br>We have thoroughly reviewed the entire manuscript and carefully revised the text to address all grammatical errors, awkward phrasings, and formatting inconsistencies, particularly in the Introduction and Methods sections as noted. The sentence on lines 76–77 has been restructured for clarity. The citation formatting throughout the manuscript has been corrected. We have also sought assistance from a professional

English language editing service to ensure the manuscript meets high standards of readability and presentation.
Line 37, the author could change “background” to “introduction”, because “background” may be confused as the same one in Abstract.
The heading “Background” was changed to “Introduction”.
Lines 38–42: The point that the liver synthesizes lipids and is heavily burdened in hens is repeated. The author could try to combine them with a single sentence.
We have revised the text to eliminate redundancy and merged the information into a concise sentence (page 2, lines 40-44).
The methods mentioned in the manuscript should be cited formally. Such as Line 51-54.
We have carefully reviewed the entire Methods section to ensure all procedures, including those mentioned in lines 51–54, are properly cited with relevant and appropriate references.
Lines 55–64: The paragraph listing all plants and their bio-actives is dense and hard to read. The author could Use a table or bullet points for readability or briefly summarize: "These herbs are rich in flavonoids, alkaloids, sulfur compounds, and dietary fibers, with demonstrated antioxidant, anti-inflammatory, and lipid-lowering properties."
Thank you for your helpful suggestion. We have replaced the dense paragraph with a table summarizing the medicinal plants used and their key bioactive components (Table 1).
Lines 37-69, the author could use clearer topic sentences to guide the reader through each section logically, including liver function, disease description, economic impact, and herbal solutions.
We have revised lines 37–69 to include clearer and more distinct topic sentences at the beginning of each major section.
Lines 88–90, the author use informal phrasing “running into the experimental trial", which can be corrected to "A two-week adaptation period was implemented to acclimate hens to the diets prior to the 12-week experimental trial."
We have revised the sentence to eliminate informal phrasing.

For animal study design, the randomization or blinding details were missing. Please mark those in the experiment design part.
Thank you for highlighting this important point. In response, we have updated the experimental design section to include details regarding randomization and blinding (page 5, lines 89-90).
No mention of sample size calculation or statistical power. The author could revise as "Sample size was determined based on expected effect sizes from previous studies to achieve 80% power at α = 0.05."
We have revised the Methods section to include a statement on sample size calculation. The text now reads: “Sample size was determined based on expected effect sizes from previous studies to achieve 80% power at α = 0.05” (page 5, lines 88-89).
Reviewer #2: Dear Authors, Strictly follow my comments/corrections in the attached file.
1. In the introduction section, need for the study should be highlighted at the end followed by the hypothesis of the study

Thank you for your insightful comment. We have revised the final part of the Introduction to clearly emphasize the need for the study and to better establish the context and significance of our research. Additionally, we have now explicitly stated the hypothesis of the study to guide the reader and clarify our research objectives.

2. In methodology, clearly elaborate the sample size determination
We have revised this section to provide a clear explanation of how the sample size was determined (Page 5, lines 88-89).
3. Add appropriate citations
We have thoroughly reviewed the entire Methods section and ensured that all procedures are now properly cited with relevant and appropriate references.
4. In the results, improve readability and add actual p-value of each result
Thank you for your valuable comment. We have thoroughly revised the Results section to enhance clarity and improve readability. Additionally, we have included the actual p-values for all statistical comparisons rather than simply stating significance levels (e.g., $p < 0.05$).

Powered by Editorial Manager® and ProduXion Manager® from Aries Systems Corporation

	5. In the discussion, add logical reasoning of each result before discussing with the previous studies.<br>Thank you. We have revised the Discussion section to provide a clear and logical interpretation of each key result before comparing our findings with those of previous studies. These revisions aim to enhance the scientific depth and coherence of the discussion.<br>6. In the conclusion, briefly describe and add limitation of the study.<br>We have revised the Conclusion section to include a brief summary of the main findings, emphasizing the significance of our results. In addition, we have acknowledged the limitations of the study, such as the relatively short duration, the use of a single dose per herbal mixture, the lack of individual herb evaluation, and the restriction to one hen strain and age group. This addition provides a balanced perspective and suggests directions for future research.<br>Ad libitum feed was provided? at this age? why?<br>The CD was intentionally formulated to predispose hens to fatty liver hemorrhagic syndrome (FLHS). However, due to its imbalanced nutrient profile, feed intake could be influenced as hens attempt to compensate for nutrient deficiencies. By allowing ad libitum access, we enabled the hens to express natural compensatory feeding behavior, which is crucial for observing true diet-induced metabolic and hepatic changes.
**Additional Information:**	
**Question**	**Response**
**Financial Disclosure**<br><br>Enter a financial disclosure statement that describes the sources of funding for the work included in this submission. Review the submission guidelines for detailed requirements. View published research articles from *PLOS ONE* for specific examples.<br><br>This statement is required for submission and **will appear in the published article** if the submission is accepted. Please make sure it is accurate.<br><br>**Funded studies**<br>Enter a statement with the following details:<br>• Initials of the authors who received each award<br>• Grant numbers awarded to each author<br>• The full name of each funder<br>• URL of each funder website<br>• Did the sponsors or funders play any role in the study design, data collection and analysis, decision to publish, or preparation of the manuscript?<br><br>Did you receive funding for this work?	The author(s) received no specific funding for this work.
**Competing Interests**	The authors have declared that no competing interests exist.

Use the instructions below to enter a competing interest statement for this submission. On behalf of all authors, disclose any competing interests that could be perceived to bias this work—acknowledging all financial support and any other relevant financial or non-financial competing interests.<br><br>This statement is required for submission and **will appear in the published article** if the submission is accepted. Please make sure it is accurate and that any funding sources listed in your Funding Information later in the submission form are also declared in your Financial Disclosure statement.<br><br>View published research articles from *PLOS ONE* for specific examples.<br><br>**NO authors have competing interests**<br><br>Enter: *The authors have declared that no competing interests exist.*<br><br>**Authors with competing interests**<br><br>Enter competing interest details beginning with this statement:<br><br>*I have read the journal's policy and the authors of this manuscript have the following competing interests: [insert competing interests here]*<br><br>* typeset	
**Ethics Statement**<br><br>Enter an ethics statement for this submission. This statement is required if the study involved:<br><br>• Human participants<br>• Human specimens or tissue<br>• Vertebrate animals or cephalopods<br>• Vertebrate embryos or tissues	The Animal Ethics Committee of Razi University, Kermanshah, Iran approved guidelines were followed for all experimental protocols (IR.RAZI.REC.1400.051). These guidelines adhered to the EU standards for the protection of animals and/or feed legislation

• Field research<br><br>Write "N/A" if the submission does not require an ethics statement.<br><br>General guidance is provided below. Consult the submission guidelines for detailed instructions. **Make sure that all information entered here is included in the Methods section of the manuscript.**	

Powered by Editorial Manager® and ProduXion Manager® from Aries Systems Corporation

**Format for specific study types**<br><br>**Human Subject Research (involving human participants and/or tissue)**<br>• Give the name of the institutional review board or ethics committee that approved the study<br>• Include the approval number and/or a statement indicating approval of this research<br>• Indicate the form of consent obtained (written/oral) or the reason that consent was not obtained (e.g. the data were analyzed anonymously)<br><br>**Animal Research (involving vertebrate animals, embryos or tissues)**<br>• Provide the name of the Institutional Animal Care and Use Committee (IACUC) or other relevant ethics board that reviewed the study protocol, and indicate whether they approved this research or granted a formal waiver of ethical approval<br>• Include an approval number if one was obtained<br>• If the study involved *non-human primates*, add *additional details* about animal welfare and steps taken to ameliorate suffering<br>• If anesthesia, euthanasia, or any kind of animal sacrifice is part of the study, include briefly which substances and/or methods were applied<br><br>**Field Research**<br><br>Include the following details if this study involves the collection of plant, animal, or other materials from a natural setting:<br>• Field permit number<br>• Name of the institution or relevant body that granted permission	
**Data Availability**<br><br>Authors are required to make all data underlying the findings described fully available, without restriction, and from the time of publication. PLOS allows rare exceptions to address legal and ethical concerns. See the PLOS Data Policy and FAQ for detailed information.	Yes - all data are fully available without restriction

A Data Availability Statement describing where the data can be found is required at submission. Your answers to this question constitute the Data Availability Statement and **will be published in the article**, if accepted.<br><br>**Important:** Stating 'data available on request from the author' is not sufficient. If your data are only available upon request, select 'No' for the first question and explain your exceptional situation in the text box.<br><br>Do the authors confirm that all data underlying the findings described in their manuscript are fully available without restriction?	
**Describe where the data may be found in full sentences. If you are copying our sample text, replace any instances of XXX with the appropriate details.**<br><br>• If the data are **held or will be held in a public repository**, include URLs, accession numbers or DOIs. If this information will only be available after acceptance, indicate this by ticking the box below. For example: *All XXX files are available from the XXX database (accession number(s) XXX, XXX.).*<br>• If the data are all contained **within the manuscript and/or Supporting Information files**, enter the following: *All relevant data are within the manuscript and its Supporting Information files.*<br>• If neither of these applies but you are able to provide **details of access elsewhere**, with or without limitations, please do so. For example:<br><br>*Data cannot be shared publicly because of [XXX]. Data are available from the XXX Institutional Data Access / Ethics Committee (contact via XXX) for researchers who meet the criteria for access to confidential data.*<br><br>*The data underlying the results presented in the study are available from (include the name of the third party*	All relevant data are within the manuscript and its Supporting Information files.

Powered by Editorial Manager® and ProduXion Manager® from Aries Systems Corporation

and contact information or URL).<br>• This text is appropriate if the data are owned by a third party and authors do not have permission to share the data.<br><br>* typeset	
Additional data availability information:	

Powered by Editorial Manager® and ProduXion Manager® from Aries Systems Corporation

Manuscript Click here to access/download;Manuscript;Manuscript.docx

Application of various mixtures of medicinal herbs in the diet of laying hens: Evaluating preventive approach of fatty liver syndrome

Mohammadreza Khodaei[1], Mehran Torki[1*], Fariborz Khajali[2], Iraj Karimi[3]

[1] Department of Animal Science, College of Agriculture and Natural Resources, Razi University, Kermanshah, Iran, Postal Code: 67156-85423

[2] Department of Animal Science, Shahrekord University, Shahrekord, Iran, Postal Code: 88186-34141

[3] Department of Pathology, Faculty of Veterinary Medicine, Shahrekord University, Shahrekord, Iran

[*] Corresponding author: torki@razi.ac.ir

Running title: Dietary medicinal herbs to prevent fatty liver syndrome in laying hens

Abstract

Fatty liver hemorrhagic syndrome (FLHS) is a major health issue in laying hens, which is associated with reduced productive performance and increased mortality. This study investigated the efficacy of three herbal additive mixes as dietary interventions to prevent FLHS in a total of 384 LSL-Lohmann laying hens from 65 to 77 weeks of age. Hens were allocated to eight treatments in a 2×4 factorial design, comprising two basal diets—a standard diet (SD) and a high-energy, low-protein challenge diet (CD)—alongside four dietary interventions (three herbal mixes

and a control). The herbal mixes were formulated as follows: Mix 1 (turmeric, fumitory, green tea, milk thistle), Mix 2 (lemon, black pepper, sumac, chicory), and Mix 3 (garlic, artichoke, ginger, shallot). Productive performance, blood variables, and liver lesion scores were carefully assessed. The results demonstrated that the CD decreased feed intake (FI; P = 0.0001), egg production (EP; P = 0.0001), and egg weight (EW; P = 0.0001) from week 5 onward. Birds received the CD had poorer feed conversion ratio (FCR) as opposed to the SD in weeks 5 and 6 of the trial. Feeding the CD resulted in higher circulatory levels of aspartate aminotransferase (AST; P = 0.0001) and triglycerides (TG; P = 0.0001), and a higher frequency of severe livers scores (P = 0.002). Notably, Mix 2 emerged as the most effective intervention, significantly reversing the negative impacts of the CD across all measured parameters. However, the herbal mixes did not significantly affect heterophil and lymphocyte counts or hemoglobin (Hb) concentration when fed with the CD. Overall, the CD significantly impaired productive performance and increased liver pathological lesions. However, supplementing the CD with Mix 2 (lemon, black pepper, sumac, and chicory) consistently led to the most significant improvements across all measured parameters, proving to be an effective dietary intervention to prevent FLHS in laying hens.

Keywords: Egg Production; Fatty Liver; Laying Hen; Medicinal Herb

Introduction

A typical layer diet provides approximately 2-4 g of lipids per day, which is considerably less than the amount required for body weight gain and egg formation [1]. In birds, lipids are primarily synthesized in the liver. As a result, there is a heavy burden on the liver to form necessary lipids. Consequently, dealing with an enormous amount of lipids predisposes laying hens to fatty liver hemorrhagic syndrome (FLHS) [2, 3].

FLHS is a non-infectious disease characterized by the excessive accumulation of fat in the liver and abdominal cavity. This significant fat buildup can lead to hepatocyte rupture, internal bleeding, and sudden death in affected birds [4, 5]. FLHS is more commonly observed in laying hens that are housed in cage systems [5, 6]. Affected flocks suffer substantial economic losses due to decreased egg production (EP) and increased mortality rates [4, 7]. During necropsy, birds that died from FLHS exhibit enlarged, soft, yellow, and fatty livers. This condition makes the liver more susceptible to tearing and bleeding [3, 5]. The exact cause of FLHS remains unclear, but several factors are thought to contribute. These include nutritional factors like excess dietary energy, fungal toxins, and anti-nutritional substances [2, 8, 9], as well as genetic predisposition, and environmental factors such as ambient temperature and rearing system [5, 10]. It is likely that a combination of these factors may contribute to the development of FLHS.

Given the multifactorial nature of FLHS, various nutritional strategies have been explored to mitigate its impact—particularly those aimed at reducing hepatic lipid synthesis [6]. Among these, medicinal plants have emerged as a promising approach due to their bioactive compounds with health-promoting properties [11–21]. These compounds often include flavonoids, alkaloids, sulfur compounds, and dietary fibers, all of which possess documented antioxidant, anti-inflammatory, and lipid-lowering properties [22]. Specific examples of these plants and their key bioactive constituents are summarized in Table 1.

In addition to reducing economic losses, herbal interventions align with animal welfare priorities by addressing the pain and suffering associated with FLHS [4, 7]. As global egg demand rises, enhancing the health and productivity of laying hens is vital for sustainable poultry production. Therefore, developing effective dietary strategies remains a key research priority.

While individual medicinal herbs have been examined for their potential benefits in laying hens, the efficacy of combinations of these herbal additives in preventing or mitigating FLHS has not been thoroughly investigated. Thus, the objectives of the present study were to develop an appropriate formulation of herbal additives in laying hen diets to evaluate productive responses, blood parameters, and the development of FLHS.

Table 1. Medicinal plants and their key bioactive compounds

**Plant**	**Scientific name**	**Main bioactive compounds**	**References**
Sumac	*Rhus coriaria*	Flavonoids, anthocyanins	[11]
Lemon	*Citrus limon*	Vitamin C, flavonoids, limonoids	[12]
Black pepper	*Piper nigrum*	Piperine alkaloids	[13]
Chicory	*Cichorium intybus L.*	Inulin, sesquiterpenes	[14]
Milk thistle	*Silybum marianum*	Silymarin (flavonoid complex)	[15]
Garlic / Shallot	*Allium sativum, A. cepa*	Allicin (organosulfur compound)	[16]
Artichoke	*Cynara scolymus*	Inulin, oligofructose	[17]
Green tea	*Camellia sinensis*	Catechins (flavonoids)	[18]
Turmeric	*Curcuma longa*	Curcumin	[19]
Fumitory	*Fumaria officinalis*	Protopine, fumaric acid	[20]
Ginger	*Zingiber officinale*	Gingerol, gingerdiol, gingerdione	[21]

Materials and methods

Ethics approval

The Animal Ethics Committee of Razi University, Kermanshah, Iran approved guidelines were followed for all experimental protocols (IR.RAZI.REC.1400.051). These guidelines adhered to the EU standards for the protection of animals and/or feed legislation.

Experimental design and dietary treatments

A number of 1000 fertile eggs (60.5 ± 3.5 g, Lohmann LSL-Lite strain) were obtained from a local breeding company (Toyoor-Barekat, Tehran, Iran). Fertile eggs were set in the laboratory incubator (MG-1000 model, Rcom Co., Ltd., Korea) of the Animal Science Department, Razi University, Kermanshah, Iran. Hatched chicks were then raised according to the strain guideline until the onset of the experimental trail at the age of 65 weeks [23]. A total of 384 laying hens were randomly selected from the flock. In doing so, all hens were weighed to obtain an average body weight in the flock (1,605 ± 85 g). The hens were housed in wire cages measuring 45 × 45 × 45 cm, with three birds per cage, corresponding to a stocking density of 675 cm^2 per bird. Two adjacent cages (containing six birds in total) were considered one replicate, resulting in eight replicates per treatment. Sample size was determined based on expected effect sizes from previous studies to achieve 80% power at $\alpha = 0.05$. To minimize potential bias, all data collection and subsequent laboratory analyses were performed by personnel blinded to the dietary treatments. A 16-hour light/8-hour dark lighting schedule was implemented using white LED lighting, providing an intensity of approximately 10–15 lux at bird level. The temperature-humidity index (THI) over the 12-week period is presented in Fig 1. Hens had free access to feed and water throughout the experiment.

Fig 1. Variation in temperature-humidity index (THI), temperature (°C), and relative humidity (%) over a 12-week period. The THI was calculated using the equation proposed by Marai et al. [24]:

THI = db°F – [(0.55 – 0.55 × RH) × (db°F – 58)], where RH represents the relative humidity (%) and db°F is the dry-bulb temperature in degrees Fahrenheit.

Two basal diets were formulated for the experiment: a standard diet (SD) formulated according to the strain's recommended guidelines (2500 kcal/kg AME and 15% crude protein) [23], and a challenge diet (CD) having high energy and low protein contents (3100 kcal/kg AME and 12% crude protein) designed to induce FLHS. Within each basal diet, there were four dietary interventions including three herbal mixes along with a control with no herbal additives. Mix 1 included turmeric, fumitory, green tea, and milk thistle. Mix 2 contained lemon, black pepper, sumac, and chicory. Mix 3 comprised garlic, artichoke, ginger, and shallot. The experimental diets and their chemical composition are presented in Table 2. A two-week adaptation period was implemented to acclimate hens to the diets prior to the 12-week experimental trial. The adaptation period was considered to minimize the error effect caused by the changes in the diets. The experimental trial lasted for 12 weeks (weeks 65 through 77).

Table 2. Ingredients and composition of the treatment diets.

**Ingredients (%)**	**SD**	**CD**
Corn	57	66
Soybean meal	24	17
Wheat bran	6.4	-
Soybean oil	1	6
Methionine	0.15	0.15
Salt	0.2	0.2
Sodium bicarbonate	0.1	0.1

Vitamin premix[a]	0.25	0.25
Mineral premix[b]	0.25	0.25
Oyster shell	9.2	9.2
Dicalcium phosphate	1.5	1.5
Multi enzyme containing phytase	0.05	0.05
**Nutrient composition**		
ME (Kcal/kg)	2500	3100
Crude protein (%)	15	12
Crude fiber (%)	2.1	2.1
Calcium (%)	3.7	3.7
Sodium (%)	0.13	0.13
Available phosphorus (%)	0.36	0.36

Treatment diets: SD (standard diet = normal protein and normal ME); CD (challenge diet = low protein and high ME)

[a] Vitamin mixture per 0.3 kg/100 kg of diet: Vitamin A, 7,700,000 IU; Vitamin D3, 3,300,000 IU; Vitamin E, 6600 mg; VitaminK3, 550 mg; thiamine,2200 mg; riboflavin, 4400 mg; Vitamin B6, 4400 mg; capantothenate, 550 mg; nicotinic acid, 200 mg; folic acid, 110 mg; choline chloride, 275,000 mg; biotin,55

mg; Vitamin B12, 8.8 mg. [b]Mineral mixture per 0.3 kg/100 kg of diet: Mn, 66,000 mg; Zn, 66,000 mg; Fe, 33,000 mg; Cu, 8,800 mg; Se, 300 mg.

Measurements

Gas chromatography-mass spectrometry (GC-MS)

Volatile compounds of herbal samples were identified using a headspace (HS) sampling technique followed by GC-MS analysis. A qualitative GC-MS analysis was performed with an HP 6890 gas chromatograph interfaced with an HP 5975C Mass Selective Detector (Agilent Technologies, Foster City, CA, USA). The analysis employed electron ionization (EI) mode at 70 eV and followed the procedure described by Naghneh et al. [25]. The composition of herbs has shown in supplementary file 1.

Productive performance

Egg count and weight were recorded on a daily basis, and these data were used to calculate hen-day egg production (EP), and egg weight (EW). Moreover, feed conversion ratio (FCR) was calculated based on feed intake (FI) and egg performance data [26].

Blood parameters

At weeks 65 (the start), 70 (the middle) and 77 (the end of trial), two laying hens from each replicate (16 hens per treatment) were randomly selected for blood collection from the wing vein. A sample of 3 ml blood was collected in a test tube without any anticoagulant. Blood samples were immediately centrifuged at 3000 rpm for 10 minutes and the resulting sera were stored at -20°C until laboratory tests. The following parameters were determined according to the manufacturer's instructions using commercial diagnostic kits (Pars Azmun, Iran): aspartate aminotransferase (AST), total protein (TP), total triglyceride (TG), total cholesterol (TC), low-density cholesterol

(LDL), high-density cholesterol (HDL), uric acid (UA), and hemoglobin (Hb) (Parsazmun, Tehran, Iran) [27]. In addition, a smear of blood was prepared from each blood sample to count heterophiles and lymphocytes.

Hepatic histopathology and scoring system

During the experimental period, all mortalities were weighed and necropsied, and the incidence of FLHS was assessed in all treatment groups. At the end of trial (Week 77), one hen from each cage (8 hens per treatment) were randomly selected, anaesthetized via inhalation of 60% CO_2, and then sacrificed by cervical dislocation. CO_2 was delivered from compressed gas canister and delivered using a gradual fill method with a displacement rate of 60% via the chamber volume per minute using of a flowmeter. The livers were harvested and dissected for further analysis. To assess histological examinations, liver tissues were fixed in 10% buffered formalin and processed by a microtome. These sections were then stained with hematoxylin and eosin (H&E) before examination under a light microscope [28]. A four-point scoring system was employed to evaluate the severity of FLHS in the chicken livers, based on the presence and extent of acentric nuclei within hepatocytes. Acentric nuclei are displaced from their central position due to the accumulation of fat droplets within the cells. Scores were ranged from 1 (indicating normal liver with centrally located nuclei) to 4 (representing severe FLHS with extensive acentric nuclei), with 2 and 3 signifying mild and moderate degrees of acentricity, respectively (Fig 2).

Fig 2. Representative liver histopathology under H&E staining at increasing grades of fatty change. (A) Normal liver shows uniform architecture with centrally located nuclei (score 1). (B) Mild fatty change (score 2) exhibits minimal fatty vacuoles (clear spaces) within hepatocytes. (C) Moderate fatty change (score 3) demonstrates increased cytoplasmic vacuolization. (D) Severe

fatty change (score 4) displays extensive fatty infiltration obscuring most cytoplasm. Arrowhead highlights an acentric nucleus, a characteristic finding in steatosis.

Statistical analysis

The study was conducted in a completely randomized design using a 2×4 factorial layout. Data were subjected to the GLM procedures of SAS 9.4 software [29] according to the following model:

$Y_{ijk} = \mu + A_i + B_j + ABij + e_{ijk}$

where Y_{ijk} = observed value for a particular trait, μ= overall mean, A_i = effect of the basal diet (SD and CD), B_j = effect of the herbal mixture, AB_{ij} = the respective interaction of ith and jth levels of dietary SDCD and herbal additives, and e_{ijk} = random error associated with the ijkth recording. The treatment means were separated by Duncan's multiple-range test at $P \leq 0.05$ statistical level. The effects of the main factors were not considered, whenever the interaction was significant. The non-parametric method of Kruskal-Wallis test was used to analyze liver histopathology scores [29].

Results

Laying performance

Tables 3, 4, 5, and 6 present the effects of dietary interventions on FI, EP, EW, and FCR of laying hens, respectively. Although no significant differences in FI were observed among treatments during the first four weeks of the experiment, both the main effects of the basal diets (SD vs. CD) and the herbal additives became significant in weeks 5 and 6 (Table 3). Hens fed the CD showed reduced FI compared to those on the SD (P = 0.0001). Among the herbal treatments, Mix 2 led to the highest FI during weeks 5 and 6 (P = 0.0053 and P = 0.0003, respectively). From weeks 7 to

12, a significant interaction between dietary treatments and FI was observed (P = 0.0153, P = 0.0015, P = 0.0036, P = 0.0061, P = 0.0034, and P = 0.0031, respectively). During this period, hens receiving the SD, with or without herbal additives, generally showed higher FI. Among the CD groups, CD + Mix 2 resulted in the highest FI and outperformed the other CD-based treatments (Table 3).

EP rate was not significantly affected by the experimental treatments during the first four weeks of the trial (P > 0.05) (Table 4). From week 5 onward, the interaction effects of SD and CD plus herbal mixes on EP were significant (P = 0.0001). Throughout the trial, the CD and CD with herbal mixes showed reduced EP compared to the SD and the SD with herbal mixes. The exception was the CD + Mix 2, which showed no significant difference from the SD group (P > 0.05).

No significant differences were observed in the EW of laying hens during the first four weeks (P > 0.05), except for a significant interaction effect in the first week and a main effect of herbal additives in week 3 (Table 5). In the first week, the group fed with SD had greater EW compared to the SD + Mix 1 (P = 0.0342). In the third week, Mix 2 resulted in a higher EW relative to the control group (P = 0.0445). In addition, the main effects of SD and CD were significant from weeks 5 through 12, with laying hens fed the CD having lower EW than those fed the SD (P = 0.0001). From weeks 5 to 9, the main effects of herbal additives on EW were significant. Diets containing Mix 2 and Mix 3 consistently resulted in higher EW than the control diet (P = 0.0002, P = 0.0002, P = 0.0005, and P = 0.0044, respectively).

FCR was not affected by the experimental treatments between weeks 1 to 4 and weeks 9 to 12 (P > 0.05) (Table 6). The interaction effects of SD and CD with herbal additives were significant during weeks 5 to 8. In week 5, the lowest FCR was observed in the SD, SD + Mix 3, and CD + Mix 2, which were significantly different from CD, CD + Mix 1, and CD + Mix 3 (P = 0.0035). In week

6, the lowest FCR belonged to the CD+ Mix 2 and SD diets, while the highest FCR was related to CD, CD + Mix 4, and CD + Mix 3 (P = 0.0048). In week 7, the CD + Mix 2, SD, SD + Mix 3, and CD groups had the lowest FCR and the CD + Mix 1 and CD + Mix 3 groups had the highest (P = 0.0239). Moreover, CD + Mix 2 and CD + Mix 3 had the lowest and highest FCR, respectively, when measured at week 8 (P = 0.0467).

Table 3. Effect of different combinations of herbal additives with standard and challenge diet on the feed intake of laying hens during weeks 1 to 12 of the trial (65 to 77 weeks of age)

**Treatments**	**Feed intake (g/hen/day)**											
	Week1	Week2	Week3	Week4	Week5	Week6	Week7	Week8	Week9	Week10	Week11	Week12
**Main effects**												
SD	114.56	116.06	111.37	120.97	117.40^{a}	117.84^{a}	117.96^{a}	118.19^{a}	118.53^{a}	118.44^{a}	118.78^{a}	118.84^{a}
CD	114.40	116.40	110.69	118.62	105.34^{b}	103.62^{b}	102.84^{b}	103.31^{b}	96.87^{b}	96.87^{b}	96.87^{b}	96.87^{b}
C	113.44	114.31^{b}	110.75	117.00	110.44^{b}	108.81^{b}	107.19^{c}	107.62^{c}	103.50^{c}	103.31^{c}	104.00^{c}	104.12^{b}
Mix 1	113.31	114.81^{ab}	110.31	119.62	109.50^{b}	109.50^{b}	109.50^{bc}	109.50^{bc}	104.37^{c}	104.37^{bc}	104.37^{bc}	104.37^{b}
Mix 2	115.50	117.75^{a}	112.12	122.81	114.37^{a}	114.06^{a}	114.06^{a}	115.00^{a}	114.12^{a}	114.12^{a}	114.12^{a}	114.12^{a}
Mix 3	115.69	118.06^{a}	110.94	119.75	111.19^{b}	110.56^{b}	110.87^{b}	110.87^{b}	108.81^{b}	108.81^{b}	108.81^{b}	108.81^{b}
**SEM**	12.01	20.13	22.21	55.17	15.20	11.81	17.36	18.07	37.24	39.97	40.07	40.23
**Interactions**												
SD	113.13	112.13	110.88	116.25	115.13	116.88	117.38^{a}	118.25^{a}	117.13^{a}	116.75^{a}	118.13^{a}	118.38^{a}
SD + Mix 1	113.75	116.63	110.38	123.25	116.50	116.50	116.50^{a}	116.50^{a}	116.88^{a}	116.88^{a}	116.88^{a}	116.88^{a}
SD + Mix 2	115.00	116.88	112.38	121.75	119.38	119.38	119.38^{a}	119.38^{a}	120.00^{a}	120.00^{a}	120.00^{a}	120.00^{a}
SD + Mix 3	116.38	118.63	111.88	122.63	118.63	118.63	118.63^{a}	118.63^{a}	120.13^{a}	120.13^{a}	120.13^{a}	120.13^{a}
CD	113.75	116.50	110.63	117.75	105.75	100.75	97.00^{d}	97.00^{d}	89.88^{d}	89.88^{d}	89.88^{d}	89.88^{d}
CD + Mix 1	112.88	113.00	110.25	116.00	102.50	102.50	102.50^{c}	102.50^{c}	91.88^{cd}	91.88^{cd}	91.88^{cd}	91.88^{cd}
CD + Mix 2	116.00	118.63	111.88	123.88	109.38	108.75	108.75^{b}	110.63^{b}	108.25^{b}	108.25^{b}	108.25^{b}	108.25^{b}
CD + Mix 3	115.00	117.50	110.00	116.88	103.75	102.50	103.13^{c}	103.13^{c}	97.50^{c}	97.50^{c}	97.50^{c}	97.50^{c}
**SEM**	3.46	4.49	4.71	7.43	3.90	3.44	4.16	4.25	6.10	6.32	6.33	6.34
**Significant levels**												
SDCD	0.8575	0.7604	0.5619	0.2121	0.0001	0.0001	0.0001	0.0001	0.0001	0.0001	0.0001	0.0001
HA	0.0985	0.0377	0.7306	0.1908	0.0053	0.0003	0.0002	0.0001	0.0001	0.0001	0.0001	0.0001
SDCD*HA	0.7264	0.0780	0.9496	0.1771	0.1202	0.0889	0.0153	0.0015	0.0036	0.0061	0.0034	0.0031

SD=standard diet; CD=challenge diet; C=control (without additive); Mix 1= turmeric, fumitory, green tea and milk thistle powder; Mix 2= lemon, black pepper, sumac and chicory powder; Mix 3= garlic, artichoke, ginger and shallot powder; HA= herbal additive; SDCD*HA= interaction effect of dietary treatments.

a–d Means with no common superscript within each column are significantly ($P \leq 0.05$) different.

SEM= standard error of the mean.

Table 4. Effect of different combinations of herbal additives with standard and challenge diet on the egg production ratio (%) of laying hens during weeks 1 to 12 of the trial (65 to 77 weeks of age)

**Treatments**	Hen-day egg production (%)											
	Week1	Week2	Week3	Week4	Week5	Week6	Week7	Week8	Week9	Week10	Week11	Week12
**Main effects**												
SD	88.37	86.68	85.03	85.81	84.84^{a}	84.37^{a}	83.78^{a}	83.41^{a}	82.09^{a}	81.25^{a}	80.69^{a}	80.40^{a}
CD	88.58	86.86	85.05	85.68	75.59^{b}	75.50^{b}	75.50^{b}	75.53^{b}	71.66^{b}	71.28^{b}	70.34^{b}	70.34^{b}
C	88.44	87.20	85.53	86.42	80.87^{b}	79.87^{b}	79.75^{b}	79.25^{b}	75.12^{bc}	73.19^{c}	71.81^{b}	71.25^{b}
Mix 1	88.67	86.69	85.03	85.61	78.56^{c}	78.56^{b}	78.31^{bc}	78.31^{b}	74.00^{c}	73.50^{c}	72.69^{b}	72.69^{b}
Mix 2	88.00	86.64	84.84	85.64	83.31^{a}	83.25^{a}	82.87^{a}	82.81^{a}	80.69^{a}	80.69^{a}	80.31^{a}	80.31^{a}
Mix 3	88.81	86.55	84.75	85.31	78.12^{c}	78.06^{c}	77.62^{c}	77.50^{b}	77.69^{b}	77.69^{b}	77.25^{a}	77.25^{a}
**SEM**	1.83	1.79	1.63	1.43	5.76	5.87	5.86	6.23	15.89	16.76	20.51	20.89
**Interactions**												
SD	88.62	87.40	85.69	86.87	86.75^{a}	85.12^{a}	84.87^{a}	83.75^{a}	84.75^{a}	81.37^{a}	80.12^{a}	79.00^{a}
SD + Mix 1	88.37	86.12	84.50	85.19	84.00^{b}	84.00^{ab}	83.50^{a}	83.50^{a}	81.12^{a}	81.12^{a}	80.50^{a}	80.50^{a}
SD + Mix 2	87.75	86.31	85.06	85.94	84.25^{ab}	84.12^{ab}	83.37^{a}	83.25^{a}	81.00^{a}	81.00^{a}	80.75^{a}	80.75^{a}
SD + Mix 3	88.75	86.87	84.87	85.26	84.37^{ab}	84.25^{ab}	83.37^{a}	83.12^{a}	81.50^{a}	81.50^{a}	81.37^{a}	81.37^{a}
CD	88.25	87.00	85.37	85.97	75.00^{c}	74.62^{c}	74.62^{b}	74.75^{b}	65.50^{c}	65.00^{c}	63.50^{c}	63.50^{c}
CD + Mix 1	88.97	87.25	85.56	86.04	73.12^{cd}	73.12^{cd}	73.12^{bc}	73.12^{bc}	66.87^{c}	65.87^{c}	64.87^{c}	64.87^{c}
CD + Mix 2	88.25	86.97	84.62	85.34	82.37^{b}	82.37^{b}	82.37^{a}	82.37^{a}	80.37^{a}	80.37^{a}	79.87^{a}	79.87^{a}
CD + Mix 3	88.86	86.22	84.62	85.36	71.87^{d}	71.87^{d}	71.87^{c}	71.87^{c}	73.87^{b}	73.87^{b}	73.12^{b}	73.12^{b}
**SEM**	1.82	1.78	1.63	1.43	5.75	2.42	2.42	2.50	3.99	4.09	4.53	4.57
**Significant levels**												
SDCD	0.5383	0.5835	0.9612	0.6477	0.0001	0.0001	0.0001	0.0001	0.0001	0.0001	0.0001	0.0001
HA	0.3576	0.5193	0.3221	0.0667	0.0001	0.0001	0.0001	0.0001	0.0001	0.0001	0.0001	0.0001
SDCD*HA	0.7334	0.1988	0.3151	0.1774	0.0001	0.0001	0.0001	0.0001	0.0001	0.0001	0.0001	0.0001

SD=standard diet; CD=challenge diet; C=control (without additive); Mix 1= turmeric, fumitory, green tea and milk thistle powder; Mix 2= lemon, black pepper, sumac and chicory powder; Mix 3= garlic, artichoke, ginger and shallot powder; HA= herbal additive; SDCD*HA= interaction effect of dietary treatments.

a–d Means with no common superscript within each column are significantly ($P \leq 0.05$) different.

SEM= standard error of the mean.

Table 5. Effect of different combinations of herbal additives with standard and challenge diet on the egg weight (g) of laying hens during weeks 1 to 12 of the trial (65 to 77 weeks of age)

**Treatments**	Egg weight (g)											
	Week1	Week2	Week3	Week4	Week5	Week6	Week7	Week8	Week9	Week10	Week11	Week12
**Main effects**												
SD	62.56	63.62	63.17	64.69	64.37^{a}	64.37^{a}	64.43^{a}	64.39^{a}	64.51^{a}	64.56^{a}	64.65^{a}	64.75^{a}
CD	62.47	63.58	63.36	65.25	61.75^{b}	61.09^{b}	61.00^{b}	61.09^{b}	61.19^{b}	60.87^{b}	60.81^{b}	61.00^{b}
C	62.56	63.31	62.69^{b}	64.94	62.59^{b}	62.03^{b}	61.91^{b}	61.87^{b}	62.16^{b}	62.16^{b}	62.31	62.50
Mix 1	62.31	63.42	63.37^{ab}	64.20	62.53^{b}	61.84^{b}	61.91^{b}	62.09^{b}	62.14^{b}	62.06^{b}	62.16	62.21
Mix 2	62.62	63.81	63.81^{a}	65.49	63.44^{a}	63.43^{a}	63.44^{a}	63.41^{a}	63.37^{a}	63.06^{ab}	62.94	63.12
Mix 3	62.56	63.87	63.19^{ab}	65.25	63.69^{a}	63.62^{a}	63.62^{a}	63.59^{a}	63.72^{a}	63.59^{a}	63.53	63.69
**SEM**	1.73	1.06	1.21	2.05	1.31	1.81	1.83	1.83	2.20	3.59	4.28	4.21
**Interactions**												
SD	63.25^{a}	63.50	62.50	64.50	63.93	63.93	64.18	64.00	64.06	64.31	64.50	64.87
SD + Mix 1	61.62^{b}	63.12	63.31	64.27	63.69	63.69	63.69	63.68	63.66	63.62	63.68	63.66
SD + Mix 2	62.87^{ab}	63.87	64.00	65.11	64.50	64.50	64.50	64.43	64.87	64.87	64.87	64.87
SD + Mix 3	62.50^{ab}	64.00	62.87	64.87	65.37	65.37	65.37	65.43	65.44	65.44	65.56	65.62
CD	61.87^{ab}	63.12	62.87	65.37	61.25	60.12	59.62	59.75	60.25	60.00	60.12	60.12
CD + Mix 1	63.00^{ab}	63.71	63.44	64.12	61.37	60.00	60.12	60.50	60.62	60.50	60.62	60.75
CD + Mix 2	62.37^{ab}	63.75	63.62	65.87	62.37	62.37	62.37	62.37	61.87	61.25	61.00	61.37
CD + Mix 3	62.62^{ab}	63.75	63.50	65.62	62.00	61.87	61.87	61.75	62.00	61.75	61.50	61.75
**SEM**	1.31	1.03	1.10	1.43	1.14	1.34	1.33	1.35	1.48	1.89	2.07	2.05
**Significant levels**												
SDCD	0.7766	0.8754	0.4983	0.1240	0.0001	0.0001	0.0001	0.0001	0.0001	0.0001	0.0001	0.0001
HA	0.9111	0.3239	0.0445	0.0722	0.0094	0.0002	0.0002	0.0005	0.0044	0.0752	0.2298	0.1865
SDCD*HA	0.0342	0.5568	0.6163	0.7240	0.4319	0.2669	0.0925	0.1424	0.8498	0.8509	0.8302	0.6418

SD=standard diet; CD=challenge diet; C=control (without additive); Mix 1= turmeric, fumitory, green tea and milk thistle powder; Mix 2= lemon, black pepper, sumac and chicory powder; Mix 3= garlic, artichoke, ginger and shallot powder; HA= herbal additive; SDCD*HA= interaction effect of dietary treatments.
a–d Means with no common superscript within each column are significantly ($P \leq 0.05$) different.
SEM= standard error of the mean.

Table 6. Effect of different combinations of herbal additives with standard and challenge diet on the feed conversion ratio of laying hens during weeks 1 to 12 of the trial (65 to 77 weeks of age)

Treatments	Feed conversion ratio (g/g)											
	Week1	Week2	Week3	Week4	Week5	Week6	Week7	Week8	Week9	Week10	Week11	Week12
**Main effects**												
SD	2.07	2.10	2.07	2.17	2.15^{b}	2.17^{b}	2.19	2.20	2.23	2.26	2.27	2.28
CD	2.06	2.11	2.05	2.12	2.26^{a}	2.25^{a}	2.24	2.24	2.22	2.24	2.28	2.28
C	2.051	2.071	2.068	2.081	2.191	2.199^{ab}	2.168^{b}	2.187	2.226	2.277	2.331	2.350
Mix 1	2.054	2.091	2.050	2.175	2.233	2.259^{a}	2.275^{a}	2.262	2.268	2.290	2.312	2.306
Mix 2	2.096	2.130	2.062	2.193	2.164	2.160^{b}	2.168^{b}	2.187	2.229	2.241	2.250	2.250
Mix 3	2.084	2.136	2.075	2.150	2.242	2.232^{ab}	2.262^{ab}	2.262	2.203	2.207	2.225	2.218
**SEM**	0.008	0.011	0.011	0.025	0.013	0.010	0.016	0.015	0.034	0.037	0.044	0.046
**Interactions**												
SD	2.018	2.021	2.087	2.075	2.076^{d}	2.147^{cd}	2.150^{b}	2.200^{abc}	2.158	2.236	2.275	2.312
SD + Mix 1	2.090	2.145	2.050	2.250	2.178^{cd}	2.178^{cd}	2.212^{ab}	2.212^{abc}	2.263	2.264	2.275	2.275
SD + Mix 2	2.085	2.120	2.050	2.175	2.197^{bcd}	2.200^{bcd}	2.225^{ab}	2.225^{abc}	2.283	2.283	2.287	2.287
SD + Mix 3	2.100	2.134	2.100	2.212	2.151^{d}	2.154^{cd}	2.187^{b}	2.187^{bc}	2.253	2.253	2.250	2.250
CD	2.084	2.121	2.050	2.087	2.306^{ab}	2.251^{abc}	2.187^{b}	2.175^{bc}	2.294	2.317	2.387	2.387
CD + Mix 1	2.017	2.038	2.050	2.100	2.287^{abc}	2.341^{a}	2.337^{a}	2.312^{ba}	2.273	2.316	2.350	2.337
CD + Mix 2	2.108	2.140	2.075	2.212	2.132^{d}	2.119^{d}	2.112^{b}	2.150^{c}	2.176	2.199	2.212	2.212
CD + Mix 3	2.067	2.138	2.050	2.087	2.334^{a}	2.310^{ab}	2.337^{a}	2.337^{a}	2.153	2.161	2.200	2.187
**SEM**	0.090	0.106	0.107	0.158	0.115	0.104	0.128	0.125	0.186	0.193	0.210	0.215
**Significant levels**												
SDCD	0.8502	0.8783	0.5623	0.1610	0.0002	0.0019	0.1258	0.2352	0.7386	0.8243	0.7674	1.000
HA	0.4149	0.2614	0.9237	0.2131	0.1978	0.0516	0.0286	0.1368	0.7966	0.6151	0.4384	0.3268
SDCD*HA	0.1536	0.0618	0.7448	0.2418	0.0035	0.0048	0.0239	0.0467	0.2226	0.4632	0.5175	0.6516

SD=standard diet; CD=challenge diet; C=control (without additive); Mix 1= turmeric, fumitory, green tea and milk thistle powder; Mix 2= lemon, black pepper, sumac and chicory powder; Mix 3= garlic, artichoke, ginger and shallot powder; HA= herbal additive; SDCD*HA= interaction effect of dietary treatments.

a–d Means with no common superscript within each column are significantly ($P \leq 0.05$) different.

SEM= standard error of the mean.

Hematological criteria

Tables 7, 8, and 9 show hematological criteria of laying hens fed with herbal additives in both SD and CD at the beginning, middle, and end of the experimental period. Feeding the CD resulted in elevated levels of AST compared to the SD in the middle and at the end of the trial (P = 0.0001). The interaction effects of dietary treatments on AST enzyme levels in the middle and at the end of the trial were significant (P = 0.0001). Specifically, the lowest AST level was observed in laying hens fed with SD + Mix 3 and CD + Mix 2, while the highest levels were associated with the CD and CD + Mix 1 (Table 7).

The interaction effects of CD and SD with herbal additives on the concentration of TP were significant at both the middle and the end of the trial (Table 7). In the middle of the trial, hens fed the CD + Mix 2 had the highest TP concentration, while those on the CD and CD + Mix 1 showed the lowest (P = 0.0428). By the end of the trial, the highest TP concentrations were observed in the CD + Mix 2, SD + Mix 2, and SD + Mix 3 groups, whereas the SD, CD, and CD + Mix 1 groups had the lowest (P = 0.050).

For UA, the interaction effects of experimental treatments were significant at both the middle and the end of the experiment (Table 7). Overall, the SD without additives resulted in the highest blood UA levels. At mid-trial, this was significantly higher than those in the SD + Mix 2, SD + Mix 3, and CD + Mix 2 groups (P = 0.0006). By the end of the trial, the SD group also showed significantly higher UA concentrations compared to SD + Mix 1, SD + Mix 2, SD + Mix 3, and CD + Mix 2 (P = 0.0004). The lowest UA concentrations were consistently observed in the SD + Mix 2 and SD + Mix 3 groups.

The main effects of SD and CD on TG concentration in the second sampling and their interaction effect in the third sampling were significant (Table 8). Based on the results, feeding CD to laying hens increased TG concentration when compared to the SD (*P* = 0.0001). Similarly, in the third sampling,

birds fed with CD, CD + Mix 1, and CD + Mix 3 had the highest amount of TG, and SD + Mix 1 had the lowest (P = 0.0001).

In the second and third samplings, the interaction effects of dietary treatments on TC concentration were significant (Table 8). The highest amount of TC in the second sampling was observed in layers fed with CD + Mix 3, and the lowest in the diet of laying hens fed with SD + Mix 3 (P = 0.0001). In the third sampling, the highest concentration of TC resulted from feeding CD + Mix 1 and CD + Mix 3, and the lowest was observed by feeding SD + Mix 3 (P = 0.0001).

The effect of treatments on HDL concentration exhibited a statistically significant interaction at the second and third samplings (P = 0.0122 and P = 0.0028, respectively). The SD + Mix 3 group had the highest HDL concentration compared to the other treatments (Table 8). As for LDL, the main effect of the SD and CD was significant in the second sampling, with CD increased the concentration of LDL compared to the SD (P = 0.0001) (Table 8).

The interaction effects of diets showed a significant effect on blood Hb concentration and heterophile and lymphocyte count in both the second and third samplings (Table 9). In the second sampling, CD + Mix 2 group had the highest Hb concentration, while the lowest values were observed in SD + Mix 2 and SD + Mix 3 (P = 0.0001). In the third sampling, the highest Hb levels were recorded in the CD and CD + Mix 1 groups, while SD + Mix 2 and SD + Mix 3 showed the lowest concentrations (P = 0.0002). Regarding the number of heterophils in the second and third samplings, the highest count was seen in the SD + Mix 1 group, while the lowest was found in SD + Mix 2 and SD + Mix 3 (P = 0.0001). In the second sampling, the highest lymphocyte counts were recorded in the SD, SD + Mix 1, and all CD groups, whereas the lowest was observed in SD + Mix 2 (P = 0.0108). In the third sampling, CD + Mix 1 exhibited the highest lymphocyte count, while SD + Mix 2 showed the lowest (P = 0.0030).

Table 7. Effect of different combinations of herbal additives with standard and challenge diet on the blood parameters of laying hens (Start, Middle, End)

Treatments	AST(U/L) Start (wk 65)	AST(U/L) Middle (wk 70)	AST(U/L) End (wk 77)	TP(G/dL) Start (wk 65)	TP(G/dL) Middle (wk 70)	TP(G/dL) End (wk 77)	UA(mg/dl) Start (wk 65)	UA(mg/dl) Middle (wk 70)	UA(mg/dl) End (wk 77)
**Main effects**									
SD	213.57	224.78^{b}	232.97^{b}	6.41	6.79^{a}	6.90^{a}	6.18	6.19^{b}	6.56^{b}
CD	213.55	259.84^{a}	295.50^{a}	6.19	6.24^{b}	6.44^{b}	6.30	6.53^{a}	7.28^{a}
C	214.13	253.62^{a}	288.56^{a}	6.21	5.97^{b}	5.99^{c}	6.19	6.79^{a}	7.56^{a}
Mix 1	212.78	253.31^{a}	279.31^{a}	6.35	6.42^{b}	6.53^{b}	6.19	6.71^{a}	7.14^{b}
Mix 2	214.59	220.31^{b}	232.06^{c}	6.28	7.28^{a}	7.40^{a}	6.26	5.91^{b}	6.33^{c}
Mix 3	212.75	242.00^{a}	257.00^{b}	6.37	6.40^{b}	6.77^{b}	6.32	6.03^{b}	6.65^{c}
**SEM**	70.06	469.77	531.16	0.279	0.559	0.430	0.122	0.262	0.332
**Interactions**									
SD	214.25	234.75^{b}	253.87^{b}	6.37	6.35^{bcd}	6.12^{c}	6.12	6.89^{a}	7.69^{a}
SD + Mix 1	209.46	232.87^{b}	235.87^{bc}	6.70	7.02^{ab}	7.06^{ab}	6.17	6.80^{a}	6.91^{b}
SD + Mix 2	218.94	225.62^{bc}	239.37^{bc}	6.46	7.12^{ab}	7.30^{a}	6.31	5.62^{c}	5.72^{c}
SD + Mix 3	211.65	205.87^{c}	202.75^{d}	6.12	6.68^{abc}	7.12^{a}	6.13	5.44^{c}	5.94^{c}
CD	214.01	272.50^{a}	323.25^{a}	6.05	5.60^{d}	5.85^{c}	6.26	6.70^{ab}	7.43^{ab}
CD + Mix 1	216.10	273.75^{a}	322.75^{a}	6.00	5.81^{d}	6.00^{c}	6.21	6.62^{ab}	7.37^{ab}
CD + Mix 2	210.23	215.00^{bc}	224.75^{cd}	6.10	7.43^{a}	7.50^{a}	6.21	6.19^{b}	6.94^{b}
CD + Mix 3	213.86	278.12^{a}	311.25^{a}	6.61	6.12^{cd}	6.41^{bc}	6.52	6.62^{ab}	7.37^{ab}
**SEM**	8.37	21.67	23.05	0.528	0.747	0.656	0.350	0.513	0.576
**Significant levels**									
SDCD	0.9917	0.0001	0.0001	0.0943	0.0046	0.0065	0.1853	0.0090	0.0001
HA	0.8956	0.0001	0.0001	0.8325	0.0001	0.0001	0.6854	0.0001	0.0001
SDCD*HA	0.0799	0.0001	0.0001	0.0870	0.0428	0.0500	0.2647	0.0006	0.0004

SD=standard diet; CD=challenge diet; C=control (without additive); Mix 1= turmeric, fumitory, green tea and milk thistle powder; Mix 2= lemon, black pepper, sumac and chicory powder; Mix 3= garlic, artichoke, ginger and shallot powder; HA= herbal additive; SDCD*HA= interaction effect of dietary treatments; AST= aspartate aminotransferase; TP= total protein; UA= uric acid.

a–d Means with no common superscript within each column are significantly ($P \leq 0.05$) different.

SEM= standard error of the mean.

Table 8. Effect of different combinations of herbal additives with standard and challenge diet on the blood parameters of laying hens (Start, Middle, End)

**Treatments**	**TG (mg/dL)**			**TC (mg/dL)**			**HDL(mg/dl)**			**LDL(mg/dl)**		
	Start (wk 65)	Middle (wk 70)	End (wk 77)	Start (wk 65)	Middle (wk 70)	End (wk 77)	Start (wk 65)	Middle (wk 70)	End (wk 77)	Start (wk 65)	Middle (wk 70)	End (wk 77)
**Main effects**												
SD	1304.28	1410.63^{b}	1485.47^{b}	210.29	199.70^{b}	200.00^{b}	5.39	6.35	6.61	46.42	52.03^{b}	55.88
CD	1434.28	1580.88^{a}	2611.97^{a}	214.59	239.71^{a}	275.19^{a}	5.71	6.51	6.25	46.63	54.44^{a}	56.21
C	1382.19	1503.31	2257.8^{a}	213.64	230.12^{a}	255.42^{a}	5.07	5.70^{b}	5.30^{c}	46.15^{b}	52.88	57.10
Mix 1	1373.44	1473.75	2195.8^{a}	215.12	233.00^{a}	258.62^{a}	5.41	5.91^{b}	5.91^{bc}	46.29^{ab}	53.88	55.59
Mix 2	1388.25	1518.75	1682.9^{b}	209.71	221.47^{a}	230.75^{b}	5.87	6.59^{ab}	6.58^{b}	47.11^{a}	53.41	56.25
Mix 3	1333.00	1487.19	2058.5^{a}	211.30	194.25^{b}	205.56^{c}	5.85	7.53^{a}	7.93^{a}	46.56^{ab}	52.78	55.25
**SEM**	127.64	272.29	120.5	108.43	423.49	531.16	2.43	2.12	1.60	1.38	3.31	9.01
**Interactions**												
SD	1305.50	1462.50	1518.87^{bc}	211.00	225.12^{b}	235.87^{c}	5.01	5.15^{b}	5.11^{c}	45.90	52.50	58.37
SD + Mix 1	1327.12	1371.87	1397.75^{c}	211.50	231.62^{ab}	233.37^{c}	4.63	5.32^{b}	5.56^{bc}	46.43	52.12	55.27
SD + Mix 2	1338.37	1453.12	1564.50^{bc}	208.93	204.19^{c}	203.37^{d}	5.60	6.43^{b}	6.60^{b}	47.15	51.93	55.75
SD + Mix 3	1245.62	1355.00	1460.75^{bc}	209.75	137.87^{d}	127.37^{e}	6.32	8.50^{a}	9.18^{a}	46.22	51.56	54.12
CD	1458.87	1544.12	2996.62^{a}	216.27	235.12^{ab}	275.00^{ab}	5.14	6.25^{b}	5.49^{bc}	46.41	53.26	55.83
CD + Mix 1	1419.75	1575.62	2993.75^{a}	218.75	234.37^{ab}	283.87^{a}	6.19	6.50^{b}	6.26^{bc}	46.13	55.63	55.90
CD + Mix 2	1438.12	1584.37	1801.25^{b}	210.49	238.75^{ab}	258.12^{b}	6.15	6.75^{b}	6.56^{b}	47.07	54.87	56.75
CD + Mix 3	1420.37	1619.37	2656.25^{a}	212.86	250.62^{a}	283.75^{a}	5.37	6.56^{b}	6.68^{b}	46.90	54.00	56.37
**SEM**	113.00	165.20	346.79	10.41	20.58	16.86	1.56	1.45	1.26	1.17	1.82	3.00
**Significant levels**												
SDCD	0.0823	0.0001	0.0001	0.1044	0.0001	0.0001	0.4174	0.6572	0.2548	0.4916	0.0001	0.6576
HA	0.5119	0.8797	0.0001	0.4703	0.0001	0.0001	0.4163	0.0033	0.0001	0.1122	0.2993	0.3221
SDCD*HA	0.6787	0.4248	0.0001	0.8758	0.0001	0.0001	0.1651	0.0122	0.0028	0.5998	0.1785	0.1501

SD=standard diet; CD=challenge diet; C=control (without additive); Mix 1= turmeric, fumitory, green tea and milk thistle powder; Mix 2= lemon, black pepper, sumac and chicory powder; Mix 3= garlic, artichoke, ginger and shallot powder; HA= herbal additive; SDCD*HA= interaction effect of dietary treatments; TG=triglyceride; TC=total cholesterol; HDL=high density lipoprotein; LDL=Low density lipoprotein.

a–d Means with no common superscript within each column are significantly ($P \leq 0.05$) different.

SEM= standard error of the mean.

Table 9. Effect of different combinations of herbal additives with standard and challenge diet on the blood parameters of laying hens (Start, Middle, End)

**Treatments**		**Hb(g/dl)**			**Heterophile**			**Lymphocyte**	
	Start (wk 65)	Middle (wk 70)	End (wk 77)	Start (wk 65)	Middle (wk 70)	End (wk 77)	Start (wk 65)	Middle (wk 70)	End (wk 77)
**Main effects**									
SD	19.41	19.72^{b}	20.69^{b}	25.84	26.54	27.77	73.03	79.95^{b}	82.36^{b}
CD	18.67	24.09^{a}	28.59^{a}	25.63	26.40	27.76	72.83	83.23^{a}	90.09^{a}
C	19.00	23.27^{a}	27.42^{a}	25.41	27.03^{b}	29.37^{a}	72.70	84.09^{a}	90.62^{a}
Mix 1	18.94	23.18^{a}	27.34^{a}	26.05	28.50^{a}	29.94^{a}	72.53	83.77^{ab}	91.65^{a}
Mix 2	17.61	21.27^{b}	21.31^{b}	25.64	25.25^{c}	26.24^{b}	72.93	77.58^{c}	77.62^{c}
Mix 3	20.61	19.91^{b}	22.50^{b}	25.83	25.12^{c}	25.50^{b}	73.56	80.94^{b}	85.00^{b}
**SEM**	6.054	4.99	10.507	0.718	2.133	5.16	1.69	16.60	22.89
**Interactions**									
SD	21.00	23.56^{b}	25.60^{bc}	25.65	27.56^{b}	30.44^{ab}	73.00	84.37^{a}	88.12^{b}
SD + Mix 1	18.61	22.74^{b}	25.17^{c}	26.11	30.50^{a}	31.51^{a}	73.25	83.54^{a}	89.81^{ab}
SD + Mix 2	17.30	16.28^{c}	15.74^{d}	25.92	24.50^{c}	24.22^{d}	72.77	73.41^{c}	74.37^{d}
SD + Mix 3	20.75	16.31^{c}	16.25^{d}	25.67	23.62^{c}	24.87^{d}	73.09	78.50^{b}	77.12^{cd}
CD	17.01	22.99^{b}	29.25^{a}	25.17	26.50^{b}	28.31^{bc}	72.40	83.81^{a}	93.12^{ab}
CD + Mix 1	19.26	23.62^{b}	29.50^{a}	25.99	26.50^{b}	28.37^{bc}	71.81	84.00^{a}	93.50^{a}
CD + Mix 2	17.92	26.25^{a}	26.87^{abc}	25.36	26.00^{b}	28.25^{bc}	73.09	81.75^{ab}	80.87^{c}
CD + Mix 3	20.47	23.50^{b}	28.75^{ab}	25.99	26.62^{b}	26.12^{cd}	74.02	83.37^{a}	92.87^{ab}
**SEM**	2.46	2.21	3.24	0.847	1.46	2.27	1.30	4.07	4.78
**Significant levels**									
SDCD	0.2298	0.0001	0.0001	0.3201	0.7016	0.9956	0.5429	0.0021	0.0001
HA	0.0721	0.0001	0.0001	0.1904	0.0001	0.0001	0.1391	0.0001	0.0001
SDCD*HA	0.0923	0.0001	0.0002	0.4563	0.0001	0.0001	0.0632	0.0108	0.0030

SD=standard diet; CD=challenge diet; C=control (without additive); Mix 1= turmeric, fumitory, green tea and milk thistle powder; Mix 2= lemon, black pepper, sumac and chicory powder; Mix 3= garlic, artichoke, ginger and shallot powder; HA= herbal additive; SDCD*HA= interaction effect of dietary treatments; Hb=hemoglobin.

a–d Means with no common superscript within each column are significantly ($P \leq 0.05$) different.

SEM= standard error of the mean.

Hepatic histopathology

Fig 3 illustrates the effects of various herbal mixes on the frequency of liver histopathology scores in laying hens. Statistical analysis revealed a significant difference ($P = 0.002$) among the treatment groups. Notably, hens fed the SD + Mix 2 and CD + Mix 2 exhibited significantly lower liver scores compared to those fed CD without additive. No significant differences were observed among the other experimental treatments ($P > 0.05$).

Fig 3. The Effect of different combinations of herbal additives with standard and challenge diet on the liver histopathology score of laying hens. SD, standard diet; CD, challenge diet; Mix 1= turmeric, fumitory, green tea and milk thistle powder; Mix 2= lemon, black pepper, sumac and chicory powder; Mix 3= garlic, artichoke, ginger and shallot powder. Different letters indicate significant difference among different treatments at the 0.05 level

Discussion

This study aimed to investigate the effects of various medicinal plant mixtures on laying hen performance, blood chemistry, and liver health. These mixtures were administered alongside either a SD or a CD. Among the tested combinations, hens receiving a CD supplemented with Mix 2 (comprising lemon, black pepper, sumac, and chicory) demonstrated significant improvements in EP and FCR, while also mitigating adverse effects on liver health.

The chemical composition of these herbal mixtures can exhibit variability depending on the plant species, growth conditions, and extraction methods employed. The GC-MS analysis of the herbal essential oils revealed that a substantial portion of the identified compounds in each oil were phthalates. Phthalides, a class of secondary metabolites, possess a broad spectrum of

pharmacological activities, including anti-inflammatory and antimicrobial activities [30]. Furthermore, most essential oils contained terpenes, a well-studied group recognized for their anti-inflammatory and antioxidant effects [31]. Examples identified in this study included caryophyllene, beta-bisabolene, limonene, and α-copaene, among others. Additionally, the presence of organosulfur compounds, another class of potentially beneficial natural products, was observed in garlic and shallot [16]. Notably, curcumin, a curcuminoid with established health benefits, constituted the predominant component in turmeric essential oil [19].

In this study, the CD was formulated with high energy and low protein content to induce FLHS [32]. Biologically, the high energy intake relative to protein causes an imbalance in macronutrient metabolism, favoring lipogenesis over protein synthesis [33]. This results in excessive fat deposition, particularly in the liver, which impairs liver function and overall metabolic homeostasis [33].

Feeding the CD significantly decreased FI, EW, and EP rate from week 5 onward, with a concomitant increase in FCR compared to feeding a SD. These declines in productive performance can be logically attributed to altered satiety signaling and metabolic stress [34]. Specifically, the accumulation of lipids in the liver and adipose tissue leads to increased leptin secretion—a hormone known to suppress appetite—thereby reducing FI [32, 35]. Reduced nutrient intake then contributes to a decline in EP and EW, as laying hens prioritize survival over reproduction under physiological stress. Additionally, impaired liver function can reduce the synthesis of essential compounds for egg formation, such as yolk precursors and lipoproteins, further compromising productivity [36]. Similar to these findings, a previous study documented a decline in EW, EP rate, and FI in hens fed a CD [32]. Notably, these reductions began in weeks 2, 3, and 4 for each measure, respectively, and affected both young and old hens [32]. Moreover, another study

reported that hens fed a CD consumed less feed and had a lower EP rate than hens on a control diet [7]. The metabolic consequences of the CD extend beyond FI and egg parameters. High hepatic lipid accumulation is a precursor to FLHS, which not only physically damages the liver but also induces systemic inflammation [37]. Inflammatory responses and oxidative stress disrupt hormonal regulation and blood flow to reproductive organs, further diminishing productivity [4, 7, 37, 38]. In the current study, the CD + Mix 2 largely compensated for the damage caused by the CD, improving FI, EP, and FCR of laying hens. These improvements are logically attributed to the biological activities of the mix's components. The herbs in Mix 2 contain a variety of secondary metabolites—such as flavonoids, phenolic acids, and terpenoids—with proven antioxidant, anti-inflammatory, and lipid-lowering properties. These compounds may inhibit de novo lipogenesis, enhance lipid catabolism, and protect hepatic tissue from oxidative damage, thereby restoring liver function and metabolic balance [11–14, 22, 39]. Two theories have been proposed for the pathogenesis of FLHS. The first theory suggests that FLHS begins with the excessive deposition of TG in the liver, disrupting the homeostasis of lipid metabolism [40]. The second theory proposes that oxidative stress in FLHS induces excessive reactive oxygen species in the liver, leading to the destruction of hepatic connective tissues, called reticulin [40]. By mitigating both lipid accumulation and oxidative stress, Mix 2 likely targets both pathways of FLHS progression, leading to improved liver histopathology and overall hen health. Furthermore, the combined use of lemon, sumac, black pepper, and chicory may exert synergistic effects, enhancing the therapeutic impact beyond what individual herbs could achieve. Limited information is available about the role of medicinal plants in preventing FLHS in laying hens. A previous study found that adding a mixture of chicory, turmeric, artichoke, and milk thistle powder to a high energy/low protein diet did not affect EP or FCR in laying hens [41]. However, artichoke and

thistle powder reduced FI, while turmeric powder increased EW compared to the control group [41]. Another study observed that broiler chickens fed diets containing chicory root powder showed significant improvement in growth performance, likely due to increased food digestion and absorption caused by changes in jejunum histomorphometry [42]. Supplementation with 0.5% sumac in feed was found to slightly increase EW in another study, although FI, FCR, and EP were not significantly affected [43]. Research has shown that adding lemon essential oil to quail feed improved FCR and FI, possibly due to the active compounds and the antioxidant and antimicrobial properties of the oil [44]. A recent study demonstrated that incorporating garlic into laying hen diets could increase EW [45]. Additionally, another study reported increased EP, EW, and FI in groups treated with inulin [46]. Inconsistencies observed across these studies may be attributed to factors such as variations in dosage, limited absorption of the additives, and potential interactions between different herbal plants.

Laying hens fed the CD, with or without herbal additives, exhibited higher blood AST levels compared to those on the SD at both the middle and the end of the experiment. This elevation suggests hepatocellular injury, as AST is a key intracellular enzyme released into circulation when liver cells are damaged or inflamed [47]. The high energy and low protein content of the CD likely promote excessive hepatic lipid accumulation, which disrupts liver architecture, induces oxidative stress, and compromises hepatocyte integrity, thereby increasing AST release into the bloodstream [32, 48]. Notably, the inclusion of Mix 2 in the CD-fed groups mitigated the rise in AST, indicating a potential protective effect of its herbal constituents on liver health. The antioxidant and anti-inflammatory properties of lemon, black pepper, sumac, and chicory—each rich in bioactive phytochemicals such as polyphenols, flavonoids, and terpenes—are likely responsible for reducing oxidative damage and stabilizing hepatocyte membranes [11, 13, 49]. This supports the hypothesis

that phytogenic additives can attenuate liver injury under metabolic stress conditions [22]. These findings align with previous reports indicating that high-energy, low-protein diets are associated with increased AST levels in laying hens, reflecting diet-induced hepatic stress and early stages of fatty liver development [32, 48]. While direct studies on the effects of medicinal plants on FLHS in laying hens are scarce, related research in avian species suggests beneficial outcomes. For instance, lemon essential oil supplementation in quail diets has been shown to reduce AST levels, potentially due to cytoprotective and antioxidative actions [44]. However, results across studies remain inconsistent. Some research found that sumac supplementation had no significant effect on AST concentrations [50, 51], and others reported that combinations of chicory, turmeric, artichoke, and milk thistle did not reduce AST levels when included in a CD for laying hens [41]. In contrast, other studies have demonstrated that herbal blends containing milk thistle significantly lowered AST in hens with fatty liver, highlighting the hepatoprotective role of specific plant constituents [52]. These contrasting results may be attributed to various factors, including the dosage of herbal additives, interactions among different herbs, or the quality of the herbs used.

All herbal mixtures incorporated into the SD increased blood TP concentration compared to the control (SD without additives). This elevation in TP may reflect improved dietary protein utilization and metabolic efficiency, potentially mediated by enhanced gut integrity, nutrient absorption, and systemic antioxidant capacity [53]. Herbal bioactives—such as flavonoids, phenolics, and essential oils—are known to modulate gut microbiota, reduce oxidative damage to enterocytes, and improve intestinal morphology, thereby supporting better nutrient digestion and absorption, including amino acids required for plasma protein synthesis [53]. For example, ginger supplementation at a rate of 5 g/kg of diet has been reported to increase serum TP concentrations in broilers, likely due to an enhanced antioxidant status [54]. Additionally, Oleforuh-Okoleh et al.

[55] demonstrated that adding garlic to broiler diets increased blood TP compared to the control group. The only CD treatment with a TP concentration similar to the SD groups was CD + Mix 2. This suggests that Mix 2 may have improved protein retention or minimized catabolism despite the protein-deficient nature of the CD. A well-established relationship exists between dietary protein levels and plasma protein concentration [56]; therefore, the reduced TP levels in other CD-fed groups might result from impaired protein synthesis, increased protein turnover, or hepatic dysfunction commonly associated with fatty liver development [57]. Supporting this, lemon essential oil has previously been shown to increase blood TP in quail, possibly through its antioxidant properties and positive impact on liver and gut function [44]. However, not all herbal additives exert the same effect. For example, supplementation with chicory, turmeric, artichoke, and milk thistle did not significantly alter TP levels in laying hens on a high-energy, low-protein diet [41], and sumac inclusion at 0.25–0.5% showed no effect on TP concentration in another study [51]. These findings differ from the present study. Notably, the enhanced effect observed with Mix 2 may be due to synergistic interactions among its components—lemon, black pepper, sumac, and chicory. Synergism among herbs occurs when combinations of bioactive compounds interact in ways that amplify their individual physiological effects, such as through complementary antioxidant actions or cooperative effects on gut and liver function [58]. This could explain why Mix 2 was more effective in maintaining TP levels than individual herbs used alone in other studies.

The increased blood UA concentration observed in the CD groups was not mitigated by the addition of herbal supplements. The lowest UA concentrations were observed in the SD + Mix 2 and SD + Mix 3 groups. UA a byproduct of protein catabolism, is primarily eliminated from the body through urinary excretion. Previous research suggests that fatty liver may elevate circulatory

UA levels, and UA has been proposed as an independent predictor of fatty liver risk [59]. In agreement with our results, one study reported that supplementing a high-energy, low-protein diet with chicory, turmeric, artichoke, and thistle powder did not significantly affect UA concentrations in laying hens [41]. Similarly, Mosayyeb Zadeh et al. [51] found that dietary inclusion of sumac 0.25, and 0.5% had no significant effect on UA levels in laying hens.

In the current study, blood TG concentrations significantly increased in hens fed the CD by the second sampling point, reflecting disrupted lipid metabolism and hepatic lipid overload. Excess dietary energy and insufficient protein may impair hepatic lipid handling, causing triglycerides to accumulate in the liver and leak into circulation. However, in the third sampling, the CD + Mix 2 treatment attenuated this increase compared to other CD treatments. This suggests that Mix 2 may enhance lipid metabolism, reduce hepatic fat accumulation, or improve lipid clearance from the bloodstream. The lipid-lowering effect is likely mediated through the antioxidant and fat-modulatory properties of its herbal components. Studies suggest that sumac and its polyphenols may reduce TG levels by hindering fat digestion and absorption [51, 60]. Similarly, an herbal mix containing lemon was reported to reduce TG levels in overweight rats [61]. This effect may be attributed to lemon's vitamin C and flavonoid content, which act as antioxidants [49, 62]. TC concentrations also increased under the influence of the CD in both the second and third samplings, and the herbal additives were unable to restore this effect. Elevated TC is commonly associated with FLHS, as impaired lipid metabolism and liver dysfunction can lead to increased cholesterol synthesis and impaired excretion [6, 48]. The inability of herbal mixtures to reduce TC under CD conditions may be due to insufficient duration of treatment, suboptimal dosing, or complex interactions among the bioactive compounds that diminished their cholesterol-lowering efficacy. The lowest TC concentration was observed in the SD + Mix 3 group which includes garlic and

shallot—members of the *Allium* genus well-documented for their hypocholesterolemic effects. These effects are often attributed to sulfur-containing compounds that influence lipid metabolism and bile acid synthesis [16, 63, 64]. Akbarian et al. [65] showed a decrease in blood TC in laying hens fed ginger root powder (0.25%, 0.5%, and 0.75%) over an 8-week period. One possible mechanism involves the modulation of bile acids [54]. Artichoke, also found in Mix 3, may contribute by enhancing biliary excretion of cholesterol and supporting liver detoxification [66]. In the present study, feeding the CD increased blood LDL concentrations in laying hens compared to the SD during the second sampling. LDL, commonly referred to as "bad cholesterol," is a key contributor to lipid accumulation in blood vessels and a known risk factor for cardiovascular disease [67]. Despite this initial increase, supplementation with herbal additives in both SD and CD groups did not lead to significant changes in LDL levels, suggesting limited impact of the additives on LDL metabolism within the timeframe of the experiment. Overall, no reduction in blood HDL was observed in the CD groups, regardless of herbal additive inclusion, when compared to the SD group. HDL is a type of lipoprotein responsible for transporting cholesterol from peripheral tissues to the liver for excretion. It is often referred to as "good cholesterol" due to its positive impact on reducing the risk of cardiovascular diseases [68]. The ratio of LDL to HDL in the bloodstream is an important indicator of lipid metabolism status [69]. Notably, in contrast to these findings, some studies have reported both increased LDL and decreased HDL in hens fed high-energy, low-protein diets [40]. Although fatty liver in laying hens can negatively impact HDL levels, this effect is not always observed. Several factors—including the severity and duration of fatty liver, dietary composition, nutritional status, and genetic predisposition—can influence HDL levels. In the present study, hens in the SD + Mix 3 group exhibited the highest HDL concentrations during both the second and third samplings compared to the other treatments.

Mix 3 includes garlic, ginger, artichoke, and shallot—herbs that are rich in bioactive compounds like allicin, gingerols, and cynarin, which have been associated with improved lipid profiles through mechanisms such as enhanced bile secretion, reduced hepatic lipogenesis, and increased antioxidant capacity [16, 53]. Previous research has shown that HDL cholesterol levels increased in laying quails fed diets containing 1%, 2%, and 4% garlic powder [70]. Similarly, Saki et al. [71] reported significantly higher serum HDL levels in laying hens fed diets supplemented with 8 or 12 g/kg of an herb mixture. Moreover, an increase in serum HDL cholesterol was reported in all treated groups with ginger rhizome oil in Japanese quails, relative to the control group [72].

The concentration of Hb across different sampling times did not follow a consistent trend. However, overall, Hb levels were elevated in laying hens fed the CD, both with and without herbal additives, compared to those in the SD + Mix 2 and SD + Mix 3 groups. A previous study showed that a high-fat diet increased Hb concentrations in mice compared to the control diet supplemented with herbal additives [61]. It is important to note that the relationship between fatty liver on Hb concentration in laying hens is complex and may be influenced by various factors, including the severity and etiology of fatty liver, as well as metabolic pathways involved in erythropoietin production and red blood cell turnover. Another study reported elevated circulating Hb levels in patients with fatty liver, with the increase correlating with the degree of hepatic steatosis. In such cases, Hb may function as an antioxidant, mitigating some of the negative effects associated with the disease [73].

In the present study, no significant differences were observed in heterophile counts among hens fed the CD with or without herbal additives. However, the SD + Mix 1 group exhibited the highest heterophile counts during the second and third samplings. Lymphocyte numbers increased in the CD groups, while the lowest counts were observed in the SD + Mix 2 and SD + Mix 3 groups.

According to a previous study, a low white blood cell count may suggest either a disease-free condition or suppressed bone marrow activity [74]. It is presumed that the medicinal plants included in Mix 2 and Mix 3 possess anti-inflammatory, antimicrobial, and immune system-stimulating properties. The potential alleviation of fatty liver symptoms may have contributed to the observed reduction in lymphocyte numbers. In support of this, one study found that dietary supplementation with 25 and 50 mg/kg garlic in broilers had no significant effect on lymphocyte counts, though the higher dose (50 mg/kg) significantly reduced heterophile counts compared to the control [75]. In contrast to our results, another study reported that garlic increased heterophile, lymphocyte, and the heterophile to lymphocyte ratio, and attributed these responses to a stimulatory effect of garlic on the immune system [76]. In our study, the CD alone led to a higher lymphocyte count than CD + Mix 2, suggesting that the combination of herbal plants in Mix 2 may have attenuated the stress associated with the CD. It is also important to consider that factors such as age, breed, and environmental conditions may influence heterophile levels, making it difficult to predict the effects of fatty liver on heterophile counts in laying hens. Supporting this complexity, one study showed that feeding a diet containing lemon and garlic resulted in a reduced white blood cell count compared to a high-fat control diet [61].

Lower liver histopathology scores were observed in hens fed the CD supplemented with Mix 2 compared to those without additives, suggesting a potential protective role of the herbs in Mix 2 against FLHS development. Although the precise mechanisms underlying this protective effect remain unclear, the individual components of the herbal blend may contribute to FLHS prevention through their known biological activities. For example, lemon and black pepper are recognized for their antioxidant and anti-inflammatory effects [12, 13, 77], which may help mitigate oxidative stress and inflammation—key factors in FLHS pathogenesis. Sumac, reported to regulate lipid

metabolism [11], could play a role in preventing excessive fat accumulation in the liver. Additionally, dietary chicory has been associated with improved overall health and reduced liver histological changes [78], possibly due to its content of isoflavones, polyphenols, and antioxidants that may help reduce liver injury. However, existing data on the impact of medicinal plants on liver pathology are mixed. While some studies have found no significant effect of herbal essential oil mixtures on liver parameters [41, 79], others have demonstrated the efficacy of specific herbal mixtures in reducing liver fat accumulation and inflammation in laying hens [52]. These discrepancies underscore the variability in biological responses to different herbal treatments. Trott et al. [4] noted varying degrees of hepatocellular vacuolization in FLHS-affected chickens, indicating that not all birds experience severe hepatic fat buildup. Similarly, Song et al. [33] reported that the severity of liver lesions may vary depending on factors such as the hens' age and dietary composition. These findings highlight the complexity of FLHS and the need for further research to better understand the individual and synergistic effects of these herbs on liver health and the progression of FLHS.

Conclusion

The present experiment demonstrated that hens fed the CD exhibited reduced FI, EW, and EP rate, along with elevated blood levels of AST (indicating liver damage), and UA (indicating protein catabolism). Additionally, their livers showed a higher histopathology scores consistent with FLHS. Among the herbal mixtures tested, Mix 2—comprising sumac, lemon, black pepper, and chicory—was the most effective. It improved performance parameters (FI, EW, and EP rate), lowered AST levels, and reduced liver histopathology scores in hens fed the CD. In contrast, the effects of other herbal mixtures were less consistent; some improved select parameters, while others showed no significant impact. Overall, the findings suggest that certain medicinal plant

mixtures, particularly Mix 2, have the potential to alleviate the adverse effects of FLHS in laying hens. However, we have acknowledged key limitations of the study, including the relatively short duration, the use of a single dose per herbal mixture, the lack of individual herb evaluation, and the restriction to one hen strain and age group, which may limit the broader applicability of the findings.

Declarations

Consent for publication

Not applicable.

Availability of data and materials

All data generated or analysed during this study are included in this published article [and its supplementary information files].

Competing interests

The authors declare that they have no competing interests.

Funding

No funding was received to assist with the preparation of this manuscript.

Authors' contributions

Mohammadreza Khodaei conducted the experiment, gathered the data, created the result tables, and authored the initial version of the manuscript. Mehran Torki was responsible for the research design and oversight, as well as for revising and submitting the draft manuscript. Fariborz Khajali conducted the statistical analysis of the experimental data and revised the manuscript. Iraj Karimi

conducted a histopathological examination of the liver. All authors reviewed and endorsed the final manuscript.

Acknowledgements

The authors appreciate Razi University for supporting the current study.

References

1. Aydin, R. 2005. Type of fatty acids, lipoprotein secretion from liver and fatty liver syndrome in laying hens. Int J Poult Sci. 2005;4:917-919.

2. Butler EJ. Lipid metabolism in the fowl under normal and abnormal circumstances. Proc Nutr Soc. 1975;34:29-34. https://doi.org/10.1079/PNS19750007

3. Butler EJ. Fatty liver diseases in the domestic fowl—A review. Avian Pathol. 1976;5:1-4. https://doi.org/10.1080/03079457608418164

4. Trott KA, Giannitti F, Rimoldi G, Hill A, Woods L, Barr B, et al. Fatty liver hemorrhagic syndrome in the backyard chicken: a retrospective histopathologic case series. Vet Pathol. 2014;51:787-95. https://doi.org/10.1177/03009858135035

5. Shini A, Shini S, Bryden WL. Fatty liver haemorrhagic syndrome occurrence in laying hens: impact of production system. Avian Pathol. 2019;48:25-34. https://doi.org/10.1080/03079457.2018.1538550

6. Choi YI, Ahn HJ, Lee BK, Oh ST, An BK, Kang CW. Nutritional and hormonal induction of fatty liver syndrome and effects of dietary lipotropic factors in egg-type male chicks. Asian-Australas J Anim Sci. 2012;25:1145. https://doi.org/10.5713/ajas.2011.11418

7. Jiang S, Cheng HW, Cui LY, Zhou ZL, Hou JF. Changes of blood parameters associated with bone remodeling following experimentally induced fatty liver disorder in laying hens. Poult Sci. 2013;92:1443-53. https://doi.org/10.3382/ps.2012-02800

8. Squires EJ, Leeson S. Aetiology of fatty liver syndrome in laying hens. Br Vet J. 1988;144:602-609. https://doi.org/10.1016/0007-1935(88)90031-0

9. Pearson AW, Butler EJ, Curtis RF, Fenwick GR, Hobson-Frohock A, Land DG, et al. Effects of rapeseed meal on laying hens (*Gallus domesticus*) in relation to fatty liver-haemorrhagic syndrome and egg taint. Res Vet Sci. 1978;25:307-13. https://doi.org/10.1016/S0034-5288(18)32945-X

10. Couch JR. Fatty livers in laying hens—a condition which may occur as a result of increased strain. Feedstuffs. 1956;28(47):46.

11. Shariatmadari F, Shariatmadari R. Sumac (*Rhus coriaria*) supplementation in poultry diet. World's Poult Sci. J 2020;76:358-64. https://doi.org/10.1080/00439339.2020.1745724

12. Manners GD. Citrus limonoids: analysis, bioactivity, and biomedical prospects. J Agric Food Chem. 2007;55:8285-94. https://doi.org/10.1021/jf071797h

13. Zuzanna Bober Z, Agnieszka Stępień A, David Aebisher D, Łukasz Ożog Ł, Dorota Bartusik-Aebisher D. Medicinal benefits from the use of Black pepper, Curcuma and Ginger. Eur J Clin Exp Med. 2018;133-45. 10.15584/ejcem.2018.2.9

14. Nandagopal S, Kumari BR. Phytochemical and antibacterial studies of Chicory (*Cichorium intybus L.*)-A multipurpose medicinal plant. Adv Biol Res. 2007;1:17-21.

15. Khazaei R, Seidavi A, Bouyeh M. A review on the mechanisms of the effect of silymarin in milk thistle (*Silybum marianum*) on some laboratory animals. Vet Med Sci. 2022;8:289-301. https://doi.org/10.1002/vms3.641

16. Kothari D, Lee WD, Niu KM, Kim SK. The genus Allium as poultry feed additive: A review. Animals. 2019;9:1032. https://doi.org/10.3390/ani9121032

17. Abadjieva D, Grigorova S, Mladenova V, Shimkus A, Kistanova E. Effect of artichoke (*Cynara scolimus L.*) on the egg productivity and biochemical parameters in laying hens. Bulg J Agric Sci. 2020; 26: 1280-5.

18. Chung MY, Park HJ, Manautou JE, Koo SI, Bruno RS. Green tea extract protects against nonalcoholic steatohepatitis in ob/ob mice by decreasing oxidative and nitrative stress responses induced by proinflammatory enzymes. J Nutr Biochem. 2012;23:361-7. https://doi.org/10.1016/j.jnutbio.2011.01.001

19. Kermanshahi H, Riasi A. Effect of turmeric rhizome powder (*Curcuma longa*) and soluble NSP degrading enzyme on some blood parameters of laying hens. Int J Poult Sci. 2006;5:494-8.

20. Sharma UR, Prakash T, Surendra V, Roopakarki N, Rao NR, Divakar G. Hepatoprotective activity of Fumaria officinalis against CCl4-induced liver damage in rats. Pharmacology. 2012;3:9-14.

21. Khan RU, Naz S, Nikousefat Z, Tufarelli VI, Javdani M, Qureshi MS, et al. Potential applications of ginger (*Zingiber officinale*) in poultry diets. World's Poult Sci J. 2012;68:245-52. https://doi.org/10.1017/S004393391200030X

22. Li XL, He WL, Wang ZB, Xu TS. Effects of Chinese herbal mixture on performance, egg quality and blood biochemical parameters of laying hens. J Anim Physiol Anim Nutr. 2016;100:1041-9. https://doi.org/10.1111/jpn.12473

23. Lohmann Breeders. 2020. LSL-Lite Layers-Management Guide. Lohmann, Cuxhaven, Germany. https://lohmann-breeders.com/media/strains/cage/management/LOHMANN-LSL-Lite-Cage-1.pdf

24. Marai IF, Habeeb AA, Gad AE. Rabbits' productive, reproductive and physiological performance traits as affected by heat stress: a review. Livest Prod Sci. 2002;78(2):71-90. https://doi.org/10.1016/S0301-6226(02)00091-X

25. Naghneh NS, Rafiei F, Shahbazi E, Gheisari MM. Variation of volatile compounds in celery through different vegetative stages. Plant Physiol. 2023;28:476-80. https://doi.org/10.1007/s40502-023-00744-6

26. Anene DO, Akter Y, Thomson PC, Groves P, O'Shea CJ. Variation and association of hen performance and egg quality traits in individual early-laying ISA Brown Hens. Animals. 2020;10(9):1601. https://doi.org/10.3390/ani10091601

27. Kianfar R, Di Rosa AR, Divari N, Janmohammadi H, Hosseintabar-Ghasemabad B, Oteri M, et al. A comparison of the effects of raw and processed amaranth grain on laying hens' performance, egg physicochemical properties, blood biochemistry and egg fatty acids. Animals. 2023;13(8):1394.

28. Cherian G, Hayat Z. Long-term effects of feeding flaxseeds on hepatic lipid characteristics and histopathology of laying hens. Poult Sci. 2009;88:2555-61. https://doi.org/10.3382/ps.2009-00425

29. SAS (Statistical Analyses System). 2015. SAS user's guide. Version 9.4. Cary, NC: SAS Inst.

30. Karmakar R, Pahari P, Mal D. Phthalides and Phthalans: Synthetic methodologies and their applications in the total synthesis. Chem Rev. 2014;114: 6213–84. https://doi.org/10.1021/cr400524q

31. Moses T, Pollier J, Thevelein J M, Goossens A. Bioengineering of plant (tri) terpenoids: From metabolic engineering of plants to synthetic biology in vivo and in vitro. New Phytologist. 2013;200: 27–43. https://doi.org/10.1111/nph.12325

32. Rozenboim I, Mahato J, Cohen NA, Tirosh O. Low protein and high-energy diet: a possible natural cause of fatty liver hemorrhagic syndrome in caged White Leghorn laying hens. Poult Sci. 2016;95:612-21. https://doi.org/10.3382/ps/pev367

33. Song Y, Ruan J, Luo J, Wang T, Yang F, Cao H, et al. Abnormal histopathology, fat percent and hepatic apolipoprotein AI and apolipoprotein B100 mRNA expression in fatty liver

hemorrhagic syndrome and their improvement by soybean lecithin. Poult Sci. 2017;96:3559-63. https://doi.org/10.3382/ps/pex163

34. McNab JM, Boorman KN. Poultry feedstuffs: supply, composition and nutritive value. CABI publishing. 2002. https://doi.org/10.1079/9780851994642.0000

35. Richards MP. Genetic regulation of feed intake and energy balance in poultry. Poult Sci. 2003;82:907-16. https://doi.org/10.1093/ps/82.6.907

36. Zaefarian F, Abdollahi MR, Cowieson A, Ravindran V. Avian liver: the forgotten organ. Animals. 2019;9(2):63. https://doi.org/10.3390/ani9020063

37. Shini S, Bryden WL. Occurrence and control of fatty liver haemorrhagic syndrome (FLHS) in caged hens. A report for the Australian Egg Corporation Limited AECL Publication No uq-105a AECL Project No UQ-105. 2009;1448-316.

38. Lee BK, Kim JS, Ahn HJ, Hwang JH, Kim JM, Lee HT, et al. Changes in hepatic lipid parameters and hepatic messenger ribonucleic acid expression following estradiol administration in laying hens (*Gallus domesticus*). Poult Sci. 2010;89:2660-7. https://doi.org/10.3382/ps.2010-00686

39. Panyod S, Sheen LY. Beneficial effects of Chinese herbs in the treatment of fatty liver diseases. J Tradit Complement Med. 2020;10:260-7. https://doi.org/10.1016/j.jtcme.2020.02.008

40. Miao YF, Gao XN, Xu DN, Li MC, Gao ZS, Tang ZH, et al. Protective effect of the new prepared Atractylodes macrocephala Koidz polysaccharide on fatty liver hemorrhagic syndrome in laying hens. Poult Sci. 2021;100:938-48. https://doi.org/10.1016/j.psj.2020.11.036

41. Assi Husain H, Sadeghi A, Karimi A. Effects of chicory, turmeric, artichoke and sage powder in high energy and low protein diets on yield, egg quality and fatty liver status in laying hens. Anim Sci. 2023;36:19-38. 10.22092/ASJ.2022.358220.2215

42. Izadi H, Arshami J, Golian A, Raji MR. Effects of chicory root powder on growth performance and histomorphometry of jejunum in broiler chicks. Vet Res Forum. 2013;4:169-174.

43. Arpášová H, Kačániová M, Gálik B, Čuboň J, Mellen M. The effect of oregano essential oil and Rhus coriaria L. on selected performance parameters of laying hens. Scientific Papers Anim Sci Biotech. 2014;47:12-12.

44. Alagawany M, El-Saadony MT, Elnesr SS, Farahat M, Attia G, Madkour M, et al. Use of lemongrass essential oil as a feed additive in quail's nutrition: its effect on growth, carcass, blood biochemistry, antioxidant and immunological indices, digestive enzymes and intestinal microbiota. Poult Sci. 2021;100:101172. https://doi.org/10.1016/j.psj.2021.101172

45. Omer HA, Ahmed SM, Abdel-Magid SS, El-Mallah GM, Bakr AA, Abdel Fattah MM. Nutritional impact of inclusion of garlic (Allium sativum) and/or onion (Allium cepa L.) powder in laying hens' diets on their performance, egg quality, and some blood constituents. Bull Natl Res Cent. 2019;43:1-9. https://doi.org/10.1186/s42269-019-0061-6

46. Park SO, Park BS. Effect of feeding inulin oligosaccharides on cecum bacteria, egg quality and egg production in laying hens. Afric J Biotech. 2012;11:9516-21. https://doi.org/10.5897/AJB12.5250

47. Yousefi M, Shivazad M, Sohrabi-Haghdoost I. Effect of dietary factors on induction of fatty liver-hemorrhagic syndrome and its diagnosis methods with use of serum and liver parameters in laying hens. Int J Poult Sci. 2005;4:468-72.

48. Zhang J, Chen D, Yu B. Effect of different dietary energy sources on induction of fatty liver-hemorrhagic syndrome in laying hens. Int J Poult Sci. 2008;7:1232-6.

49. González-Molina E, Domínguez-Perles R, Moreno DA, García-Viguera C. Natural bioactive compounds of Citrus limon for food and health. J Pharm Biomed Anal. 2010;51:327-45. https://doi.org/10.1016/j.jpba.2009.07.027

50. Gumus H, Oguz MN, Bugdayci KE, Oguz FK. Effects of sumac and turmeric as feed additives on performance, egg quality traits, and blood parameters of laying hens. Rev Bras de Zootecn. 2018;47. https://doi.org/10.1590/rbz4720170114

51. Mosayyeb Zadeh A, Mirghelenj SA, Shakouri Alishah H, Hasanlou P. Effects of sumac fruit powder (Rhus coriaria L.) supplementation on productive performance, egg quality traits and serum biochemical parameters in old laying hens. Ital J Anim Sci. 2021;20:1348-56. https://doi.org/10.1080/1828051X.2021.1965922

52. Zhu Y, Zhang X, Du P, Wang Z, Luo P, Huang Y, et al. Dietary herbaceous mixture supplementation reduced hepatic lipid deposition and improved hepatic health status in post-peak laying hens. Poult Sci. 2022;101:101870. https://doi.org/10.1016/j.psj.2022.101870

53. Abdelli N, Solà-Oriol D, Pérez JF. Phytogenic feed additives in poultry: achievements, prospective and challenges. Animals. 2021;11(12):3471. https://doi.org/10.3390/ani11123471

54. Zhang GF, Yang ZB, Wang Y, Yang WR, Jiang SZ, Gai GS. Effects of ginger root (Zingiber officinale) processed to different particle sizes on growth performance, antioxidant status, and serum metabolites of broiler chickens. Poult Sci. 2009;88:2159-66. https://doi.org/10.3382/ps.2009-00165

55. Oleforuh-Okoleh VU, Ndofor-Foleng HM, Olorunleke SO, Uguru JO. Evaluation of growth performance, haematological and serum biochemical response of broiler chickens to aqueous extract of ginger and garlic. J Agric Sci. 2015;7:167. https://doi.org/10.5539/jas.v7n4p167

56. Bush BM. Interpretation of laboratory results for small animal clinicians. Blackwell Scientific Publications Ltd. 1991;225-9.

57. Du X, Wang Y, Amevor FK, Ning Z, Deng X, Wu Y, et al. Effect of high energy low protein diet on lipid metabolism and inflammation in the liver and abdominal adipose tissue of laying hens. Animals. 2024;14(8):1199. https://doi.org/10.3390/ani14081199

58. Kiyohara H, Matsumoto T, Yamada H. Combination effects of herbs in a multi-herbal formula: expression of Juzen-taiho-to's immuno-modulatory activity on the intestinal immune system. J Evid Based Complementary Altern Med. 2004;1:83-91. https://doi.org/10.1093/ecam/neh004

59. Oral A, Sahin T, Turker F, Kocak E. Relationship between serum uric acid levels and nonalcoholic fatty liver disease in non-obese patients. Med. 2019;55:600. https://doi.org/10.3390/medicina55090600

60. Shishikura Y, Khokhar S, Murray BS. Effects of tea polyphenols on emulsification of olive oil in a small intestine model system. J Agric Food Chem. 2006;54:1906-13. https://doi.org/10.1021/jf051988p

61. Bassuony NI, Hafez LM, Ibrahim AF, El-Sebeay AS, Omer HA. Effect of using natural bioactive mixture composed of lemon, onion and garlic juices on weight and lipid profiles for overweight rats. Eur J Appl Sci. 2021;13:09-17.

62. Prabuseenivasan S, Jayakumar M, Ignacimuthu S. In vitro antibacterial activity of some plant essential oils. BMC Complement Altern Med. 2006;6:1-8. https://doi.org/10.1186/1472-6882-6-39

63. Chowdhury SR, Chowdhury SD, Smith TK. Effects of dietary garlic on cholesterol metabolism in laying hens. Poult Sci. 2002;81:1856-62. https://doi.org/10.1093/ps/81.12.1856

64. Mahmoud KZ, Gharaibeh SM, Zakaria HA, Qatramiz AM. Garlic (*Allium sativum*) supplementation: Influence on egg production, quality, and yolk cholesterol level in layer hens. Asian-Australas J Anim Sci. 2010;23:1503-9. https://doi.org/10.5713/ajas.2010.10124

65. Akbarian A, Golian A, Sheikh Ahmadi A, Moravej H. Effects of ginger root (*Zingiber officinale*) on egg yolk cholesterol, antioxidant status and performance of laying hens. J Appl Anim Res. 2011;39:19-21. https://doi.org/10.1080/09712119.2011.558612

66. Qiang Z, Lee SO, Ye Z, Wu X, Hendrich S. Artichoke extract lowered plasma cholesterol and increased fecal bile acids in Golden Syrian hamsters. Phytother Res. 2012;26:1048-52. https://doi.org/10.1002/ptr.3698

67. Ridker PM. LDL cholesterol: controversies and future therapeutic directions. Lancet. 2014;384:607-17. https://doi.org/10.1016/S0140-6736(14)61009-6

68. Hermansen K, Dinesen B, Hoie LH, Morgenstern E, Gruenwald J. Effects of soy and other natural products on LDL: HDL ratio and other lipid parameters: a literature review. Adv Ther. 2003;20:50-78. https://doi.org/10.1007/BF02850119

69. Zhang X, Han Z, Zhong H, Yin Q, Xiao J, Wang F, et al. Regulation of triglyceride synthesis by estradiol in the livers of hybrid tilapia (Oreochromis niloticus♀× O. aureus♂). Comparative Biochemistry and Physiology Part B: Biochem Mol Biol. 2019;238:110335. https://doi.org/10.1016/j.cbpb.2019.110335

70. Canogullari S, Baylan M, Erdogan Z, Duzguner V, Kucukgul A. The effects of dietary garlic powder on performance, egg yolk and serum cholesterol concentrations in laying quails. Czech J Anim Sci. 2010;55:286-93. https://doi.org/10.17221/126/2009-CJAS

71. Saki AA, Aliarabi H, Siyar SA, Salari J, Hashemi M. Effect of a phytogenic feed additive on performance, ovarian morphology, serum lipid parameters and egg sensory quality in laying hen. Vet Res Forum. 2014;5:287–293.

72. Herve T, Raphaël KJ, Ferdinand N, Victor Herman N, Willy Marvel NM, Cyril D’Alex T, et al. Effects of ginger (*Zingiber officinale, Roscoe*) essential oil on growth and laying performances, serum metabolites, and egg yolk antioxidant and cholesterol status in laying Japanese quail. J Vet Med. 2019;2019. https://doi.org/10.1155/2019/7857504

73. Juárez-Hernández E, Chávez-Tapia NC, Brizuela-Alcántara DC, Uribe M, Ramos-Ostos MH, Nuño-Lámbarri N. Association between serum hemoglobin levels and non alcoholic fatty liver disease in a mexican population. Ann Hepatol. 2018;17:577-84. https://doi.org/10.5604/01.3001.0012.0920

74. Medugu CI, Kwari ID, Igwebuike J, Nkama I, Mohammed ID, Hamaker B. Carcass and blood components of broiler chickens fed sorghum or millet as replacement for maize in the semi arid zone of Nigeria. Agric Biol J North Am. 2010;1:326-9.

75. El-katcha MI, Soltan MA, Sharaf MM, Hasen A. Growth performance, immune response, blood serum parameters, nutrient digestibility and carcass traits of broiler chicken as affected by dietary supplementation of garlic extract (allicin). Alex J Vet Sci. 2016 15;49. https://doi.org/10.5455/ajvs.219261

76. Eid KM, Iraqi MM. Effect of garlic powder on growth performance and immune response for newcastle and avian influenza virus diseases in broiler of chickens. Anim Biotechnol. 2014;1:7-13.

77. Li Y, Yang M, Lin H, Yan W, Deng G, Ye H, et al. Limonin alleviates non-alcoholic fatty liver disease by reducing lipid accumulation, suppressing inflammation and oxidative stress. Front Pharmacol. 2022;12:801730.

78. Jamzadfard M, Ebrahimi H. Herbal Medicine in the Treatment of Fatty Liver Disease: Evaluation of the therapeutic Effects of some herbs that constitute Magic Liver herbal distillate.

79. Mousavi A, Mahdavi AH, Riasi A, Soltani-Ghombavani MJ. Synergetic effects of essential oils mixture improved egg quality traits, oxidative stability and liver health indices in laying hens fed fish oil. Anim Feed Sci Technol. 2017;234:162-72.

80. Amiri HR, Mohammadi M, Sadatmand S, Taheri E. Study the chemical composition of essential oil of ginger (*zingiber officinale*) and antioxidant and cell toxicity. J Med Plants. 2016 ;15:89-98.

81. Majdi B, Mehrnia MA, Barzegar H, Alizadeh Behbahani B. Determination of the structure, chemical composition, antioxidant activity and the cytotoxic effect of Turmeric essential oil. Iran Food Sci Technol Res J. 2021;17(2):261-71. https://doi.org/10.22067/ifstrj.2020.39272

82. Khoshtinat K, Barzegar M, Sahari MA, Hamidi Z. Encapsulation of Iranian Garlic Oil with β-cyclodextrin: Optimization and its Characterization. J Agric Sci Technol. 2017;19: 97-111.

83. Morshed S, Hossain MD, Ahmad M, Junayed M. Physicochemical characteristics of essential oil of black pepper (*Piper nigrum*) cultivated in Chittagong, Bangladesh. J. Food Qual Hazards Control. 2017;4(3):66-9.

Figure

Click here to access/download;Figure;Fig 1.jpg

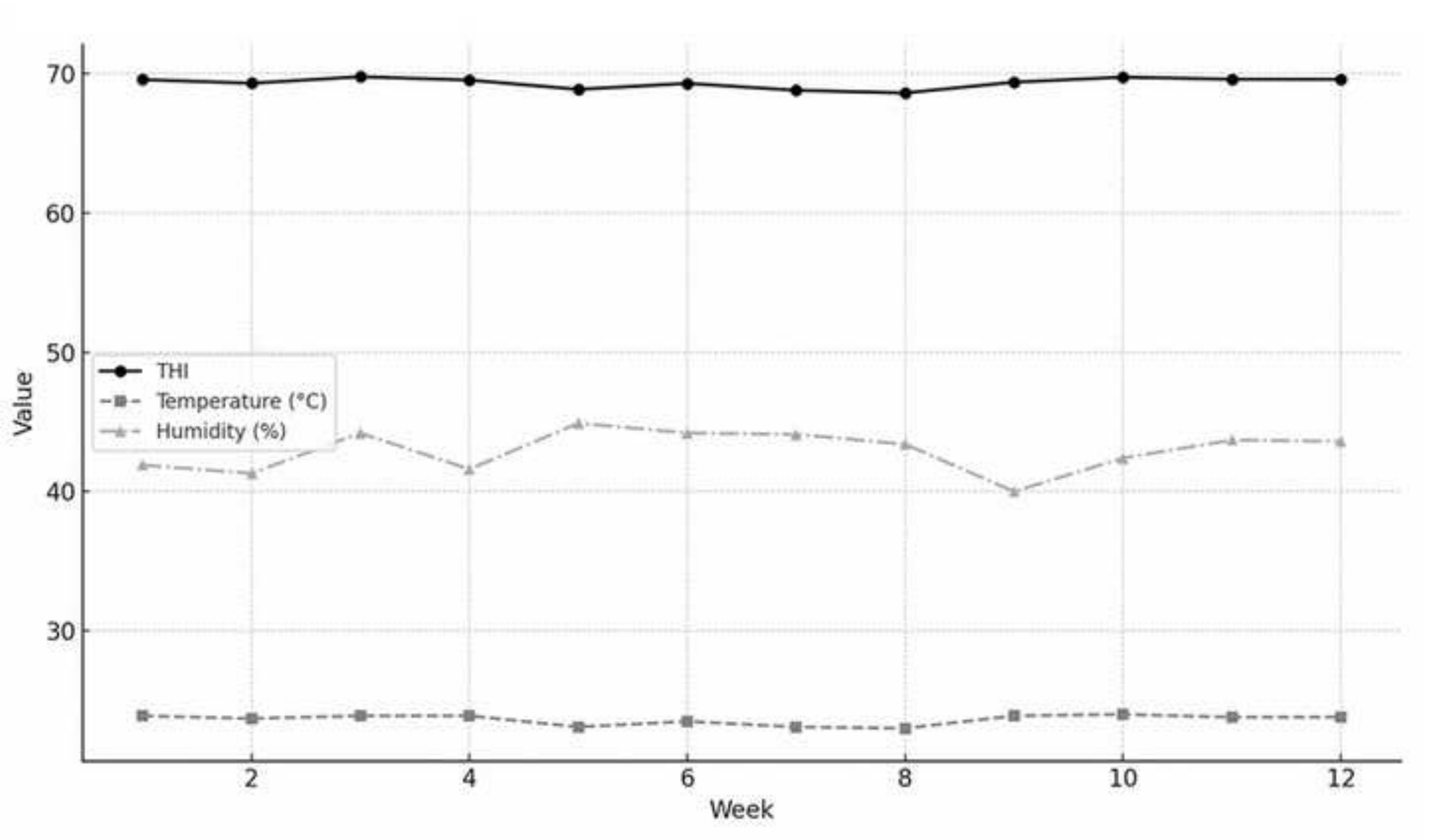

Figure

Click here to access/download;Figure;Fig 2 B.jpg

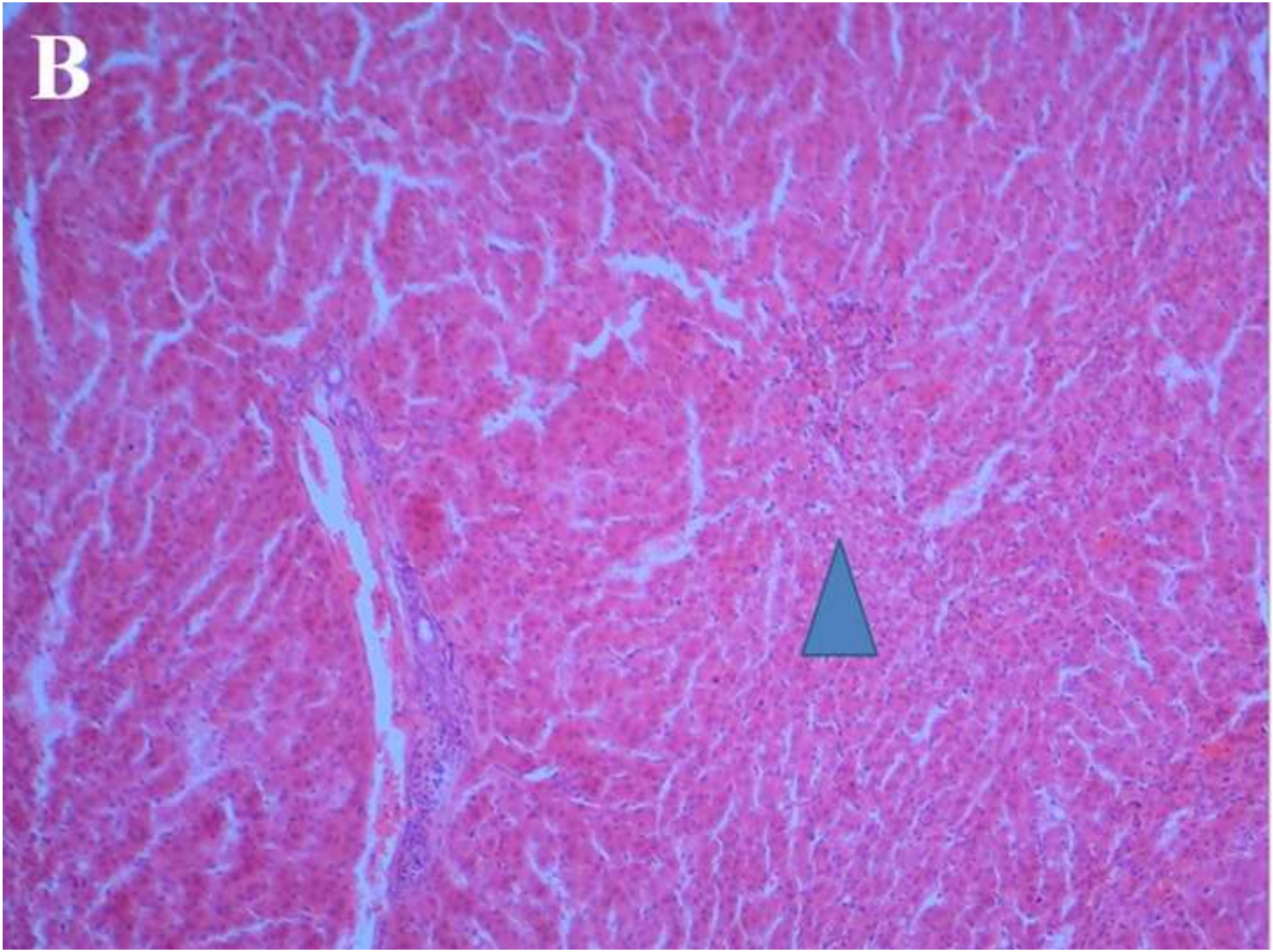

Figure

Click here to access/download;Figure;Fig 2 C.jpg

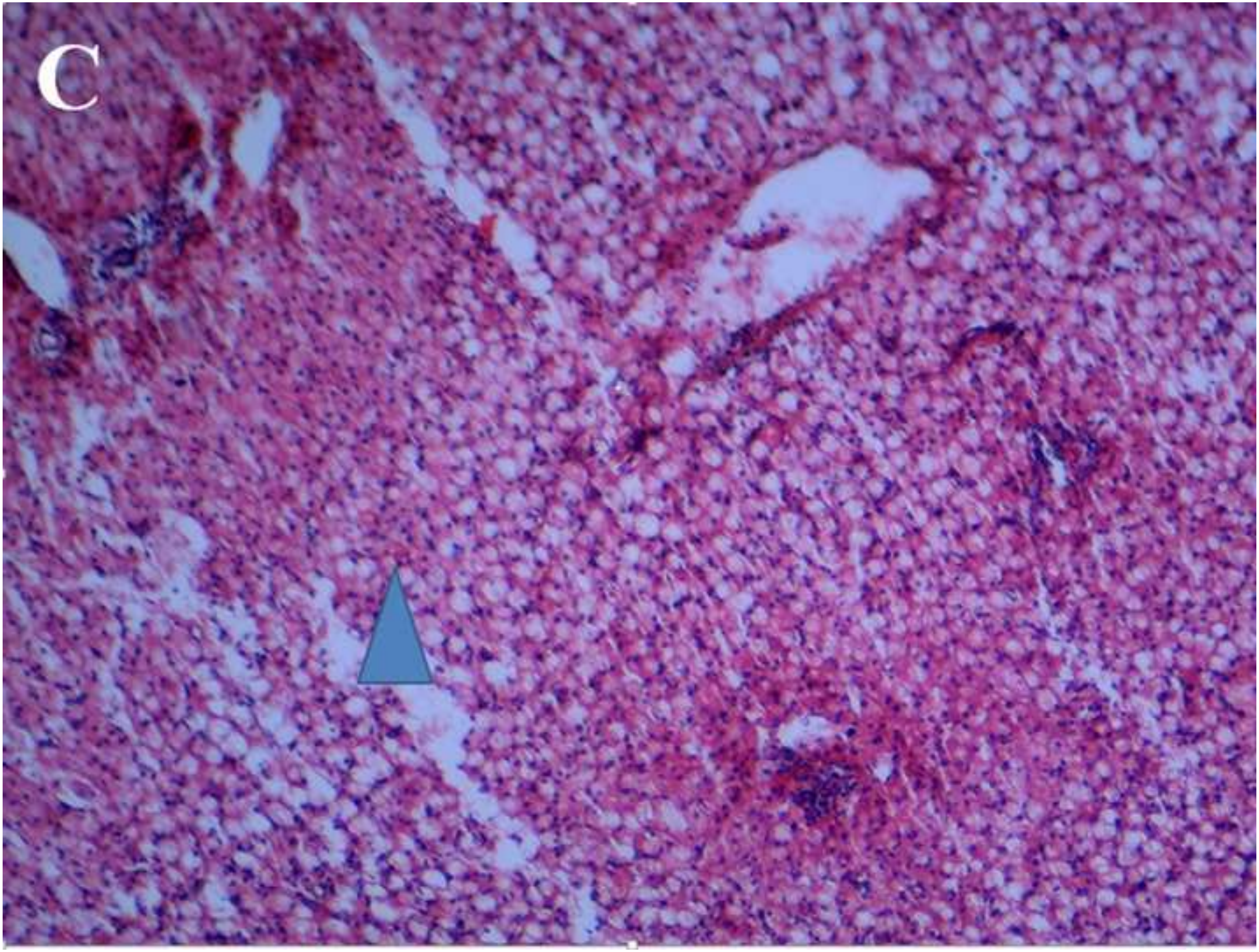

Figure

Click here to access/download;Figure;Fig 2 D.jpg

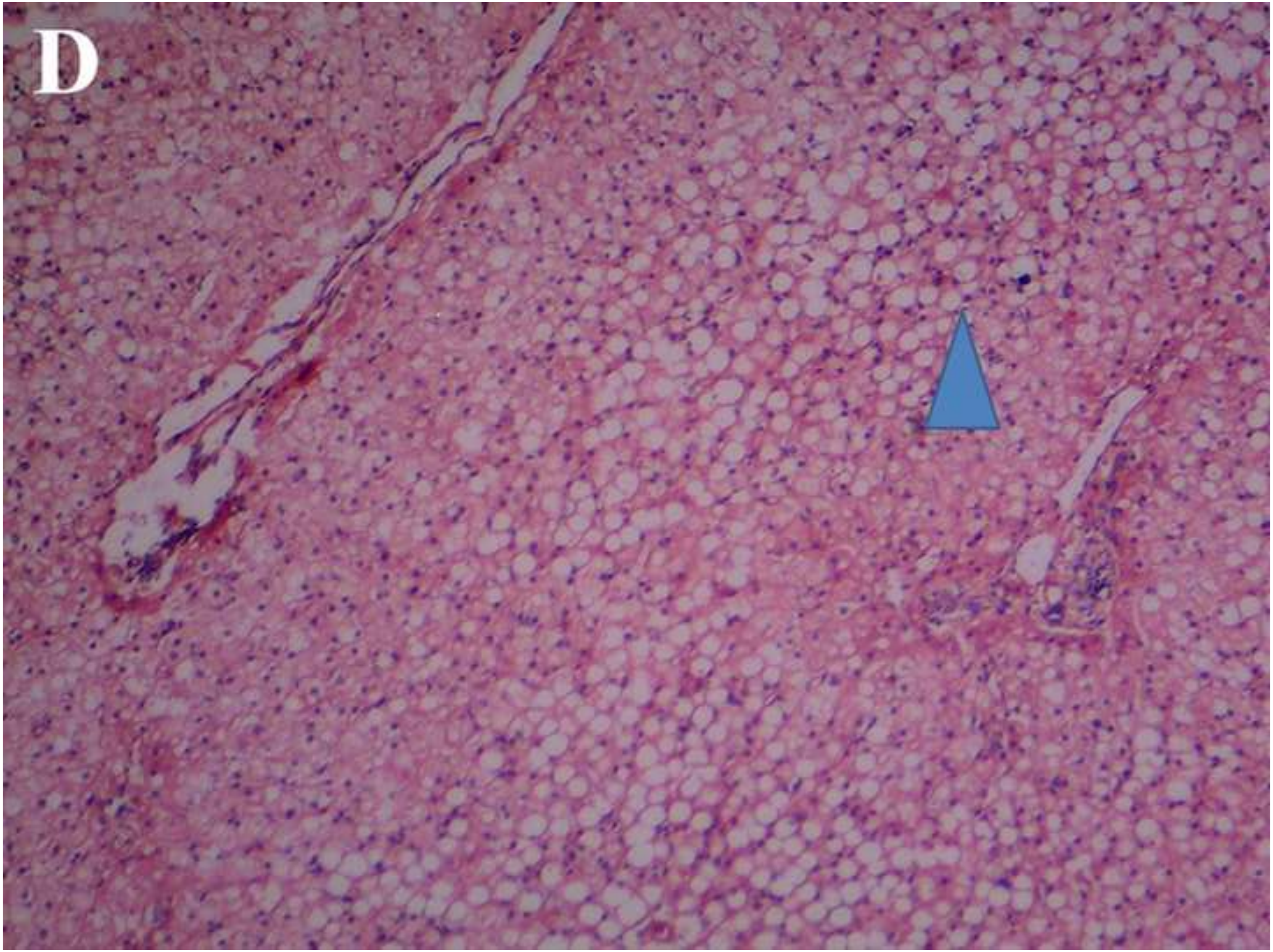

Click here to access/download;Figure;Fig 3.jpg

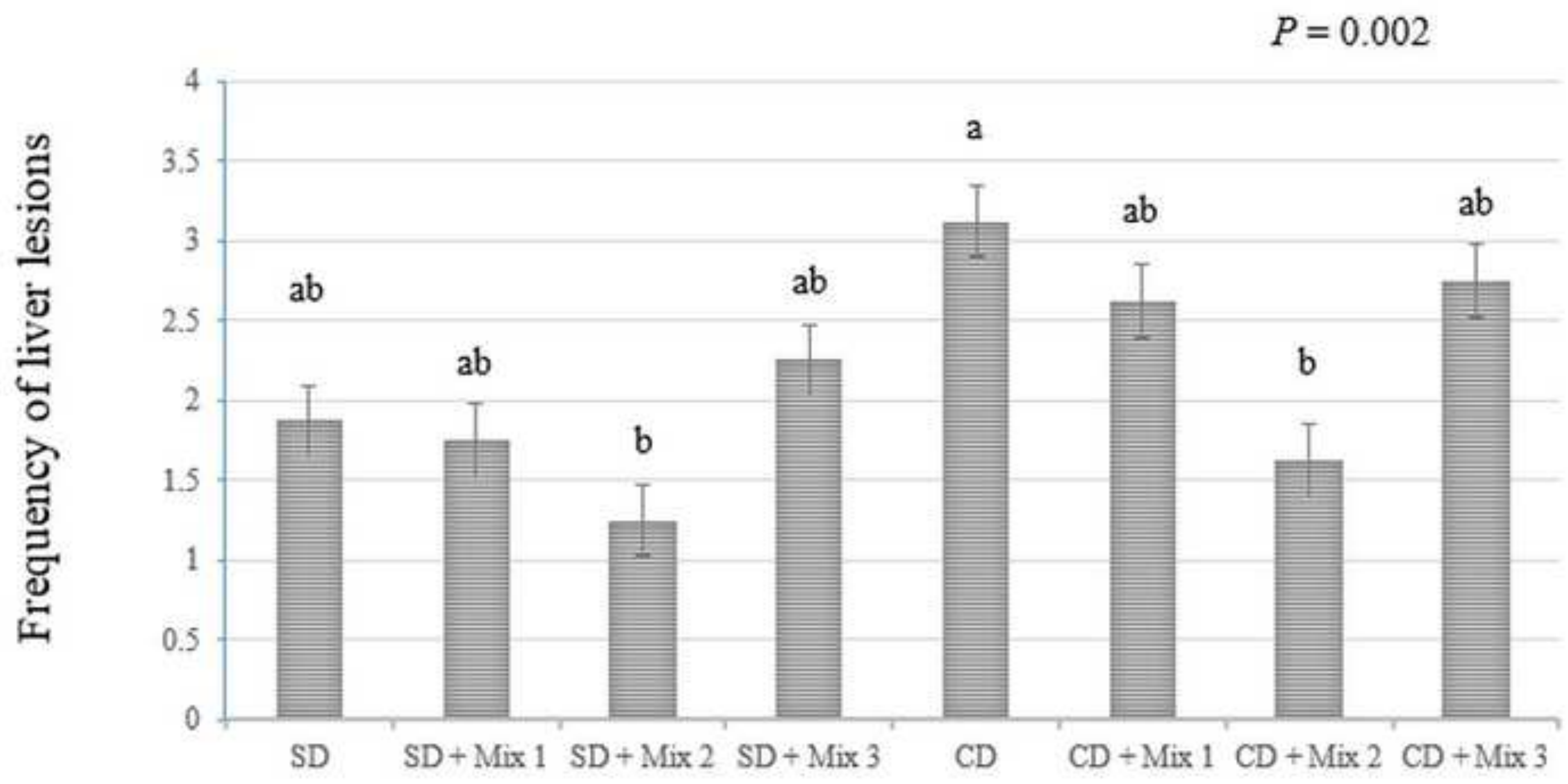

Figure

Click here to access/download;Figure;Fig 2 A.jpg

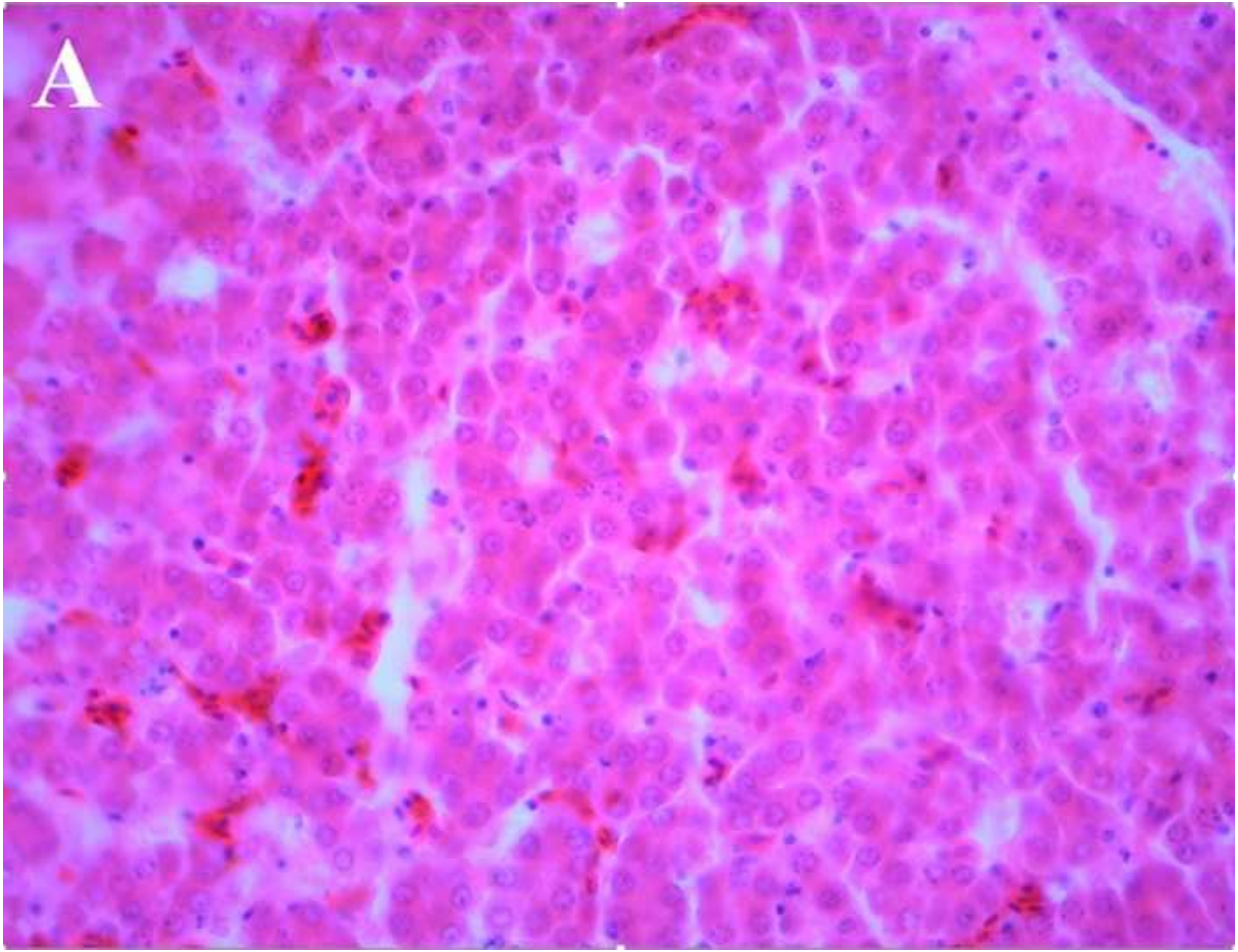

Click here to access/download
Supporting Information
Additional file 1.docx

Application of ~~V~~various mixtures of medicinal herbs in the diet of laying hens: Evaluating preventive approach of fatty liver syndrome

Mohammadreza Khodaei[1], Mehran Torki[1*], Fariborz Khajali[2], Iraj Karimi[3]

[1] Department of Animal Science, College of Agriculture and Natural Resources, Razi University, Kermanshah, Iran, Postal Code: 67156-85423

[2] Department of Animal Science, Shahrekord University, Shahrekord, Iran, Postal Code: 88186-34141

[3] Department of Pathology, Faculty of Veterinary Medicine, Shahrekord University, Shahrekord, Iran

[*] Corresponding author: torki@razi.ac.ir

Running title: Dietary medicinal herbs to prevent fatty liver syndrome in laying hens

Abstract

Fatty liver hemorrhagic syndrome (FLHS) is a major health issue in laying hens, which is associated with reduced productive performance and increased mortality. This study investigated the efficacy of three ~~distinct~~ herbal additive mixes as dietary interventions to prevent FLHS in a total of 384 LSL-Lohmann laying hens ~~aged~~from 65 to 77 weeks of age.~~Herbal additives have been explored as a dietary intervention to prevent the occurrence of FLHS. In the present study, the effect of three herbal mixes have been examined in preventing FLHS in laying hens. The study~~

~~involved 384 LSL-Lohmann laying hens from the age of 65 to 77 weeks.~~ Hens were allocated to ~~an 8-~~ eight treatments ~~,~~in a 2×4 factorial design, comprising two basal diets—a standard diet (SD) and a high-energy, low-protein challenge diet (CD)—alongside four dietary interventions (three herbal mixes and a control). The herbal mixes were formulated as follows: Mix 1 (turmeric, fumitory, green tea, milk thistle), Mix 2 (lemon, black pepper, sumac, chicory), and Mix 3 (garlic, artichoke, ginger, shallot). Productive performance, blood variables, and liver lesion scores were ~~meticulous~~carefully assessed.~~Hens were randomly distributed among 8 treatments, each consisted of 8 replicates (cages) of six birds in a 2 × 4 factorial layout. There were two basal diets including a standard diet (SD; having commercial norms of energy and protein) and a challenge diet (CD; high-energy and low-protein diet) as well as four dietary interventions (three mixes of herbs and a control with no herbal additives). Mix 1 consisted of turmeric, fumitory, green tea, and milk thistle. Mix 2 included lemon, black pepper, sumac, and chicory. Mix 3 contained garlic, artichoke, ginger, and shallot. Productive performance, blood variables and liver lesion scores were examined.~~ The results demonstrated that the CD decreased feed intake (FI; $P = 0.0001$), egg production (EP; $P = 0.0001$), and egg weight (EW; $P = 0.0001$) from week 5 onward.~~,~~ Birds received the CD had poorer feed conversion ratio (FCR) as opposed to the SD in weeks 5 and 6 of the trial. ~~whereas it increased feed conversion ratio (FCR) from Week 5 onward.~~ Feeding the CD resulted in higher circulatory levels of aspartate aminotransferase (AST; $P = 0.0001$) and triglycerides (TG; $P = 0.0001$), and a higher frequency of severe livers scores ($P = 0.002$). Notably, Mix 2 emerged as the most effective intervention, significantly reversing the negative impacts of the CD across all measured parameters.~~Inclusion of Mix 2 significantly restores these responses.~~ However, the herbal mixes did not significantly affect heterophil and lymphocyte counts or hemoglobin (Hb) concentration when fed with the CD. ~~Inclusion of herbal mixes while feeding the CD did not make a significant~~

~~effect on the number of heterophiles and lymphocytes and hemoglobin (Hb) concentration.~~ Overall, the CD significantly impaired productive performance and increased liver pathological lesions. However, supplementing the CD with Mix 2 (lemon, black pepper, sumac, and chicory) consistently led to the most significant improvements across all measured parameters, proving to be an effective dietary intervention to prevent FLHS in laying hens.

~~feeding a CD could result in poor productive performance and it was associated with a higher frequency of liver pathological lesions. Supplementing the CD with Mix 2 resulted in the most noticeable improvements in all parameters measured. The combination of lemon, black pepper, sumac, and chicory was found to be effective dietary intervention to prevent FLHS in laying hens.~~

Keywords: Egg Production; Fatty Liver; Laying Hen; Medicinal Herb

~~Background~~ Introduction

A typical layer diet provides approximately 2-4 g of lipids per day, which is considerably less than the amount required for body weight gain and egg formation [1]. In birds, lipids are primarily synthesized in the liver. As a result, there is a heavy burden on the liver to form necessary lipids. Consequently, dealing with an enormous amount of lipids predisposes laying hens to fatty liver hemorrhagic syndrome (FLHS) [2, 3].

~~In birds, lipids are primarily synthesized in the liver. A typical layer diet contains only 2-4% lipids, while body weight gain and egg formation require much higher lipids to be formed [1]. The liver accounts for supplying lipids for these vital functions. Consequently, the liver of laying hens is naturally dealt with an enormous amount of lipids [2, 3]. This situation predisposes laying hens to fatty liver hemorrhagic syndrome (FLHS).~~

FLHS is a non-infectious disease characterized by the excessive accumulation of fat in the liver and abdominal cavity. This significant fat buildup can lead to hepatocyte rupture, internal bleeding, and sudden death in affected birds [4, 5]excessive fat build-up in the liver and abdominal cavitThis fat accumulation can lead to the rupture of hepatocytes, internal bleeding, and sudden death in birds [4, 5]. FLHS is more commonly observed in laying hens that are housed in cage systems [5, 6].The syndrome is more commonly observed in laying hens housed in cage systems [5, 6]. Affected flocks suffer substantial economic losses due to decreased egg production (EP) and increased mortality rates [4, 7]Flocks affected by FLHS experience significant economic losses due to decreased egg production and increased mortality rates [4, 7]. During necropsy, birds that died from FLHS exhibit enlarged, soft, yellow, and fatty livers. This condition makes the liver more susceptible to tearing and bleeding [3, 5]. This compromised state makes the liver more susceptible to tearing and bleeding [3, 5].

The exact cause of FLHS remains unclear, but several factors are thought to be involvedcontribute. including These include nutritional factors like (excess dietary energy, fungal toxins, and anti-nutritional substances) [2, 8, 9], as well as genetic predisposition, and environmental factors (such as ambient temperature and rearing system) [5, 10]. It is likely that a combination of these factors may contribute to the development of FLHS.

Given the multifactorial nature of FLHS, various nutritional strategies have been explored to mitigate its impact—particularly those aimed at reducing hepatic lipid synthesis [6].Several nutritional strategies have been investigated to reduce hepatic lipid biosynthesis and prevent FLHS. Among these, medicinal plants have emerged as a promising avenueapproach due to their bioactive compounds with health-promoting properties [11–21]. These compounds often include flavonoids, alkaloids, sulfur compounds, and dietary fibers, all of which possess documented

antioxidant, anti-inflammatory, and lipid-lowering properties [22]One promising approach involves the use of medicinal plants.

Medicinal plants contain bioactive compounds with various health attributes. Specific examples of these plants and their key bioactive constituents are summarized in Table 1.

Sumac (*Rhus coriaria*) is rich in phenolic compounds (flavonoids and anthocyanins) [11]. Lemon (*Citrus limon*) contains vitamin C, flavonoids, and limonoids [12]. Black pepper (*Piper nigrum*) is known for its piperine alkaloids [13]. Chicory (*Cichorium intybus L.*) is rich in inulin and sesquiterpenes [14]. Milk thistle (*Silybum marianum*) contains a considerable content of silymarin, a flavonoid [15]. Garlic and shallot (*Allium sativum*, *Allium cepa*) possess allicin, an organosulfur compound [16]. Artichoke (*Cynara scolimus*) is known for its inulin and oligofructose content, which are beneficial fibers [17]. Green tea (*Camellia sinensis*) contains catechins, a group of flavonoids [18]. Turmeric (*Curcuma longa*) bioactive compound is curcumin [19] and bioactive compounds in fumitory (*Fumaria officinalis*) are protopine and fumaric acid [20]. Ginger (*Zingiber officinale*) consists of gingerol, gingerdiol, and gingerdione [21]. In addition to reducing economic losses, herbal interventions align with animal welfare priorities by addressing the pain and suffering associated with FLHS [4, 7]. As global egg demand rises, enhancing the health and productivity of laying hens is vital for sustainable poultry production. Therefore, developing effective dietary strategies remains a key research priority.

While individual medicinal herbs have been examined for their potential benefits in laying hens, the efficacy of combinations of these herbal additives in preventing or mitigating FLHS has not been thoroughly investigated.Bioactive compounds in these medicinal plants have shown antioxidant, anti-inflammatory, and lipid-lowering properties [22]. Although the use of individual medicinal herbs has been examined in laying hens, combinations of them have not been studied

~~particularly with regard to FLHS.~~ Thus, the objectives of the present study were to develop an appropriate formulation of herbal additives in laying hen diets to evaluate productive responses, blood parameters, and the development of FLHS.

Table 1. Medicinal plants and their key bioactive compounds

**Plant**	**Scientific name**	**Main bioactive compounds**	**References**
Sumac	*Rhus coriaria*	Flavonoids, anthocyanins	[11]
Lemon	*Citrus limon*	Vitamin C, flavonoids, limonoids	[12]
Black pepper	*Piper nigrum*	Piperine alkaloids	[13]
Chicory	*Cichorium intybus L.*	Inulin, sesquiterpenes	[14]
Milk thistle	*Silybum marianum*	Silymarin (flavonoid complex)	[15]
Garlic / Shallot	*Allium sativum, A. cepa*	Allicin (organosulfur compound)	[16]
Artichoke	*Cynara scolymus*	Inulin, oligofructose	[17]
Green tea	*Camellia sinensis*	Catechins (flavonoids)	[18]
Turmeric	*Curcuma longa*	Curcumin	[19]
Fumitory	*Fumaria officinalis*	Protopine, fumaric acid	[20]
Ginger	*Zingiber officinale*	Gingerol, gingerdiol, gingerdione	[21]

Materials and methods

Experimental design and dietary treatments

The Animal Ethics Committee of Razi University, Kermanshah, Iran approved guidelines were followed for all experimental protocols (IR.RAZI.REC.1400.051). These guidelines adhered to the EU standards for the protection of animals and/or feed legislation.

A number of 1000 fertile eggs (60.5 ± 3.5 g, Lohmann LSL-Lite strain) were ~~bought~~obtained from a local breeding company (Toyoor-Barekat, Tehran, Iran).~~The total number of one thousand fertile eggs were bought from a local hatchery company (Toyor-Barekat),~~ Fertile eggs were set in the laboratory incubator (MG-1000 model, Rcom Co., Ltd., Korea) of the Animal Science Department, Razi University, Kermanshah, Iran. Hatched chicks were then raised according to the strain guideline until the onset of the experimental trail at the age of 65 weeks [23]. A total of 384 laying hens were randomly selected from the flock. In doing so, all hens were weighed to obtain an average body weight in the flock (1,605 ± 85 g). The hens were housed in wire cages measuring 45 × 45 × 45 cm, with three birds per cage, corresponding to a stocking density of 675 cm² per bird. Two adjacent cages (containing six birds in total) were considered one replicate, resulting in eight replicates per treatment. Sample size was determined based on expected effect sizes from previous studies to achieve 80% power at α = 0.05. To minimize potential bias, all data collection and subsequent laboratory analyses were performed by personnel blinded to the dietary treatments. ~~and set in the laboratory incubator in Animal Science Department. The hatched chicks were then reared until the starting of the experimental trail based on the company catalogue. Finally, at the age of 65 weeks, the total number of 384 Lohmann LSL-Lite laying hens were randomly selected from this larger herd and distributed between 64 wire cages with dimensions of 45 × 45 × 45 cm, with six birds per cage.~~ A 16-hour light/8-hour dark lighting schedule was implemented using white LED lighting, providing an intensity of approximately 10–15 lux at bird level. The temperature-humidity index (THI) over the 12-week period is presented in Fig 1. Hens had free access to feed and water throughout the experiment.

Fig 1. Variation in temperature-humidity index (THI), temperature (°C), and relative humidity (%) over a 12-week period. The THI was calculated using the equation proposed by Marai et al. [24]:

THI = db°F – [(0.55 – 0.55 × RH) × (db°F – 58)], where RH represents the relative humidity (%) and db°F is the dry-bulb temperature in degrees Fahrenheit.

Two basal diets were formulated for the experiment: a standard diet (SD) formulated according to the strain's recommended guidelines (2500 kcal/kg AME and 15% crude protein) [23]~~-~~, and a challenge diet (CD) having high energy and low protein contents (3100 kcal/kg AME and 12% crude protein) designed to induce FLHS~~designed to induce FLHS included high energy and low protein contents (3100 kcal/kg AME and 12% crude protein)~~. Within each basal diet, there were four dietary interventions including three herbal mixes along with a control with no herbal additives. Mix 1 included turmeric, fumitory, green tea, and milk thistle. Mix 2 contained lemon, black pepper, sumac, and chicory. Mix 3 comprised garlic, artichoke, ginger, and shallot. The experimental diets and their chemical composition are presented in Table ~~1~~2. A two-week adaptation period was implemented to acclimate hens to the diets prior to the 12-week experimental trial~~The experimental diets were fed to laying hens for two weeks in order to adapt to new diets before running into the experimental trial~~. The adaptation period was considered to minimize the error effect caused by the changes in the diets. The experimental trial lasted for 12 weeks (weeks 65 through 77).

Table 2. Ingredients and composition of the treatment diets.

**Ingredients (%)**	**SD**	**CD**
Corn	57	66
Soybean meal	24	17
Wheat bran	6.4	-
Soybean oil	1	6

Methionine	0.15	0.15
Salt	0.2	0.2
Sodium bicarbonate	0.1	0.1
Vitamin premix[a]	0.25	0.25
Mineral premix[b]	0.25	0.25
Oyster shell	9.2	9.2
Dicalcium phosphate	1.5	1.5
Multi enzyme containing phytase	0.05	0.05
**Nutrient composition**		
ME (Kcal/kg)	2500	3100
Crude protein (%)	15	12
Crude fiber (%)	2.1	2.1
Calcium (%)	3.7	3.7
Sodium (%)	0.13	0.13
Available phosphorus (%)	0.36	0.36

Treatment diets: SD (standard diet = normal protein and normal ME); CD (challenge diet = low protein and high ME)

[a] Vitamin mixture per 0.3 kg/100 kg of diet: Vitamin A, 7,700,000 IU; Vitamin D3, 3,300,000 IU; Vitamin E, 6600 mg; VitaminK3, 550 mg; thiamine,2200 mg; riboflavin, 4400 mg; Vitamin B6, 4400 mg; capantothenate, 550 mg; nicotinic acid, 200 mg; folic acid, 110 mg; choline chloride, 275,000 mg; biotin,55

mg; Vitamin B12, 8.8 mg. [b]Mineral mixture per 0.3 kg/100 kg of diet: Mn, 66,000 mg; Zn, 66,000 mg; Fe, 33,000 mg; Cu, 8,800 mg; Se, 300 mg.

Measurements

Gas chromatography-mass spectrometry (GC-MS)

Volatile compounds of herbal ~~The volatile compounds within the plant~~ samples were identified using a headspace (HS) sampling technique followed by GC-MS analysis. ~~-~~A qualitative GC-MS analysis was performed with an HP 6890 gas chromatograph interfaced with an HP 5975C Mass Selective Detector (Agilent Technologies, Foster City, CA, USA). The analysis employed electron ionization (EI) mode at 70 eV and followed the procedure described by Naghneh et al. [25]. The composition of herbs has shown in supplementary~~Additional~~ file 1.

Productive performance

Egg ~~production~~ ~~number~~count and ~~egg~~ weight were recorded on a daily basis, and these~~the~~ data were used to calculate hen-day egg production (EP), and egg weight (EW). Moreover, feed conversion ratio (FCR) was calculated based on feed intake (FI) and egg performance data [26].

Blood parameters

At weeks 65 (the start), 70 (the middle) and 77 (the end of trial), two laying hens from each replicate (16 hens per treatment) ~~was~~ were randomly selected for blood collection from the wing vein. A sample of 3 ml blood was collected in a test tube without any anticoagulant.~~and 3 ml of blood was collected in a test tube without any anticoagulant.~~ ~~The~~ B~~b~~lood samples were immediately centrifuged at 3000 rpm for 10 minutes and the resulting sera were stored at -20°C until laboratory tests. The following parameters were determined according to the manufacturer's instructions using commercial diagnostic kits (Pars Azmun, Iran)~~to the manufacturer's guidelines~~:

aspartate aminotransferase (AST), total protein (TP), total triglyceride (TG), total cholesterol (TC), low-density cholesterol (LDL), high-density cholesterol (HDL), uric acid (UA), and hemoglobin (Hb) (Parsazmun, Tehran, Iran) [27]. In addition, a smear of blood was prepared from each blood sample ~~and sent to the laboratory~~ to count heterophiles and lymphocytes.

Hepatic histopathology and scoring system

During the experimental period, all mortalities were ~~mortality was~~ weighed and necropsied, and the incidence of FLHS was assessed in all treatment groups. At the end of trial (Week 77), ~~a single bird~~one hen from each cage (8 hens per treatment) were randomly selected, ~~and the selected laying hens were~~ anaesthetized via inhalation of 60% CO_2, and then sacrificed by cervical dislocation. CO_2 was delivered from compressed gas canister and delivered using a gradual fill method with a displacement rate of 60% via the chamber volume per minute using of a flowmeter. The~~ir~~ livers were harvested and dissected for further analysis. To assess histological examinations, liver tissues were fixed in 10% buffered formalin and processed by a microtome. These sections were then stained with hematoxylin and eosin (H&E) before examination under a light microscope ~~and observed under a light microscope~~ [~~24~~28]. A four-point scoring system was employed to evaluate the severity of ~~fatty liver~~ FLHS in the chicken livers, based on the presence and extent of acentric nuclei within hepatocytes. Acentric nuclei are displaced from their central position due to the accumulation of fat droplets within the cells. Scores were ranged from 1 (indicating normal liver ~~pathology~~ with centrally located nuclei) to 4 (representing severe FLHS with extensive acentric nuclei), with 2 and 3 signifying mild and moderate degrees of acentricity, respectively (Fig 2).

Fig 2. Representative liver histopathology under H&E staining at increasing grades of fatty change. (A) Normal liver shows uniform architecture with centrally located nuclei (score 1). (B) Mild fatty change (score 2) exhibits minimal fatty vacuoles (clear spaces) within hepatocytes. (C)

Formatted: Subscript

Moderate fatty change (score 3) demonstrates increased cytoplasmic vacuolization. (D) Severe fatty change (score 4) displays extensive fatty infiltration obscuring most cytoplasm. Arrowhead highlights an acentric nucleus, a characteristic finding in steatosis.

Statistical analysis

The study was conducted in a completely randomized design using a 2×4 factorial layout. Data were subjected to the GLM procedures of SAS 9.4 software [29~~25~~] according to the following model:

$$Y_{ijk} = \mu + A_i + B_j + ABij + e_{ijk}$$

where Y_{ijk} = observed value for a particular trait, μ= overall mean, A_i = effect of the basal diet (SD and CD), B_j = effect of the herbal mixture, AB_{ij} = the respective interaction of ith and jth levels of dietary SDCD and herbal additives, and e_{ijk} = random error associated with the ijkth recording. The treatment means were separated by Duncan's multiple-range test at P $\leq$ ~~<~~0.05 statistical level. The effects of the main factors were not considered, whenever the interaction was significant. The non-parametric method of Kruskal-Wallis test was used to analyze liver histopathology scores [29].

Results

Laying performance

Tables 3, 4, 5, and 6 present the effects of dietary interventions on FI, EP, EW, and FCR of laying hens, respectively. Although no significant differences in FI were observed among treatments during the first four weeks of the experiment, both the main effects of the basal diets (SD vs. CD) and the herbal additives became significant in weeks 5 and 6 (Table 3).~~Although the difference~~

~~between the treatments was not significant with respect to FI during the first 4 weeks of the experiment, the main effects of SD and CD, as well as the main effects of herbal additives in weeks 5 and 6 were significant (Table 2).~~ Hens fed the CD showed reduced FI compared to those on the SD (P = 0.0001). Among the herbal treatments, Mix 2 led to the highest FI during weeks 5 and 6 (P = 0.0053 and P = 0.0003, respectively). From weeks 7 to 12, a significant interaction between dietary treatments and FI was observed (P = 0.0153, P = 0.0015, P = 0.0036, P = 0.0061, P = 0.0034, and P = 0.0031, respectively). During this period, hens receiving the SD, with or without herbal additives, generally showed higher FI. Among the CD groups, CD + Mix 2 resulted in the highest FI and outperformed the other CD-based treatments (Table 3).

~~Feeding laying hens with the CD reduced FI as opposed to the SD (*P* = 0.0001). Additionally, Mix 2 had the highest FI compared to other groups in weeks 5 and 6 were significant (*P* = 0.0053 and *P* = 0.0003, respectively). From week 7 through 12, a notable interaction effect of the experimental treatments was observed for FI of laying hens (). Laying hens fed with SD with or without herbal mixes had higher FI, followed by the CD + Mix 2, which were superior to other treatments (Table 2).~~

EP rate was not significantly affected by the experimental treatments during the first ~~4~~ four weeks of the trial (P > 0.05) (Table 4). From w~~W~~eek 5 onward, the interaction effects of SD and CD plus herbal mixes on EP were significant (P ~~<~~ = 0.~~05~~0001). ~~Throughout~~Throughout the trial, the CD and ~~the~~ CD with herbal mixes showed reduced EP compared to the SD and the SD with herbal mixes. The exception was the CD + Mix 2, which ~~had~~ showed no significant difference ~~with~~ from the SD group (P > 0.05).

No significant differences ~~was~~ were observed ~~with respect to~~in the EW of laying hens during the first four weeks -(P > 0.05), except for ~~the~~ a significant interaction effect~~s~~ ~~of the~~in the first week

and ~~the~~ a main effect of herbal additives in week 3 ~~(*P* < 0.05)~~ (Table 5). In the first week, the group fed with SD had ~~more~~ greater EW compared to the SD + Mix 1 (P = 0.0342).~~,~~ In ~~and in~~ the third week, Mix 2 ~~had~~ resulted in a higher EW relative to the control group (P ~~<~~ = 0.~~05~~0445). In addition, the main effects of SD and CD were significant from weeks 5 through 12, ~~so that the~~with laying hens fed ~~with~~ the CD ~~had~~ having lower EW than those fed the SD (P ~~<~~ = 0.~~05~~0001). From~~In~~ weeks 5 to 9, the main effects of herbal additives on EW were significant.~~,~~ ~~and the~~ D~~d~~iets containing~~ed~~ Mix 2 and Mix 3 consistently ~~had~~ resulted in higher EW than the control diet (P ~~<~~ = 0.~~05~~0002, P = 0.0002, P = 0.0005, and P = 0.0044, respectively).

FCR was not affected by the experimental treatments between weeks 1 to 4 and weeks 9 to 12 (P > 0.05) (Table 6). The interaction effects of SD and CD with herbal additives were significant during weeks 5 to 8 ~~(*P* < 0.05)~~. In week 5, the lowest FCR was ~~related~~ observed ~~to~~ in the SD, SD + Mix 3, and CD + Mix 2, which were significantly different from CD, CD + Mix 1, and CD + Mix 3 (P = 0.0035~~*P* < 0.05~~). In week 6, the lowest FCR belonged to the CD+ Mix 2 and SD diets, ~~and~~ while the highest FCR was related to CD, CD + Mix 4, and CD + Mix 3 (P = 0.0048). In week 7, the CD + Mix 2, SD, SD + Mix 3, and CD groups had the lowest FCR and the CD + Mix 1 and CD + Mix 3 groups had the highest (P = 0.0239). Moreover, CD + Mix 2 and CD + Mix 3 had the lowest and highest FCR, respectively, when ~~obtained~~ measured at week 8 (P ~~<~~ = 0.~~05~~0467).

Table 3. Effect of different combinations of herbal additives with standard and challenge diet on the feed intake of laying hens during weeks 1 to 12 of the trial (65 to 77 weeks of age)

**Treatments**	**Feed intake (g/hen/day)**											
	Week1	Week2	Week3	Week4	Week5	Week6	Week7	Week8	Week9	Week10	Week11	Week12
**Main effects**												
SD	114.56	116.06	111.37	120.97	117.40^{a}	117.84^{a}	117.96^{a}	118.19^{a}	118.53^{a}	118.44^{a}	118.78^{a}	118.84^{a}
CD	114.40	116.40	110.69	118.62	105.34^{b}	103.62^{b}	102.84^{b}	103.31^{b}	96.87^{b}	96.87^{b}	96.87^{b}	96.87^{b}
C	113.44	114.31^{b}	110.75	117.00	110.44^{b}	108.81^{b}	107.19^{c}	107.62^{c}	103.50^{c}	103.31^{c}	104.00^{c}	104.12^{b}
Mix 1	113.31	114.81^{ab}	110.31	119.62	109.50^{b}	109.50^{b}	109.50^{bc}	109.50^{bc}	104.37^{c}	104.37^{bc}	104.37^{bc}	104.37^{b}
Mix 2	115.50	117.75^{a}	112.12	122.81	114.37^{a}	114.06^{a}	114.06^{a}	115.00^{a}	114.12^{a}	114.12^{a}	114.12^{a}	114.12^{a}
Mix 3	115.69	118.06^{a}	110.94	119.75	111.19^{b}	110.56^{b}	110.87^{b}	110.87^{b}	108.81^{b}	108.81^{b}	108.81^{b}	108.81^{b}
**SEM**	12.01	20.13	22.21	55.17	15.20	11.81	17.36	18.07	37.24	39.97	40.07	40.23
**Interactions**												
SD	113.13	112.13	110.88	116.25	115.13	116.88	117.38^{a}	118.25^{a}	117.13^{a}	116.75^{a}	118.13^{a}	118.38^{a}
SD + Mix 1	113.75	116.63	110.38	123.25	116.50	116.50	116.50^{a}	116.50^{a}	116.88^{a}	116.88^{a}	116.88^{a}	116.88^{a}
SD + Mix 2	115.00	116.88	112.38	121.75	119.38	119.38	119.38^{a}	119.38^{a}	120.00^{a}	120.00^{a}	120.00^{a}	120.00^{a}
SD + Mix 3	116.38	118.63	111.88	122.63	118.63	118.63	118.63^{a}	118.63^{a}	120.13^{a}	120.13^{a}	120.13^{a}	120.13^{a}
CD	113.75	116.50	110.63	117.75	105.75	100.75	97.00^{d}	97.00^{d}	89.88^{d}	89.88^{d}	89.88^{d}	89.88^{d}
CD + Mix 1	112.88	113.00	110.25	116.00	102.50	102.50	102.50^{c}	102.50^{c}	91.88^{cd}	91.88^{cd}	91.88^{cd}	91.88^{cd}
CD + Mix 2	116.00	118.63	111.88	123.88	109.38	108.75	108.75^{b}	110.63^{b}	108.25^{b}	108.25^{b}	108.25^{b}	108.25^{b}
CD + Mix 3	115.00	117.50	110.00	116.88	103.75	102.50	103.13^{c}	103.13^{c}	97.50^{c}	97.50^{c}	97.50^{c}	97.50^{c}
**SEM**	3.46	4.49	4.71	7.43	3.90	3.44	4.16	4.25	6.10	6.32	6.33	6.34
**Significant levels**												
SDCD	0.8575	0.7604	0.5619	0.2121	0.0001	0.0001	0.0001	0.0001	0.0001	0.0001	0.0001	0.0001
HA	0.0985	0.0377	0.7306	0.1908	0.0053	0.0003	0.0002	0.0001	0.0001	0.0001	0.0001	0.0001
SDCD*HA	0.7264	0.0780	0.9496	0.1771	0.1202	0.0889	0.0153	0.0015	0.0036	0.0061	0.0034	0.0031

SD=standard diet; CD=challenge diet; C=control (without additive); Mix 1= turmeric, fumitory, green tea and milk thistle powder; Mix 2= lemon, black pepper, sumac and chicory powder; Mix 3= garlic, artichoke, ginger and shallot powder; HA= herbal additive; SDCD*HA= interaction effect of dietary treatments.

a–d Means with no common superscript within each column are significantly ($P \leq 0.05$) different.

SEM= standard error of the mean.

Table 4. Effect of different combinations of herbal additives with standard and challenge diet on the egg production ratio (%) of laying hens during weeks 1 to 12 of the trial (65 to 77 weeks of age)

Treatments	Hen-day egg production (%)											
	Week1	Week2	Week3	Week4	Week5	Week6	Week7	Week8	Week9	Week10	Week11	Week12
**Main effects**												
SD	88.37	86.68	85.03	85.81	84.84^{a}	84.37^{a}	83.78^{a}	83.41^{a}	82.09^{a}	81.25^{a}	80.69^{a}	80.40^{a}
CD	88.58	86.86	85.05	85.68	75.59^{b}	75.50^{b}	75.50^{b}	75.53^{b}	71.66^{b}	71.28^{b}	70.34^{b}	70.34^{b}
C	88.44	87.20	85.53	86.42	80.87^{b}	79.87^{b}	79.75^{b}	79.25^{b}	75.12^{bc}	73.19^{c}	71.81^{b}	71.25^{b}
Mix 1	88.67	86.69	85.03	85.61	78.56^{c}	78.56^{b}	78.31^{bc}	78.31^{b}	74.00^{c}	73.50^{c}	72.69^{b}	72.69^{b}
Mix 2	88.00	86.64	84.84	85.64	83.31^{a}	83.25^{a}	82.87^{a}	82.81^{a}	80.69^{a}	80.69^{a}	80.31^{a}	80.31^{a}
Mix 3	88.81	86.55	84.75	85.31	78.12^{c}	78.06^{c}	77.62^{c}	77.50^{b}	77.69^{b}	77.69^{b}	77.25^{a}	77.25^{a}
**SEM**	1.83	1.79	1.63	1.43	5.76	5.87	5.86	6.23	15.89	16.76	20.51	20.89
**Interactions**												
SD	88.62	87.40	85.69	86.87	86.75^{a}	85.12^{a}	84.87^{a}	83.75^{a}	84.75^{a}	81.37^{a}	80.12^{a}	79.00^{a}
SD + Mix 1	88.37	86.12	84.50	85.19	84.00^{b}	84.00^{ab}	83.50^{a}	83.50^{a}	81.12^{a}	81.12^{a}	80.50^{a}	80.50^{a}
SD + Mix 2	87.75	86.31	85.06	85.94	84.25^{ab}	84.12^{ab}	83.37^{a}	83.25^{a}	81.00^{a}	81.00^{a}	80.75^{a}	80.75^{a}
SD + Mix 3	88.75	86.87	84.87	85.26	84.37^{ab}	84.25^{ab}	83.37^{a}	83.12^{a}	81.50^{a}	81.50^{a}	81.37^{a}	81.37^{a}
CD	88.25	87.00	85.37	85.97	75.00^{c}	74.62^{c}	74.62^{b}	74.75^{b}	65.50^{c}	65.00^{c}	63.50^{c}	63.50^{c}
CD + Mix 1	88.97	87.25	85.56	86.04	73.12^{cd}	73.12^{cd}	73.12^{bc}	73.12^{bc}	66.87^{c}	65.87^{c}	64.87^{c}	64.87^{c}
CD + Mix 2	88.25	86.97	84.62	85.34	82.37^{b}	82.37^{b}	82.37^{a}	82.37^{a}	80.37^{a}	80.37^{a}	79.87^{a}	79.87^{a}
CD + Mix 3	88.86	86.22	84.62	85.36	71.87^{d}	71.87^{d}	71.87^{c}	71.87^{c}	73.87^{b}	73.87^{b}	73.12^{b}	73.12^{b}
**SEM**	1.82	1.78	1.63	1.43	5.75	2.42	2.42	2.50	3.99	4.09	4.53	4.57
**Significant levels**												
SDCD	0.5383	0.5835	0.9612	0.6477	0.0001	0.0001	0.0001	0.0001	0.0001	0.0001	0.0001	0.0001
HA	0.3576	0.5193	0.3221	0.0667	0.0001	0.0001	0.0001	0.0001	0.0001	0.0001	0.0001	0.0001
SDCD*HA	0.7334	0.1988	0.3151	0.1774	0.0001	0.0001	0.0001	0.0001	0.0001	0.0001	0.0001	0.0001

SD=standard diet; CD=challenge diet; C=control (without additive); Mix 1= turmeric, fumitory, green tea and milk thistle powder; Mix 2= lemon, black pepper, sumac and chicory powder; Mix 3= garlic, artichoke, ginger and shallot powder; HA= herbal additive; SDCD*HA= interaction effect of dietary treatments.
a–d Means with no common superscript within each column are significantly ($P \leq 0.05$) different.
SEM= standard error of the mean.

Table 5. Effect of different combinations of herbal additives with standard and challenge diet on the egg weight (g) of laying hens during weeks 1 to 12 of the trial (65 to 77 weeks of age)

Treatments	Egg weight (g)											
	Week1	Week2	Week3	Week4	Week5	Week6	Week7	Week8	Week9	Week10	Week11	Week12
**Main effects**												
SD	62.56	63.62	63.17	64.69	64.37^{a}	64.37^{a}	64.43^{a}	64.39^{a}	64.51^{a}	64.56^{a}	64.65^{a}	64.75^{a}
CD	62.47	63.58	63.36	65.25	61.75^{b}	61.09^{b}	61.00^{b}	61.09^{b}	61.19^{b}	60.87^{b}	60.81^{b}	61.00^{b}
C	62.56	63.31	62.69^{b}	64.94	62.59^{b}	62.03^{b}	61.91^{b}	61.87^{b}	62.16^{b}	62.16^{b}	62.31	62.50
Mix 1	62.31	63.42	63.37^{ab}	64.20	62.53^{b}	61.84^{b}	61.91^{b}	62.09^{b}	62.14^{b}	62.06^{b}	62.16	62.21
Mix 2	62.62	63.81	63.81^{a}	65.49	63.44^{a}	63.43^{a}	63.44^{a}	63.41^{a}	63.37^{a}	63.06^{ab}	62.94	63.12
Mix 3	62.56	63.87	63.19^{ab}	65.25	63.69^{a}	63.62^{a}	63.62^{a}	63.59^{a}	63.72^{a}	63.59^{a}	63.53	63.69
**SEM**	1.73	1.06	1.21	2.05	1.31	1.81	1.83	1.83	2.20	3.59	4.28	4.21
**Interactions**												
SD	63.25^{a}	63.50	62.50	64.50	63.93	63.93	64.18	64.00	64.06	64.31	64.50	64.87
SD + Mix 1	61.62^{b}	63.12	63.31	64.27	63.69	63.69	63.69	63.68	63.66	63.62	63.68	63.66
SD + Mix 2	62.87^{ab}	63.87	64.00	65.11	64.50	64.50	64.50	64.43	64.87	64.87	64.87	64.87
SD + Mix 3	62.50^{ab}	64.00	62.87	64.87	65.37	65.37	65.37	65.43	65.44	65.44	65.56	65.62
CD	61.87^{ab}	63.12	62.87	65.37	61.25	60.12	59.62	59.75	60.25	60.00	60.12	60.12
CD + Mix 1	63.00^{ab}	63.71	63.44	64.12	61.37	60.00	60.12	60.50	60.62	60.50	60.62	60.75
CD + Mix 2	62.37^{ab}	63.75	63.62	65.87	62.37	62.37	62.37	62.37	61.87	61.25	61.00	61.37
CD + Mix 3	62.62^{ab}	63.75	63.50	65.62	62.00	61.87	61.87	61.75	62.00	61.75	61.50	61.75
**SEM**	1.31	1.03	1.10	1.43	1.14	1.34	1.33	1.35	1.48	1.89	2.07	2.05
**Significant levels**												
SDCD	0.7766	0.8754	0.4983	0.1240	0.0001	0.0001	0.0001	0.0001	0.0001	0.0001	0.0001	0.0001
HA	0.9111	0.3239	0.0445	0.0722	0.0094	0.0002	0.0002	0.0005	0.0044	0.0752	0.2298	0.1865
SDCD*HA	0.0342	0.5568	0.6163	0.7240	0.4319	0.2669	0.0925	0.1424	0.8498	0.8509	0.8302	0.6418

SD=standard diet; CD=challenge diet; C=control (without additive); Mix 1= turmeric, fumitory, green tea and milk thistle powder; Mix 2= lemon, black pepper, sumac and chicory powder; Mix 3= garlic, artichoke, ginger and shallot powder; HA= herbal additive; SDCD*HA= interaction effect of dietary treatments.

a–d Means with no common superscript within each column are significantly ($P \leq 0.05$) different.

SEM= standard error of the mean.

Table 6. Effect of different combinations of herbal additives with standard and challenge diet on the feed conversion ratio of laying hens during weeks 1 to 12 of the trial (65 to 77 weeks of age)

Treatments	Feed conversion ratio (g/g)											
	Week1	Week2	Week3	Week4	Week5	Week6	Week7	Week8	Week9	Week10	Week11	Week12
**Main effects**												
SD	2.07	2.10	2.07	2.17	2.15^{b}	2.17^{b}	2.19	2.20	2.23	2.26	2.27	2.28
CD	2.06	2.11	2.05	2.12	2.26^{a}	2.25^{a}	2.24	2.24	2.22	2.24	2.28	2.28
C	2.051	2.071	2.068	2.081	2.191	2.199^{ab}	2.168^{b}	2.187	2.226	2.277	2.331	2.350
Mix 1	2.054	2.091	2.050	2.175	2.233	2.259^{a}	2.275^{a}	2.262	2.268	2.290	2.312	2.306
Mix 2	2.096	2.130	2.062	2.193	2.164	2.160^{b}	2.168^{b}	2.187	2.229	2.241	2.250	2.250
Mix 3	2.084	2.136	2.075	2.150	2.242	2.232^{ab}	2.262^{ab}	2.262	2.203	2.207	2.225	2.218
**SEM**	0.008	0.011	0.011	0.025	0.013	0.010	0.016	0.015	0.034	0.037	0.044	0.046
**Interactions**												
SD	2.018	2.021	2.087	2.075	2.076^{d}	2.147^{cd}	2.150^{b}	2.200^{abc}	2.158	2.236	2.275	2.312
SD + Mix 1	2.090	2.145	2.050	2.250	2.178^{cd}	2.178^{cd}	2.212^{ab}	2.212^{abc}	2.263	2.264	2.275	2.275
SD + Mix 2	2.085	2.120	2.050	2.175	2.197^{bcd}	2.200^{bcd}	2.225^{ab}	2.225^{abc}	2.283	2.283	2.287	2.287
SD + Mix 3	2.100	2.134	2.100	2.212	2.151^{d}	2.154^{cd}	2.187^{b}	2.187^{bc}	2.253	2.253	2.250	2.250
CD	2.084	2.121	2.050	2.087	2.306^{ab}	2.251^{abc}	2.187^{b}	2.175^{bc}	2.294	2.317	2.387	2.387
CD + Mix 1	2.017	2.038	2.050	2.100	2.287^{abc}	2.341^{a}	2.337^{a}	2.312^{ba}	2.273	2.316	2.350	2.337
CD + Mix 2	2.108	2.140	2.075	2.212	2.132^{d}	2.119^{d}	2.112^{b}	2.150^{c}	2.176	2.199	2.212	2.212
CD + Mix 3	2.067	2.138	2.050	2.087	2.334^{a}	2.310^{ab}	2.337^{a}	2.337^{a}	2.153	2.161	2.200	2.187
**SEM**	0.090	0.106	0.107	0.158	0.115	0.104	0.128	0.125	0.186	0.193	0.210	0.215
**Significant levels**												
SDCD	0.8502	0.8783	0.5623	0.1610	0.0002	0.0019	0.1258	0.2352	0.7386	0.8243	0.7674	1.000
HA	0.4149	0.2614	0.9237	0.2131	0.1978	0.0516	0.0286	0.1368	0.7966	0.6151	0.4384	0.3268
SDCD*HA	0.1536	0.0618	0.7448	0.2418	0.0035	0.0048	0.0239	0.0467	0.2226	0.4632	0.5175	0.6516

SD=standard diet; CD=challenge diet; C=control (without additive); Mix 1= turmeric, fumitory, green tea and milk thistle powder; Mix 2= lemon, black pepper, sumac and chicory powder; Mix 3= garlic, artichoke, ginger and shallot powder; HA= herbal additive; SDCD*HA= interaction effect of dietary treatments.
a–d Means with no common superscript within each column are significantly ($P \leq 0.05$) different.
SEM= standard error of the mean.

Hematological criteria

Tables 7, 8, and 9 show hematological criteria of laying hens fed with herbal additives in both SD and CD at the beginning, middle, and end of the experimental period. Feeding the CD resulted in elevated levels of AST compared to the ~~standard diet~~SD ~~at~~ in the middle and at the end of the trial (P = 0.0001). The interaction effects of dietary treatments on AST enzyme levels in the middle and at the end of the trial were significant (P ~~<~~ = 0.~~05~~0001). ~~so~~ Specifically, ~~that~~ the lowest AST level was ~~related to~~observed ~~the~~ in laying hens fed with SD + Mix 3 and CD + Mix 2, ~~and~~ while the highest levels ~~was~~ were ~~related~~ associated ~~to~~ with the CD and~~,~~ CD + Mix 1 (Table 7).

The interaction effects of CD and SD with herbal additives on the concentration of TP were significant at both the middle and the end of the trial ~~(*P* < 0.05)~~ (Table 7). In the middle of the trial, hens fed the CD + Mix 2 had the highest TP concentration, ~~and~~ while those on the CD and ~~the~~ CD + Mix 1 ~~had~~ showed the lowest (P = 0.0428) ~~TP concentration~~. By the end of the trial, the highest TP concentrations were observed in the ~~The~~ CD + Mix 2, SD + Mix 2, and SD + Mix 3 groups~~had the highest TP~~, ~~and~~ whereas the ~~the~~ SD, CD, and CD + Mix 1 groups had the lowest (P = 0.050). ~~at the end of trial.~~

~~As for~~For UA, ~~in the middle and the end of experiment,~~ the interaction effects of experimental treatments were significant at both the middle and the end of the experiment ~~(*P* < 0.05)~~ (Table 7). Overall, the SD without additives resulted in the highest blood UA levels. At mid-trial, this was significantly higher than those in the SD + Mix 2, SD + Mix 3, and CD + Mix 2 groups (P = 0.0006). By the end of the trial, the SD group also showed significantly higher UA concentrations compared to SD + Mix 1, SD + Mix 2, SD + Mix 3, and CD + Mix 2 (P = 0.0004). The lowest UA concentrations were consistently observed in the SD + Mix 2 and SD + Mix 3 groups.

~~In general, the SD without additives had the highest amount of blood UA, which was significantly different from SD + Mix 2, SD + Mix 3, and CD + Mix 2 in the middle of trial and SD + Mix 1, SD +~~

Mix 2, SD + Mix 3, and CD + Mix 2, at the end of trial ($P < 0.05$). The lowest amount of UA was seen in the SD + Mix 2 and SD + Mix 3.

The main effects of SD and CD on TG concentration in the second sampling and its their interaction effect in the third sampling were significant ($P < 0.05$) (Table 8). Based on the results, feeding CD to laying hens increased TG concentration when compared to the SD (P < = 0.050001). LikewiseSimilarly, in the third sampling, birds fed with CD, CD + Mix 1, and CD + Mix 3 had the highest amount of TG, and SD + Mix 1 had the lowest (P = 0.0001).

In the second and third samplings, the interaction effects of dietary treatments on TC concentration were significant ($P < 0.05$) (Table 8). The highest amount of TC in the second sampling was observed in layers fed with CD + Mix 3, and the lowest in the diet of laying hens fed with SD + Mix 3 (P = 0.0001). In the third sampling, the highest concentration of TC was resulted from feeding CD + Mix 1 and CD + Mix 3, and the lowest was observed by feeding SD + Mix 3 (P = 0.0001).

The effect of treatments on HDL concentration exhibited a statistically significant interaction effect at the second and third samplings (P = 0.0122 and P = 0.0028, respectively,). so thaThet SD + Mix 3 group had the highest HDL concentration compared to the other treatments. (Table 8). As for LDL, the main effect of the SD and CD in the second sampling was significant in the second sampling, and with CD increased the concentration of LDL compared to the SD (P = 0.0001) ($P < 0.05$) (Table 8).

The interaction effects of diets showed a significant effect on blood Hb concentration and heterophile and lymphocyte count in both the second and third samplings ($P < 0.05$) (Table 9). In the second sampling, the CD + Mix 2 group had the highest Hb concentration, and while the lowest values were observed in SD + Mix 2 and SD + Mix 3 had the lowest(P = 0.0001) amount of Hb. In the third sampling, the highest Hb levels was were seen recorded in the CD and CD + Mix 1 groups, and the lowest inwhile SD + Mix 2 and SD + Mix 3 showed the lowest concentrations (P = 0.0002). In terms ofRegarding the number of heterophiles in the second and third samplings, the highest number count was seen in the SD + Mix 1 group, and while the lowest in was found in SD + Mix 2 and SD + Mix 3

(P = 0.0001). In the second sampling, the highest lymphocyte counts were recorded in the SD, SD + Mix 1, and all CD groups, whereas the lowest was observed in SD + Mix 2 (P = 0.0108). In the third sampling, CD + Mix 1 exhibited the highest lymphocyte count, while SD + Mix 2 showed the lowest (P = 0.0030).

Table 7. Effect of different combinations of herbal additives with standard and challenge diet on the blood parameters of laying hens (Start, Middle, End)

**Treatments**	**AST(U/L)**			**TP(G/dL)**			**UA(mg/dl)**		
	Start (wk 65)	Middle (wk 70)	End (wk 77)	Start (wk 65)	Middle (wk 70)	End (wk 77)	Start (wk 65)	Middle (wk 70)	End (wk 77)
**Main effects**									
SD	213.57	224.78^{b}	232.97^{b}	6.41	6.79^{a}	6.90^{a}	6.18	6.19^{b}	6.56^{b}
CD	213.55	259.84^{a}	295.50^{a}	6.19	6.24^{b}	6.44^{b}	6.30	6.53^{a}	7.28^{a}
C	214.13	253.62^{a}	288.56^{a}	6.21	5.97^{b}	5.99^{c}	6.19	6.79^{a}	7.56^{a}
Mix 1	212.78	253.31^{a}	279.31^{a}	6.35	6.42^{b}	6.53^{b}	6.19	6.71^{a}	7.14^{b}
Mix 2	214.59	220.31^{b}	232.06^{c}	6.28	7.28^{a}	7.40^{a}	6.26	5.91^{b}	6.33^{c}
Mix 3	212.75	242.00^{a}	257.00^{b}	6.37	6.40^{b}	6.77^{b}	6.32	6.03^{b}	6.65^{c}
**SEM**	70.06	469.77	531.16	0.279	0.559	0.430	0.122	0.262	0.332
**Interactions**									
SD	214.25	234.75^{b}	253.87^{b}	6.37	6.35^{bcd}	6.12^{c}	6.12	6.89^{a}	7.69^{a}
SD + Mix 1	209.46	232.87^{b}	235.87^{bc}	6.70	7.02^{ab}	7.06^{ab}	6.17	6.80^{a}	6.91^{b}
SD + Mix 2	218.94	225.62^{bc}	239.37^{bc}	6.46	7.12^{ab}	7.30^{a}	6.31	5.62^{c}	5.72^{c}
SD + Mix 3	211.65	205.87^{c}	202.75^{d}	6.12	6.68^{abc}	7.12^{a}	6.13	5.44^{c}	5.94^{c}
CD	214.01	272.50^{a}	323.25^{a}	6.05	5.60^{d}	5.85^{c}	6.26	6.70^{ab}	7.43^{ab}
CD + Mix 1	216.10	273.75^{a}	322.75^{a}	6.00	5.81^{d}	6.00^{c}	6.21	6.62^{ab}	7.37^{ab}
CD + Mix 2	210.23	215.00^{bc}	224.75^{cd}	6.10	7.43^{a}	7.50^{a}	6.21	6.19^{b}	6.94^{b}
CD + Mix 3	213.86	278.12^{a}	311.25^{a}	6.61	6.12^{cd}	6.41^{bc}	6.52	6.62^{ab}	7.37^{ab}
**SEM**	8.37	21.67	23.05	0.528	0.747	0.656	0.350	0.513	0.576
**Significant levels**									
SDCD	0.9917	0.0001	0.0001	0.0943	0.0046	0.0065	0.1853	0.0090	0.0001
HA	0.8956	0.0001	0.0001	0.8325	0.0001	0.0001	0.6854	0.0001	0.0001
SDCD*HA	0.0799	0.0001	0.0001	0.0870	0.0428	0.0500	0.2647	0.0006	0.0004

SD=standard diet; CD=challenge diet; C=control (without additive); Mix 1= turmeric, fumitory, green tea and milk thistle powder; Mix 2= lemon, black pepper, sumac and chicory powder; Mix 3= garlic, artichoke, ginger and shallot powder; HA= herbal additive; SDCD*HA= interaction effect of dietary treatments; AST= aspartate aminotransferase; TP= total protein; UA= uric acid.

a–d Means with no common superscript within each column are significantly ($P \leq 0.05$) different.

SEM= standard error of the mean.

Table 8. Effect of different combinations of herbal additives with standard and challenge diet on the blood parameters of laying hens (Start, Middle, End)

**Treatments**	**TG (mg/dL)**			**TC (mg/dL)**			**HDL(mg/dl)**			**LDL(mg/dl)**		
	Start (wk 65)	Middle (wk 70)	End (wk 77)	Start (wk 65)	Middle (wk 70)	End (wk 77)	Start (wk 65)	Middle (wk 70)	End (wk 77)	Start (wk 65)	Middle (wk 70)	End (wk 77)
**Main effects**												
SD	1304.28	1410.63^{b}	1485.47^{b}	210.29	199.70^{b}	200.00^{b}	5.39	6.35	6.61	46.42	52.03^{b}	55.88
CD	1434.28	1580.88^{a}	2611.97^{a}	214.59	239.71^{a}	275.19^{a}	5.71	6.51	6.25	46.63	54.44^{a}	56.21
C	1382.19	1503.31	2257.8^{a}	213.64	230.12^{a}	255.42^{a}	5.07	5.70^{b}	5.30^{c}	46.15^{b}	52.88	57.10
Mix 1	1373.44	1473.75	2195.8^{a}	215.12	233.00^{a}	258.62^{a}	5.41	5.91^{b}	5.91^{bc}	46.29^{ab}	53.88	55.59
Mix 2	1388.25	1518.75	1682.9^{b}	209.71	221.47^{a}	230.75^{b}	5.87	6.59^{ab}	6.58^{b}	47.11^{a}	53.41	56.25
Mix 3	1333.00	1487.19	2058.5^{a}	211.30	194.25^{b}	205.56^{c}	5.85	7.53^{a}	7.93^{a}	46.56^{ab}	52.78	55.25
**SEM**	127.64	272.29	120.5	108.43	423.49	531.16	2.43	2.12	1.60	1.38	3.31	9.01
**Interactions**												
SD	1305.50	1462.50	1518.87^{bc}	211.00	225.12^{b}	235.87^{c}	5.01	5.15^{b}	5.11^{c}	45.90	52.50	58.37
SD + Mix 1	1327.12	1371.87	1397.75^{c}	211.50	231.62^{ab}	233.37^{c}	4.63	5.32^{b}	5.56^{bc}	46.43	52.12	55.27
SD + Mix 2	1338.37	1453.12	1564.50^{bc}	208.93	204.19^{c}	203.37^{d}	5.60	6.43^{b}	6.60^{b}	47.15	51.93	55.75
SD + Mix 3	1245.62	1355.00	1460.75^{bc}	209.75	137.87^{d}	127.37^{e}	6.32	8.50^{a}	9.18^{a}	46.22	51.56	54.12
CD	1458.87	1544.12	2996.62^{a}	216.27	235.12^{ab}	275.00^{ab}	5.14	6.25^{b}	5.49^{bc}	46.41	53.26	55.83
CD + Mix 1	1419.75	1575.62	2993.75^{a}	218.75	234.37^{ab}	283.87^{a}	6.19	6.50^{b}	6.26^{bc}	46.13	55.63	55.90
CD + Mix 2	1438.12	1584.37	1801.25^{b}	210.49	238.75^{ab}	258.12^{b}	6.15	6.75^{b}	6.56^{b}	47.07	54.87	56.75
CD + Mix 3	1420.37	1619.37	2656.25^{a}	212.86	250.62^{a}	283.75^{a}	5.37	6.56^{b}	6.68^{b}	46.90	54.00	56.37
**SEM**	113.00	165.20	346.79	10.41	20.58	16.86	1.56	1.45	1.26	1.17	1.82	3.00
**Significant levels**												
SDCD	0.0823	0.0001	0.0001	0.1044	0.0001	0.0001	0.4174	0.6572	0.2548	0.4916	0.0001	0.6576
HA	0.5119	0.8797	0.0001	0.4703	0.0001	0.0001	0.4163	0.0033	0.0001	0.1122	0.2993	0.3221
SDCD*HA	0.6787	0.4248	0.0001	0.8758	0.0001	0.0001	0.1651	0.0122	0.0028	0.5998	0.1785	0.1501

SD=standard diet; CD=challenge diet; C=control (without additive); Mix 1= turmeric, fumitory, green tea and milk thistle powder; Mix 2= lemon, black pepper, sumac and chicory powder; Mix 3= garlic, artichoke, ginger and shallot powder; HA= herbal additive; SDCD*HA= interaction effect of dietary treatments; TG=triglyceride; TC=total cholesterol; HDL=high density lipoprotein; LDL=Low density lipoprotein.

a–d Means with no common superscript within each column are significantly ($P \leq 0.05$) different.

SEM= standard error of the mean.

Table 9. Effect of different combinations of herbal additives with standard and challenge diet on the blood parameters of laying hens (Start, Middle, End)

**Treatments**	**Hb(g/dl)**			**Heterophile**			**Lymphocyte**		
	Start (wk 65)	Middle (wk 70)	End (wk 77)	Start (wk 65)	Middle (wk 70)	End (wk 77)	Start (wk 65)	Middle (wk 70)	End (wk 77)
**Main effects**									
SD	19.41	19.72^{b}	20.69^{b}	25.84	26.54	27.77	73.03	79.95^{b}	82.36^{b}
CD	18.67	24.09^{a}	28.59^{a}	25.63	26.40	27.76	72.83	83.23^{a}	90.09^{a}
C	19.00	23.27^{a}	27.42^{a}	25.41	27.03^{b}	29.37^{a}	72.70	84.09^{a}	90.62^{a}
Mix 1	18.94	23.18^{a}	27.34^{a}	26.05	28.50^{a}	29.94^{a}	72.53	83.77^{ab}	91.65^{a}
Mix 2	17.61	21.27^{b}	21.31^{b}	25.64	25.25^{c}	26.24^{b}	72.93	77.58^{c}	77.62^{c}
Mix 3	20.61	19.91^{b}	22.50^{b}	25.83	25.12^{c}	25.50^{b}	73.56	80.94^{b}	85.00^{b}
**SEM**	6.054	4.99	10.507	0.718	2.133	5.16	1.69	16.60	22.89
**Interactions**									
SD	21.00	23.56^{b}	25.60^{bc}	25.65	27.56^{b}	30.44^{ab}	73.00	84.37^{a}	88.12^{b}
SD + Mix 1	18.61	22.74^{b}	25.17^{c}	26.11	30.50^{a}	31.51^{a}	73.25	83.54^{a}	89.81^{ab}
SD + Mix 2	17.30	16.28^{c}	15.74^{d}	25.92	24.50^{c}	24.22^{d}	72.77	73.41^{c}	74.37^{d}
SD + Mix 3	20.75	16.31^{c}	16.25^{d}	25.67	23.62^{c}	24.87^{d}	73.09	78.50^{b}	77.12^{cd}
CD	17.01	22.99^{b}	29.25^{a}	25.17	26.50^{b}	28.31^{bc}	72.40	83.81^{a}	93.12^{ab}
CD + Mix 1	19.26	23.62^{b}	29.50^{a}	25.99	26.50^{b}	28.37^{bc}	71.81	84.00^{a}	93.50^{a}
CD + Mix 2	17.92	26.25^{a}	26.87^{abc}	25.36	26.00^{b}	28.25^{bc}	73.09	81.75^{ab}	80.87^{c}
CD + Mix 3	20.47	23.50^{b}	28.75^{ab}	25.99	26.62^{b}	26.12^{cd}	74.02	83.37^{a}	92.87^{ab}
**SEM**	2.46	2.21	3.24	0.847	1.46	2.27	1.30	4.07	4.78
**Significant levels**									
SDCD	0.2298	0.0001	0.0001	0.3201	0.7016	0.9956	0.5429	0.0021	0.0001
HA	0.0721	0.0001	0.0001	0.1904	0.0001	0.0001	0.1391	0.0001	0.0001
SDCD*HA	0.0923	0.0001	0.0002	0.4563	0.0001	0.0001	0.0632	0.0108	0.0030

SD=standard diet; CD=challenge diet; C=control (without additive); Mix 1= turmeric, fumitory, green tea and milk thistle powder; Mix 2= lemon, black pepper, sumac and chicory powder; Mix 3= garlic, artichoke, ginger and shallot powder; HA= herbal additive; SDCD*HA= interaction effect of dietary treatments; Hb=hemoglobin.

a–d Means with no common superscript within each column are significantly ($P \leq 0.05$) different.

SEM= standard error of the mean.

~~The highest number of lymphocytes in the second sampling was related to SD, SD + Mix 1, and all of the CDs, and the lowest was found in SD + Mix 2. Moreover, in the third sampling, CD + Mix 1 and SD + Mix 2 had the highest and lowest number of lymphocytes, respectively (*P* < 0.05).~~

Formatted: Font: 12 pt

Hepatic histopathology

Fig 3 illustrates the effects of various herbal mixes on the frequency of liver histopathology scores ~~of~~ in laying hens. Statistical analysis revealed a significant difference (P ~~<~~ = 0.~~05~~002) ~~in these scores~~ ~~between~~ among the treatment groups. Notably, hens fed ~~diets containing~~the SD + Mix 2 and CD + Mix_2 exhibited significantly lower liver scores compared to those fed CD without additive. No significant differences were observed among the other experimental treatments (P > 0.05)~~The difference between other experimental treatments was not significant (*P* > 0.05)~~.

Fig 3. The Effect of different combinations of herbal additives with standard and challenge diet on the liver histopathology score of laying hens. SD, standard diet; CD, challenge diet; Mix 1= turmeric, fumitory, green tea and milk thistle powder; Mix 2= lemon, black pepper, sumac and chicory powder; Mix 3= garlic, artichoke, ginger and shallot powder. Different letters indicate significant difference among different treatments at the 0.05 level

Discussion

This study ~~investigated~~ aimed to investigate the effects of various medicinal plant mixtures on laying hen performance, blood chemistry, and liver health. These mixtures were ~~provided~~ administered alongside either a SD or a CD. Among the tested ~~mixtures~~combinations, hens ~~fed~~ receiving a CD supplemented with Mix 2 (~~containing~~ comprising lemon, black pepper, sumac, and

chicory) ~~exhibited~~ demonstrated significant improvements in EP and FCR, while also ~~alleviating~~ mitigating ~~negative~~ adverse effects on ~~the~~ liver health.

The chemical composition of these herbal mixtures can ~~vary~~ exhibit variability depending on the plant species, growth conditions, and extraction methods employed. The GC-MS analysis of the herbal ~~plant~~ essential oils revealed that a ~~large~~ substantial portion of the identified compounds in each oil ~~belonged to a group of~~were phthalates. Phthalides, ~~are~~ a class of secondary metabolites, ~~with a wide~~possess a broad ~~range of~~spectrum of pharmacological activities, including anti-inflammatory and antimicrobial activities [30]. ~~Moreover~~Furthermore, most essential oils contained terpenes, a well-studied group ~~known~~ recognized for their anti-inflammatory and antioxidant effects [31]. Examples identified in this study included caryophyllene, beta-bisabolene, limonene, and α-copaene, ~~and several~~among others. Additionally, the presence of organosulfur compounds, another class of potentially beneficial natural products, was observed in garlic and shallot ~~garlic and shallot displayed the presence of organosulfur compounds, another class of potentially beneficial natural products~~ [16]. ~~Interestingly~~Notably, curcumin, a curcuminoid with ~~recognized~~ established health benefits, constituted the predominant~~was the dominant~~ component in turmeric essential oil [19].

In this study, the CD was formulated with high energy and low protein content to induce FLHS [32]. Biologically, the high energy intake relative to protein causes an imbalance in macronutrient metabolism, favoring lipogenesis over protein synthesis [33]. This results in excessive fat deposition, particularly in the liver, which impairs liver function and overall metabolic homeostasis [33].

Feeding the CD significantly decreased FI, EW, and EP rate from week 5 onward, with a concomitant increase in FCR ~~as~~ compared to feeding a SD. These declines in productive

performance can be logically attributed to altered satiety signaling and metabolic stress [34]. Specifically, the accumulation of lipids in the liver and adipose tissue leads to increased leptin secretion—a hormone known to suppress appetite—thereby reducing FI [32, 35]. Reduced nutrient intake then contributes to a decline in EP and EW, as laying hens prioritize survival over reproduction under physiological stress. Additionally, impaired liver function can reduce the synthesis of essential compounds for egg formation, such as yolk precursors and lipoproteins, further compromising productivity [36]. Similar to these~~is~~ ~~study~~findings, a previous study documented a decline in EW, EP rate, and FI in hens fed a CD [32]. Notably, these reductions began in weeks 2, 3, and 4 for each measure, respectively, and affected both young and old hens [32]. Moreover, another study reported that hens fed a CD consumed less feed and had a lower EP rate than hens on a control diet [7]. ~~It is assumed that energy consumption plays a key role in controlling FI [30].~~ The metabolic consequences of the CD extend beyond FI and egg parameters. High hepatic lipid accumulation is a precursor to FLHS, which not only physically damages the liver but also induces systemic inflammation [37]. Inflammatory responses and oxidative stress disrupt hormonal regulation and blood flow to reproductive organs, further diminishing productivity [4, 7, 37, 38]~~When a CD with a high ratio of energy to protein is fed to hens, more fat is accumulated in the liver and body tissues. Body lipid reserves secret leptin, a peptide hormone, which reduces FI [29, 31]. Excessive lipid accumulation in the liver predisposes laying hens to FLHS. This condition triggers inflammation and discomfort, leading to a decline in EP and a rise in FCR. FLHS in laying hens can be developed through a combination of factors, including impaired lipid metabolism, hormonal imbalances, oxidative stress, disruption of the metabolism and utilization of nutrients, impaired vitamin D metabolism, inflammation, and reduced blood flow to the reproductive organs [4, 7, 32, 33]~~. In the current study, the CD + Mix 2 largely compensated

for the damage caused by the CD~~ to a large extent~~, ~~and improved ~~improving FI, EP, and FCR of laying hens. These improvements are logically attributed to the biological activities of the mix's components. The herbs in Mix 2 contain a variety of secondary metabolites—such as flavonoids, phenolic acids, and terpenoids—with proven antioxidant, anti-inflammatory, and lipid-lowering properties. These compounds may inhibit de novo lipogenesis, enhance lipid catabolism, and protect hepatic tissue from oxidative damage, thereby restoring liver function and metabolic balance [11–14, 22, 39]~~The improvement is believed to be due to Mix 2's ability to inhibit fat production, enhance fat burning, reduce oxidative stress, and suppress liver inflammation [34]. Sumac, lemon, black pepper and chicory, all components of Mix 2, possess various bioactive compounds with antioxidant, anti-inflammatory, and lipid-lowering properties [11-14, 22]~~. Two theories have been proposed ~~in ~~for the pathogenesis of FLHS. The first theory ~~proposes ~~suggests that FLHS ~~starts ~~begins with the excessive deposition of ~~triglyceride ~~TG in the liver, ~~which~~ disrupting the homeostasis of lipid metabolism [40]. The second theory proposes that oxidative stress in FLHS induces excessive reactive oxygen species in the liver, ~~which leads~~leading to the destruction of hepatic connective tissues, called reticulin [40]. By mitigating both lipid accumulation and oxidative stress, Mix 2 likely targets both pathways of FLHS progression, leading to improved liver histopathology and overall hen health. Furthermore, the combined use of lemon, sumac, black pepper, and chicory may exert synergistic effects, enhancing the therapeutic impact beyond what individual herbs could achieve.~~The antioxidant and antihyperlipidemic properties of secondary metabolites in the Mix 2 could have helped improving the liver function and overall health. It is worth noting that the simultaneous use of these herbal additives could have a synergistic effect compared to using them alone.~~ Limited information is available about the role of medicinal plants in ~~prevention ~~preventing ~~of ~~FLHS in laying hens. A

previous study found that adding a mixture of chicory, turmeric, artichoke, and milk thistle powder to a high energy/low protein diet did not affect EP or FCR in laying hens [41]. However, artichoke and thistle powder reduced FI, while turmeric powder increased EW compared to the control group [41]. Another study observed that broiler chickens fed diets containing chicory root powder showed significant improvement in growth performance, likely due to increased food digestion and absorption caused by changes in jejunum histomorphometry [42]. Supplementation with 0.5% sumac in feed was found to slightly increase EW in another study, although FI, FCR, and EP were not significantly affected [43]. Research has shown that adding lemon essential oil to quail feed improved FCR and FI, possibly due to the active compounds and the~~,~~ antioxidant~~,~~ and antimicrobial properties of the oil [44]. A recent study demonstrated that incorporating garlic into laying hen diets could increase EW [45]. Additionally, another study reported increased EP, EW, and FI in groups treated with inulin [46]. Inconsistencies observed across these studies may be attributed to factors such as variations in dosage, limited absorption of the additives, and potential interactions between different herbal plants.

Laying hens fed ~~on~~ the CD, with or without herbal additives, ~~had~~ exhibited higher blood AST levels compared to those on the SD ~~in~~ at both the middle ~~(second sampling)~~ and the ~~the~~ end ~~(third sampling)~~ of the experiment. This elevation suggests hepatocellular injury, as AST is a key intracellular enzyme released into circulation when liver cells are damaged or inflamed [47]. The high energy and low protein content of the CD likely promote excessive hepatic lipid accumulation, which disrupts liver architecture, induces oxidative stress, and compromises hepatocyte integrity, thereby increasing AST release into the bloodstream [32, 48]. Notably, the inclusion of Mix 2 in the CD-fed groups mitigated the rise in AST, indicating a potential protective effect of its herbal constituents on liver health. The antioxidant and anti-inflammatory properties

of lemon, black pepper, sumac, and chicory—each rich in bioactive phytochemicals such as polyphenols, flavonoids, and terpenes—are likely responsible for reducing oxidative damage and stabilizing hepatocyte membranes [11, 13, 49]. This supports the hypothesis that phytogenic additives can attenuate liver injury under metabolic stress conditions [22].~~In this situation, supplementing CD with the Mix 2 restored the response.~~ These findings align with previous reports indicating that high-energy, low-protein diets are associated with increased AST levels in laying hens, reflecting diet-induced hepatic stress and early stages of fatty liver development [32, 48]~~AST is a liver enzyme released into the bloodstream when liver cells are damaged. Hence, a high level of AST in the blood is an indicator of liver damage or inflammation [42]. Studies have shown that feeding hens a high-energy, low-protein diet can increase AST levels [29, 43]~~. While direct studies on the effects of medicinal plants on FLHS in laying hens are scarce, related research in avian species suggests beneficial outcomes. For instance, lemon essential oil supplementation in quail diets has been shown to reduce AST levels, potentially due to cytoprotective and antioxidative actions [44]. However, results across studies remain inconsistent. Some research found that sumac supplementation had no significant effect on AST concentrations [50, 51], and others reported that combinations of chicory, turmeric, artichoke, and milk thistle did not reduce AST levels when included in a CD for laying hens [41]. In contrast, other studies have demonstrated that herbal blends containing milk thistle significantly lowered AST in hens with fatty liver, highlighting the hepatoprotective role of specific plant constituents [52]. ~~Although there is limited information on the effect of medicinal plants on FLHS in laying hens, the antioxidant and anti-inflammatory effects of these herbal additives can assist in improving liver health and decreasing AST concentration [11, 13, 44]. For example, one study found that feeding quails with lemon essential oil reduced AST levels, possibly due to the oil's protective effects on cells [39]. However, other~~

~~studies have shown that sumac supplementation does not significantly affect AST levels [45, 46]. Similarly, a recent study found that including chicory, turmeric, artichoke, and milk thistle powder in a high-energy, low-protein diet did not significantly impact AST concentration in laying hens [36]. In contrast, another study showed that adding a mixture containing milk thistle to the diet of hens with fatty liver reduced blood AST concentration [47].~~ These ~~differing~~ contrasting results may be ~~due to~~attributed to various factors, ~~such as~~including the dosage of ~~the~~ herbal additives, interactions ~~between~~ among different herbs, or the quality of the herbs used.

All herbal mixtures ~~used~~ incorporated into ~~in~~ the SD increased blood TP concentration compared to the control (SD without additives). This elevation in TP may reflect improved dietary protein utilization and metabolic efficiency, potentially mediated by enhanced gut integrity, nutrient absorption, and systemic antioxidant capacity [53]. Herbal bioactives—such as flavonoids, phenolics, and essential oils—are known to modulate gut microbiota, reduce oxidative damage to enterocytes, and improve intestinal morphology, thereby supporting better nutrient digestion and absorption, including amino acids required for plasma protein synthesis [53].~~Such an increase could be linked to improved utilization of dietary protein due to a better gut health and antioxidant status.~~ ~~It has been reported that~~For example, ginger supplementation at a rate of 5 g/kg of diet has been reported to increase~~d~~ serum TP concentrations in ~~the serum of~~ broilers, likely due to an enhanced antioxidant status [54]. Additionally, Oleforuh-Okoleh et al. [55] ~~showed~~ demonstrated that adding garlic to broiler diets increased blood TP compared to the control group. The only CD treatment with a TP concentration similar to the SD groups was CD + Mix 2~~The only treatment in CD group with TP concentration similar to SD groups was CD + Mix 2~~. This suggests that Mix 2 may have improved protein retention or minimized catabolism despite the protein-deficient nature of the CD. A well-established relationship exists between dietary protein levels and plasma protein

concentration [56]; therefore, the reduced TP levels in other CD-fed groups might result from impaired protein synthesis, increased protein turnover, or hepatic dysfunction commonly associated with fatty liver development [57]~~A positive correlation between dietary protein and plasma protein concentration has been established [50]. It is possible that low protein levels in CD groups may be caused by a decrease in protein utilization, as well as an increase in protein turnover~~. Supporting this, lemon essential oil has previously been shown to increase blood TP in quail, possibly through its antioxidant properties and positive impact on liver and gut function [~~Alagawany 2021~~44]. However, not all herbal additives exert the same effect. For example, supplementation with chicory, turmeric, artichoke, and milk thistle did not significantly alter TP levels in laying hens on a high-energy, low-protein diet [~~Assi Husain~~41], and sumac inclusion at 0.25–0.5% showed no effect on TP concentration in another study [~~Mosayyeb Zadeh~~51]. ~~Studies have shown that blood TP concentration increased with the addition of lemon essential oil in quail's diets and that chicory, turmeric, artichoke, and thistle powder did not affect blood TP concentration in laying hens with fatty liver [36, 39]. Additionally, Mosayyeb Zadeh et al. [46] found that using 0, 0.25, and 0.5% sumac in the diet of laying hens had no effect on TP concentration.~~ These findings differ from the present study. Notably, the enhanced effect observed with Mix 2 may be due to synergistic interactions among its components—lemon, black pepper, sumac, and chicory. Synergism among herbs occurs when combinations of bioactive compounds interact in ways that amplify their individual physiological effects, such as through complementary antioxidant actions or cooperative effects on gut and liver function [~~Kiyohara~~58]. This could explain why Mix 2 was more effective in maintaining TP levels than individual herbs used alone in other studies.

~~The potential synergistic effects among herbs in Mix 2 may have resulted in a more pronounced outcome compared to the use of single herbs. Research has shown that the synergistic effect is based on physicochemical interactions among the bioactive compounds in herbs [51].~~

The i~~I~~ncreased blood UA concentration observed in the CD groups was not ~~compensated~~ mitigated by the addition of herbal ~~additives~~supplements. The lowest UA concentrations ~~of UA~~ were~~as~~ observed in the SD + Mix 2 and SD + Mix 3 groups. UA ~~is~~ a byproduct of protein catabolism, ~~which~~ is primarily eliminated from the body through urinary excretion. Previous r~~R~~esearch suggests that fatty liver may ~~increases~~ elevate circulatory UA levels ~~of UA~~, and UA ~~may be considere~~has been proposed~~d~~ as an independent predictor of ~~the risk for~~ fatty liver risk [~~Oral~~59]. In agreement with our results, ~~a~~ one study ~~found~~ reported that supplementing a high-energy, low-protein diet with~~dietary inclusion of~~ chicory, turmeric, artichoke, and thistle powder ~~to a high-energy, low-protein diet~~ did not significantly ~~change~~ affect UA concentrations in laying hens [~~Assi Husain~~41]. Similarly, Mosayyeb Zadeh et al. [51] ~~indicated~~ found that dietary ~~levels~~ inclusion of sumac 0.25, and 0.5% ~~sumac did not influence the concentration of~~had no significant effect on UA levels in laying hens.

In the current study, blood TG concentrations significantly increased in hens fed the CD by the second sampling point, reflecting disrupted lipid metabolism and hepatic lipid overload. Excess dietary energy and insufficient protein may impair hepatic lipid handling, causing triglycerides to accumulate in the liver and leak into circulation. ~~Blood TG concentration of laying hens was increased in CD groups in the second samplings.~~ However, in the third sampling, the CD + Mix 2 treatment attenuated this increase compared to other CD treatments. This suggests that Mix 2 may enhance lipid metabolism, reduce hepatic fat accumulation, or improve lipid clearance from the bloodstream. The lipid-lowering effect is likely mediated through the antioxidant and fat-

modulatory properties of its herbal components. Excess fat could be accumulated as TG in the liver and body tissues and streamed in the blood due to inability of the liver to deal with the overloaded of lipids. Studies suggest that sumac and its polyphenols may lower reduce TG levels by hindering fat digestion and absorption [Mosayyeb Zadeh51, Shishikura60]. Similarly, an herbal mix containing lemon was reported to reduce TG levels in overweight rats [61]. This effect may be attributed to lemon's vitamin C and flavonoid content, which act as antioxidantsThe mechanism might involve lemon's vitamin C and flavonoid content acting as antioxidants [González-Molina49, 62]. The concentration of TC concentrations was also increased under the influence of the CD in both the second and third samplings, and the herbal additives were unable to restore these this effects. Elevated TC is commonly associated with FLHS, as impaired lipid metabolism and liver dysfunction can lead to increased cholesterol synthesis and impaired excretionIt was suggested that TC and TG contents were increased in FLHS [6, 48]. Inability of herbal additives in reducing blood TC of birds in CD groups in the current study remained unexplained.

The inability of herbal mixtures to reduce TC under CD conditions may be due to insufficient duration of treatment, suboptimal dosing, or complex interactions among the bioactive compounds that diminished their cholesterol-lowering efficacy. Factors that are thought to be involved in the occurrence of FLHS include duration of feeding the challenge diet, the composition of herbal mixes, and interactions among herbal additive. The lowest TC concentration of TC was observed in the SD + Mix 3 group. which includes garlic and shallot—members of the *Allium* genus well-documented for their hypocholesterolemic effects. These effects are often attributed to sulfur-containing compounds that influence lipid metabolism and bile acid synthesis [16, 63, 64]. Garlic and shallot belong to the *Allium* specie, and several researchers have suggested that they had hypocholesterolemic effects in laying hens and quails [16, 56, 5Akbarian et al. [65] showed a

decrease in blood TC ~~of~~ in laying hens ~~treated with~~fed ginger root powder (0.25%, 0.5%, and 0.75%) over an 8-week period~~for 8 weeks~~. One possible mechanism ~~was~~ involves the modulation of bile acids [54]. Artichoke, also found in Mix 3, may contribute by enhancing biliary excretion of cholesterol and supporting liver detoxification [66].

~~Moreover, artichoke decreased blood TC through increased biliary clearance [59].~~

In the present study, feeding the CD increased blood LDL concentrations ~~of~~ in laying hens compared to the SD ~~in~~ during the second sampling.~~, and no other changes were seen in LDL concentration in laying hens fed by SD or CD with herbal additives~~ LDL, commonly referred to as "bad cholesterol," is a key contributor to lipid accumulation in blood vessels and a known risk factor for cardiovascular disease [67]. ~~. High blood LDL level is a major risk factor for heart disease [60].~~ Despite this initial increase, supplementation with herbal additives in both SD and CD groups did not lead to significant changes in LDL levels, suggesting limited impact of the additives on LDL metabolism within the timeframe of the experiment. Overall, no reduction in blood HDL was observed in the CD groups, regardless of herbal additive inclusion, when compared to the SD group~~In general, there was no decrease in blood HDL in CD groups with or without herbal additives compared to the SD in the present study~~. HDL is a type of lipoprotein responsible for transporting~~that helps transport~~ cholesterol from peripheral tissues to the liver for excretion~~elimination from the body~~. It is often referred to as "good cholesterol" due to its positive impact on reducing the risk of cardiovascular diseases [68]. The ratio of LDL to HDL in the bloodstream is an important indicator of lipid metabolism status~~The balance between LDL and HDL levels in the blood reflects the state of lipid metabolism in the body~~ [69]. Notably, in contrast to these findings, some studies have reported both increased LDL and decreased HDL in hens fed high-energy, low-protein diets [40].~~A study found an increase in LDL and a decrease in HDL in a~~

~~high energy, low protein group compared to the control group [35]. Their finding was not consistent with our result.~~ Although f~~F~~atty liver in laying hens ~~have a negative effect~~can negatively impact ~~on~~ HDL levels, this effect is not always observed~~but it does not always do so~~. Several factors—including the severity and duration of fatty liver, dietary composition, nutritional status, and genetic predisposition—can influence HDL levels.~~There are a number of factors that can influence the effect of fatty liver on HDL levels, including severity and duration of fatty liver, diet and nutrition, and genetic factors.~~ In the present study, ~~layers fed~~hens in the SD + Mix 3 group ~~showed~~ exhibited the highest ~~level of~~ HDL concentrations during both ~~in~~ the second and third samplings compared to the other treatments. Mix 3 includes garlic, ginger, artichoke, and shallot—herbs that are rich in bioactive compounds like allicin, gingerols, and cynarin, which have been associated with improved lipid profiles through mechanisms such as enhanced bile secretion, reduced hepatic lipogenesis, and increased antioxidant capacity [16, 53]. Previous research~~Studies~~ has~~ve~~ shown that ~~increased~~ HDL cholesterol levels increased in laying quails ~~when~~ fed diets containing 1%, 2%, and 4% garlic powder [70]. Similarly, Saki et al. [71] ~~showed~~ reported ~~that~~ significantly higher serum HDL levels in laying hens ~~were significantly increased when~~ fed diets ~~containing~~ supplemented with 8 or 12 g/kg~~-1~~ of an herb mixture. Moreover, an increase in serum HDL cholesterol was reported in all treated groups with g~~G~~inger rhizome oil in Japanese quails, ~~compared~~ relative to the control group [72].

The concentration of Hb ~~in~~ across different sampling times did not follow a ~~specific~~ consistent trend.~~,~~ However, overall, ~~but in general, there was an elevation of~~ Hb levels were elevated in laying hens fed the CD, both with and without herbal additives, compared to ~~the~~ those in the SD + Mix 2 and SD + Mix 3 groups. A previous study showed that a high-fat diet increased ~~the amount of~~ Hb concentrations in ~~the blood of~~ mice compared to the control diet supplemented with herbal

additives [61]. It is important to keep in mindnote that the impact relationship ofbetween fatty liver on Hb concentration in laying hens can be quiteis complex and may be depend influenced on by various factors, including the severity and etiology of underlying cause of the fatty liver, as well as the metabolic pathways involved in erythropoietin production and red blood cell destructionturnover. Another study reported elevated circulating Hb levels in patients with fatty liver, with the increase correlating with the degree of hepatic steatosis. In such cases, Hb may function as an antioxidant, mitigating some of the negative effects associated with the disease Another study indicated that patients with fatty liver had high circulating Hb level, which was related to the degree of hepatic steatosis, and this Hb may act as an antioxidant to neutralize the adverse effects of this disease [73].

In this the present study, no significant differences were observed in heterophile counts among hens fed the CD with or without herbal additives.the difference between the numbers of heterophiles in laying hens fed CD with and without additives was not significant, However, the SD + Mix 1 group exhibited the highest heterophile counts during the second and third samplings. Lymphocyte numbers but the SD + Mix 1 showed the highest count of heterophiles in the second and third sampling. The number of lymphocytes were increased in the CD groups, and while the lowest number counts was were observed in diets the SD + Mix 2 and SD + Mix 3 groups. According to a previous study, a low count of white blood cell count s may indicate suggest either a disease-free condition or suppressed production in bone marrow activity [74]. It is presumed that the medicinal plants included in these two mixesMix 2 and Mix 3 have possess anti-inflammatory, antimicrobial, and immune system-stimulating properties. The potential alleviation of fatty liver symptoms may have contributed to the observed reduction in lymphocyte numbers.It is presumed that alleviating the fatty liver symptoms could have reduced the number of lymphocytes. In support

of this, one study found that dietary supplementation with ~~By adding~~ 25 and 50 mg/kg garlic in ~~to the diet of~~ broilers had~~, a study~~ ~~found~~ no significant effect on lymphocyte counts,~~.~~ though the higher dose (50 mg/kg) significantly reduced heterophile counts compared to the control [75].~~However, the higher inclusion level (50mg/kg) significantly reduced heterophiles relative to the control [68].~~ In contrast to our results, another study reported that garlic increased heterophile~~s~~, lymphocyte~~s~~, and the heterophile to lymphocyte ratio, and attributed these responses to a stimulatory effect of garlic on the immune system [76]. In our study, the CD alone ~~resulted in more~~led to a higher lymphocyte count~~s~~ ~~compared to~~than CD + Mix 2,~~.~~ suggesting that~~It shows that~~ the combination of herbal plants in ~~this herbal mix~~Mix 2 may have attenuated ~~CD stress~~the stress associated with the CD. It is also important to consider that ~~It is worth noting that other~~ factors~~,~~ such as age, breed, and environmental conditions~~, could~~may ~~also affect~~influence heterophile levels,~~.~~ making it difficult to predict the effects of fatty liver on heterophile counts in laying hens.~~Therefore, it is difficult to predict how fatty liver will affect heterophile levels in hens.~~ Supporting this complexity, one study showed that feeding a diet containing lemon and garlic resulted in a reduced white blood cell count compared to a high-fat control diet~~A study reported that feeding a diet containing lemon and garlic decreased the number of white blood cells compared to the control diet with high fat~~ [61].

Lower liver histopathology scores were observed in hens fed the CD supplemented with Mix 2 compared to those without additives, ~~were observed in this study,~~ suggesting a potential protective role ~~for~~ of the herbs in Mix 2 against FLHS development. Although t~~T~~he precise mechanisms ~~by which Mix 2 exerts~~underlying this protective effect remain unclear,~~.~~ ~~However,~~ the individual components ~~within~~ of the herbal blend may contribute to FLHS prevention through their known biological activities~~possess properties potentially contributing to FLHS prevention~~. For example,

lemon and black pepper~~,~~ ~~known~~ are recognized for their antioxidant and anti-inflammatory effects [12, 13, 77], ~~could potentially combat~~which may help mitigate oxidative stress and inflammation— ~~in the liver,~~ key factors in FLHS pathogenesis. Sumac, ~~shown~~ reported to regulate lipid metabolism [11], could ~~potentially aid~~play a role in preventing excessive fat accumulation in the liver. ~~Moreover~~Additionally, dietary chicory has been associated with improved overall health and reduced liver histological changes [78], possibly due to its content of isoflavones, polyphenols, and antioxidants that may help reduce liver injury. ~~studies suggest that chicory in the diet may improve health and reduce liver histology changes [71]. This reduction might be due to isoflavones, polyphenols, and antioxidants present in chicory, which may mitigate liver injury [71]. The~~However, existing data on the impact of medicinal plants on liver pathology ~~is~~ are mixed. While some studies~~research~~ ~~reported~~ have found no significant effect of herbal essential oil mixtures on liver parameters [41, 79], others have demonstrated the ~~effectiveness~~ efficacy of specific herbal mixtures in reducing liver fat accumulation and inflammation in laying hens [52]. These discrepancies underscore the variability in biological responses to different herbal treatments~~These findings highlight the variability in responses~~. Trott et al. [4] noted varying ~~amounts~~ degrees of hepatocellular vacuolization in FLHS-affected -chickens, indicating that not all birds ~~have~~ experience severe hepatic fat buildup. ~~Additionally~~Similarly, Song et al. [33] reported that the severity of liver lesions ~~can~~ may vary depending on factors such as the hens' age and dietary composition. These findings highlight the complexity of FLHS and the need for further research to better understand the individual and synergistic effects of these herbs on liver health and the progression of FLHS.

~~Further research is necessary to explore the individual and synergistic effects of these herbs on liver health and FLHS development.~~

Conclusion

The present experiment ~~showed~~ demonstrated that hens fed the CD ~~had lower~~exhibited reduced FI, EW, and EP rate, ~~as well as~~along with ~~higher~~ elevated blood levels of AST (indicating liver damage), and UA (indicating protein catabolism). Additionally, t~~T~~heir livers ~~also~~ showed a higher histopathology scores consistent with FLHS. Among the herbal mixtures tested, Mix 2—comprising sumac, lemon, black pepper, and chicory—was the most effective.~~Mix 2, containing sumac, lemon, black pepper, and chicory,~~ It improved performance parameters (FI, EW, and EP rate), ~~and~~ lowered AST ~~content~~ levels, and ~~in hens fed the CD. Mix 2 also~~ reduced liver histopathology scores ~~compared to hens fed the CD alone~~in hens fed the CD. In contrast, the effects of other herbal mixtures were less consistent; some improved select parameters, while others showed no significant impact.~~The effects of other medicinal plant mixtures were less consistent. Some improved certain parameters but not others, while others had no significant effects.~~ Overall, ~~this study~~the findings suggest~~s~~ that ~~some~~ certain medicinal plant mixtures, particularly Mix 2, have the potential to ~~mitigate~~ alleviate the ~~negative~~ adverse effects of FLHS in laying hens. However, we have acknowledged key limitations of the study, including the relatively short duration, the use of a single dose per herbal mixture, the lack of individual herb evaluation, and the restriction to one hen strain and age group, which may limit the broader applicability of the findings.

Declarations

Ethics approval and consent to participate

All experimental protocols adhered to the guidelines, which were approved by, the Animal Ethics Committee of the Razi University, Kermanshah, Iran, and were in accordance with the EU standards for the protection of animals and/or feed legislation.

Consent for publication

Not applicable.

Availability of data and materials

All data generated or analysed during this study are included in this published article [and its supplementary information files].

Competing interests

The authors declare that they have no competing interests.

Funding

No funding was received to assist with the preparation of this manuscript.

Authors' contributions

Mohammadreza Khodaei conducted the experiment, gathered the data, created the result tables, and authored the initial version of the manuscript. Mehran Torki was responsible for the research design and oversight, as well as for revising and submitting the draft manuscript. Fariborz Khajali conducted the statistical analysis of the experimental data and revised the manuscript. Iraj Karimi

conducted a histopathological examination of the liver. All authors reviewed and endorsed the final manuscript.

Acknowledgements

The authors appreciate Razi University for financially supporting the current study.

References

1. Aydin, R. 2005. Type of fatty acids, lipoprotein secretion from liver and fatty liver syndrome in laying hens. Int J Poult Sci. 2005;4:917-919.

2. Butler EJ. Lipid metabolism in the fowl under normal and abnormal circumstances. Proc Nutr Soc. 1975;34:29-34. https://doi.org/10.1079/PNS19750007

3. Butler EJ. Fatty liver diseases in the domestic fowl—A review. Avian Pathol. 1976;5:1-4. https://doi.org/10.1080/03079457608418164

4. Trott KA, Giannitti F, Rimoldi G, Hill A, Woods L, Barr B, et al. Fatty liver hemorrhagic syndrome in the backyard chicken: a retrospective histopathologic case series. Vet Pathol. 2014;51:787-95. https://doi.org/10.1177/03009858135035

5. Shini A, Shini S, Bryden WL. Fatty liver haemorrhagic syndrome occurrence in laying hens: impact of production system. Avian Pathol. 2019;48:25-34. https://doi.org/10.1080/03079457.2018.1538550

6. Choi YI, Ahn HJ, Lee BK, Oh ST, An BK, Kang CW. Nutritional and hormonal induction of fatty liver syndrome and effects of dietary lipotropic factors in egg-type male chicks. Asian-Australas J Anim Sci. 2012;25:1145. https://doi.org/10.5713/ajas.2011.11418

7. Jiang S, Cheng HW, Cui LY, Zhou ZL, Hou JF. Changes of blood parameters associated with bone remodeling following experimentally induced fatty liver disorder in laying hens. Poult Sci. 2013;92:1443-53. https://doi.org/10.3382/ps.2012-02800

8. Squires EJ, Leeson S. Aetiology of fatty liver syndrome in laying hens. Br Vet J. 1988;144:602-609. https://doi.org/10.1016/0007-1935(88)90031-0

9. Pearson AW, Butler EJ, Curtis RF, Fenwick GR, Hobson-Frohock A, Land DG, et al. Effects of rapeseed meal on laying hens (*Gallus domesticus*) in relation to fatty liver-haemorrhagic syndrome and egg taint. Res Vet Sci. 1978;25:307-13. https://doi.org/10.1016/S0034-5288(18)32945-X

10. Couch JR. Fatty livers in laying hens—a condition which may occur as a result of increased strain. Feedstuffs. 1956;28(47):46.

11. Shariatmadari F, Shariatmadari R. Sumac (*Rhus coriaria*) supplementation in poultry diet. World's Poult Sci. J 2020;76:358-64. https://doi.org/10.1080/00439339.2020.1745724

12. Manners GD. Citrus limonoids: analysis, bioactivity, and biomedical prospects. J Agric Food Chem. 2007;55:8285-94. https://doi.org/10.1021/jf071797h

13. Zuzanna Bober Z, Agnieszka Stępień A, David Aebisher D, Łukasz Ożog Ł, Dorota Bartusik-Aebisher D. Medicinal benefits from the use of Black pepper, Curcuma and Ginger. Eur J Clin Exp Med. 2018;133-45. 10.15584/ejcem.2018.2.9

14. Nandagopal S, Kumari BR. Phytochemical and antibacterial studies of Chicory (*Cichorium intybus L.*)-A multipurpose medicinal plant. Adv Biol Res. 2007;1:17-21.

15. Khazaei R, Seidavi A, Bouyeh M. A review on the mechanisms of the effect of silymarin in milk thistle (*Silybum marianum*) on some laboratory animals. Vet Med Sci. 2022;8:289-301. https://doi.org/10.1002/vms3.641

16. Kothari D, Lee WD, Niu KM, Kim SK. The genus Allium as poultry feed additive: A review. Animals. 2019;9:1032. https://doi.org/10.3390/ani9121032

17. Abadjieva D, Grigorova S, Mladenova V, Shimkus A, Kistanova E. Effect of artichoke (*Cynara scolimus L.*) on the egg productivity and biochemical parameters in laying hens. Bulg J Agric Sci. 2020; 26: 1280-5.

18. Chung MY, Park HJ, Manautou JE, Koo SI, Bruno RS. Green tea extract protects against nonalcoholic steatohepatitis in ob/ob mice by decreasing oxidative and nitrative stress responses induced by proinflammatory enzymes. J Nutr Biochem. 2012;23:361-7. https://doi.org/10.1016/j.jnutbio.2011.01.001

19. Kermanshahi H, Riasi A. Effect of turmeric rhizome powder (*Curcuma longa*) and soluble NSP degrading enzyme on some blood parameters of laying hens. Int J Poult Sci. 2006;5:494-8.

20. Sharma UR, Prakash T, Surendra V, Roopakarki N, Rao NR, Divakar G. Hepatoprotective activity of Fumaria officinalis against CCl4-induced liver damage in rats. Pharmacology. 2012;3:9-14.

21. Khan RU, Naz S, Nikousefat Z, Tufarelli VI, Javdani M, Qureshi MS, et al. Potential applications of ginger (*Zingiber officinale*) in poultry diets. World's Poult Sci J. 2012;68:245-52. https://doi.org/10.1017/S004393391200030X

22. Li XL, He WL, Wang ZB, Xu TS. Effects of Chinese herbal mixture on performance, egg quality and blood biochemical parameters of laying hens. J Anim Physiol Anim Nutr. 2016;100:1041-9. https://doi.org/10.1111/jpn.12473

23. Lohmann Breeders. 2020. LSL-Lite Layers-Management Guide. Lohmann, Cuxhaven, Germany. https://lohmann-breeders.com/media/strains/cage/management/LOHMANN-LSL-Lite-Cage-1.pdf

24. Marai IF, Habeeb AA, Gad AE. Rabbits' productive, reproductive and physiological performance traits as affected by heat stress: a review. Livest Prod Sci. 2002;78(2):71-90. https://doi.org/10.1016/S0301-6226(02)00091-X

25. Naghneh NS, Rafiei F, Shahbazi E, Gheisari MM. Variation of volatile compounds in celery through different vegetative stages. Plant Physiol. 2023;28:476-80. https://doi.org/10.1007/s40502-023-00744-6

26. Anene DO, Akter Y, Thomson PC, Groves P, O'Shea CJ. Variation and association of hen performance and egg quality traits in individual early-laying ISA Brown Hens. Animals. 2020;10(9):1601. https://doi.org/10.3390/ani10091601

27. Kianfar R, Di Rosa AR, Divari N, Janmohammadi H, Hosseintabar-Ghasemabad B, Oteri M, et al. A comparison of the effects of raw and processed amaranth grain on laying hens' performance, egg physicochemical properties, blood biochemistry and egg fatty acids. Animals. 2023;13(8):1394.

28. Cherian G, Hayat Z. Long-term effects of feeding flaxseeds on hepatic lipid characteristics and histopathology of laying hens. Poult Sci. 2009;88:2555-61. https://doi.org/10.3382/ps.2009-00425

29. SAS (Statistical Analyses System). 2015. SAS user's guide. Version 9.4. Cary, NC: SAS Inst.

30. Karmakar R, Pahari P, Mal D. Phthalides and Phthalans: Synthetic methodologies and their applications in the total synthesis. Chem Rev. 2014;114: 6213–84. https://doi.org/10.1021/cr400524q

31. Moses T, Pollier J, Thevelein J M, Goossens A. Bioengineering of plant (tri) terpenoids: From metabolic engineering of plants to synthetic biology in vivo and in vitro. New Phytologist. 2013;200: 27–43. https://doi.org/10.1111/nph.12325

32. Rozenboim I, Mahato J, Cohen NA, Tirosh O. Low protein and high-energy diet: a possible natural cause of fatty liver hemorrhagic syndrome in caged White Leghorn laying hens. Poult Sci. 2016;95:612-21. https://doi.org/10.3382/ps/pev367

33. Song Y, Ruan J, Luo J, Wang T, Yang F, Cao H, et al. Abnormal histopathology, fat percent and hepatic apolipoprotein AI and apolipoprotein B100 mRNA expression in fatty liver

hemorrhagic syndrome and their improvement by soybean lecithin. Poult Sci. 2017;96:3559-63. https://doi.org/10.3382/ps/pex163

34. McNab JM, Boorman KN. Poultry feedstuffs: supply, composition and nutritive value. CABI publishing. 2002. https://doi.org/10.1079/9780851994642.0000

35. Richards MP. Genetic regulation of feed intake and energy balance in poultry. Poult Sci. 2003;82:907-16. https://doi.org/10.1093/ps/82.6.907

36. Zaefarian F, Abdollahi MR, Cowieson A, Ravindran V. Avian liver: the forgotten organ. Animals. 2019;9(2):63. https://doi.org/10.3390/ani9020063

37. Shini S, Bryden WL. Occurrence and control of fatty liver haemorrhagic syndrome (FLHS) in caged hens. A report for the Australian Egg Corporation Limited AECL Publication No uq-105a AECL Project No UQ-105. 2009;1448-316.

38. Lee BK, Kim JS, Ahn HJ, Hwang JH, Kim JM, Lee HT, et al. Changes in hepatic lipid parameters and hepatic messenger ribonucleic acid expression following estradiol administration in laying hens (*Gallus domesticus*). Poult Sci. 2010;89:2660-7. https://doi.org/10.3382/ps.2010-00686

39. Panyod S, Sheen LY. Beneficial effects of Chinese herbs in the treatment of fatty liver diseases. J Tradit Complement Med. 2020;10:260-7. https://doi.org/10.1016/j.jtcme.2020.02.008

40. Miao YF, Gao XN, Xu DN, Li MC, Gao ZS, Tang ZH, et al. Protective effect of the new prepared Atractylodes macrocephala Koidz polysaccharide on fatty liver hemorrhagic syndrome in laying hens. Poult Sci. 2021;100:938-48. https://doi.org/10.1016/j.psj.2020.11.036

41. Assi Husain H, Sadeghi A, Karimi A. Effects of chicory, turmeric, artichoke and sage powder in high energy and low protein diets on yield, egg quality and fatty liver status in laying hens. Anim Sci. 2023;36:19-38. 10.22092/ASJ.2022.358220.2215

42. Izadi H, Arshami J, Golian A, Raji MR. Effects of chicory root powder on growth performance and histomorphometry of jejunum in broiler chicks. Vet Res Forum. 2013;4:169-174.

43. Arpášová H, Kačániová M, Gálik B, Čuboň J, Mellen M. The effect of oregano essential oil and Rhus coriaria L. on selected performance parameters of laying hens. Scientific Papers Anim Sci Biotech. 2014;47:12-12.

44. Alagawany M, El-Saadony MT, Elnesr SS, Farahat M, Attia G, Madkour M, et al. Use of lemongrass essential oil as a feed additive in quail's nutrition: its effect on growth, carcass, blood biochemistry, antioxidant and immunological indices, digestive enzymes and intestinal microbiota. Poult Sci. 2021;100:101172. https://doi.org/10.1016/j.psj.2021.101172

45. Omer HA, Ahmed SM, Abdel-Magid SS, El-Mallah GM, Bakr AA, Abdel Fattah MM. Nutritional impact of inclusion of garlic (Allium sativum) and/or onion (Allium cepa L.) powder in laying hens’ diets on their performance, egg quality, and some blood constituents. Bull Natl Res Cent. 2019;43:1-9. https://doi.org/10.1186/s42269-019-0061-6

46. Park SO, Park BS. Effect of feeding inulin oligosaccharides on cecum bacteria, egg quality and egg production in laying hens. Afric J Biotech. 2012;11:9516-21. https://doi.org/10.5897/AJB12.5250

47. Yousefi M, Shivazad M, Sohrabi-Haghdoost I. Effect of dietary factors on induction of fatty liver-hemorrhagic syndrome and its diagnosis methods with use of serum and liver parameters in laying hens. Int J Poult Sci. 2005;4:468-72.

48. Zhang J, Chen D, Yu B. Effect of different dietary energy sources on induction of fatty liver-hemorrhagic syndrome in laying hens. Int J Poult Sci. 2008;7:1232-6.

49. González-Molina E, Domínguez-Perles R, Moreno DA, García-Viguera C. Natural bioactive compounds of Citrus limon for food and health. J Pharm Biomed Anal. 2010;51:327-45. https://doi.org/10.1016/j.jpba.2009.07.027

50. Gumus H, Oguz MN, Bugdayci KE, Oguz FK. Effects of sumac and turmeric as feed additives on performance, egg quality traits, and blood parameters of laying hens. Rev Bras de Zootecn. 2018;47. https://doi.org/10.1590/rbz4720170114

51. Mosayyeb Zadeh A, Mirghelenj SA, Shakouri Alishah H, Hasanlou P. Effects of sumac fruit powder (Rhus coriaria L.) supplementation on productive performance, egg quality traits and serum biochemical parameters in old laying hens. Ital J Anim Sci. 2021;20:1348-56. https://doi.org/10.1080/1828051X.2021.1965922

52. Zhu Y, Zhang X, Du P, Wang Z, Luo P, Huang Y, et al. Dietary herbaceous mixture supplementation reduced hepatic lipid deposition and improved hepatic health status in post-peak laying hens. Poult Sci. 2022;101:101870. https://doi.org/10.1016/j.psj.2022.101870

53. Abdelli N, Solà-Oriol D, Pérez JF. Phytogenic feed additives in poultry: achievements, prospective and challenges. Animals. 2021;11(12):3471. https://doi.org/10.3390/ani11123471

54. Zhang GF, Yang ZB, Wang Y, Yang WR, Jiang SZ, Gai GS. Effects of ginger root (Zingiber officinale) processed to different particle sizes on growth performance, antioxidant status, and serum metabolites of broiler chickens. Poult Sci. 2009;88:2159-66. https://doi.org/10.3382/ps.2009-00165

55. Oleforuh-Okoleh VU, Ndofor-Foleng HM, Olorunleke SO, Uguru JO. Evaluation of growth performance, haematological and serum biochemical response of broiler chickens to aqueous extract of ginger and garlic. J Agric Sci. 2015;7:167. https://doi.org/10.5539/jas.v7n4p167

56. Bush BM. Interpretation of laboratory results for small animal clinicians. Blackwell Scientific Publications Ltd. 1991;225-9.

57. Du X, Wang Y, Amevor FK, Ning Z, Deng X, Wu Y, et al. Effect of high energy low protein diet on lipid metabolism and inflammation in the liver and abdominal adipose tissue of laying hens. Animals. 2024;14(8):1199. https://doi.org/10.3390/ani14081199

58. Kiyohara H, Matsumoto T, Yamada H. Combination effects of herbs in a multi-herbal formula: expression of Juzen-taiho-to's immuno-modulatory activity on the intestinal immune system. J Evid Based Complementary Altern Med. 2004;1:83-91. https://doi.org/10.1093/ecam/neh004

59. Oral A, Sahin T, Turker F, Kocak E. Relationship between serum uric acid levels and nonalcoholic fatty liver disease in non-obese patients. Med. 2019;55:600. https://doi.org/10.3390/medicina55090600

60. Shishikura Y, Khokhar S, Murray BS. Effects of tea polyphenols on emulsification of olive oil in a small intestine model system. J Agric Food Chem. 2006;54:1906-13. https://doi.org/10.1021/jf051988p

61. Bassuony NI, Hafez LM, Ibrahim AF, El-Sebeay AS, Omer HA. Effect of using natural bioactive mixture composed of lemon, onion and garlic juices on weight and lipid profiles for overweight rats. Eur J Appl Sci. 2021;13:09-17.

62. Prabuseenivasan S, Jayakumar M, Ignacimuthu S. In vitro antibacterial activity of some plant essential oils. BMC Complement Altern Med. 2006;6:1-8. https://doi.org/10.1186/1472-6882-6-39

63. Chowdhury SR, Chowdhury SD, Smith TK. Effects of dietary garlic on cholesterol metabolism in laying hens. Poult Sci. 2002;81:1856-62. https://doi.org/10.1093/ps/81.12.1856

64. Mahmoud KZ, Gharaibeh SM, Zakaria HA, Qatramiz AM. Garlic (*Allium sativum*) supplementation: Influence on egg production, quality, and yolk cholesterol level in layer hens. Asian-Australas J Anim Sci. 2010;23:1503-9. https://doi.org/10.5713/ajas.2010.10124

65. Akbarian A, Golian A, Sheikh Ahmadi A, Moravej H. Effects of ginger root (*Zingiber officinale*) on egg yolk cholesterol, antioxidant status and performance of laying hens. J Appl Anim Res. 2011;39:19-21. https://doi.org/10.1080/09712119.2011.558612

66. Qiang Z, Lee SO, Ye Z, Wu X, Hendrich S. Artichoke extract lowered plasma cholesterol and increased fecal bile acids in Golden Syrian hamsters. Phytother Res. 2012;26:1048-52. https://doi.org/10.1002/ptr.3698

67. Ridker PM. LDL cholesterol: controversies and future therapeutic directions. Lancet. 2014;384:607-17. https://doi.org/10.1016/S0140-6736(14)61009-6

68. Hermansen K, Dinesen B, Hoie LH, Morgenstern E, Gruenwald J. Effects of soy and other natural products on LDL: HDL ratio and other lipid parameters: a literature review. Adv Ther. 2003;20:50-78. https://doi.org/10.1007/BF02850119

69. Zhang X, Han Z, Zhong H, Yin Q, Xiao J, Wang F, et al. Regulation of triglyceride synthesis by estradiol in the livers of hybrid tilapia (Oreochromis niloticus♀× O. aureus♂). Comparative Biochemistry and Physiology Part B: Biochem Mol Biol. 2019;238:110335. https://doi.org/10.1016/j.cbpb.2019.110335

70. Canogullari S, Baylan M, Erdogan Z, Duzguner V, Kucukgul A. The effects of dietary garlic powder on performance, egg yolk and serum cholesterol concentrations in laying quails. Czech J Anim Sci. 2010;55:286-93. https://doi.org/10.17221/126/2009-CJAS

71. Saki AA, Aliarabi H, Siyar SA, Salari J, Hashemi M. Effect of a phytogenic feed additive on performance, ovarian morphology, serum lipid parameters and egg sensory quality in laying hen. Vet Res Forum. 2014;5:287–293.

72. Herve T, Raphaël KJ, Ferdinand N, Victor Herman N, Willy Marvel NM, Cyril D'Alex T, et al. Effects of ginger (*Zingiber officinale*, *Roscoe*) essential oil on growth and laying performances, serum metabolites, and egg yolk antioxidant and cholesterol status in laying Japanese quail. J Vet Med. 2019;2019. https://doi.org/10.1155/2019/7857504

73. Juárez-Hernández E, Chávez-Tapia NC, Brizuela-Alcántara DC, Uribe M, Ramos-Ostos MH, Nuño-Lámbarri N. Association between serum hemoglobin levels and non alcoholic fatty liver disease in a mexican population. Ann Hepatol. 2018;17:577-84. https://doi.org/10.5604/01.3001.0012.0920

74. Medugu CI, Kwari ID, Igwebuike J, Nkama I, Mohammed ID, Hamaker B. Carcass and blood components of broiler chickens fed sorghum or millet as replacement for maize in the semi arid zone of Nigeria. Agric Biol J North Am. 2010;1:326-9.

75. El-katcha MI, Soltan MA, Sharaf MM, Hasen A. Growth performance, immune response, blood serum parameters, nutrient digestibility and carcass traits of broiler chicken as affected by dietary supplementation of garlic extract (allicin). Alex J Vet Sci. 2016 15;49. https://doi.org/10.5455/ajvs.219261

76. Eid KM, Iraqi MM. Effect of garlic powder on growth performance and immune response for newcastle and avian influenza virus diseases in broiler of chickens. Anim Biotechnol. 2014;1:7-13.

77. Li Y, Yang M, Lin H, Yan W, Deng G, Ye H, et al. Limonin alleviates non-alcoholic fatty liver disease by reducing lipid accumulation, suppressing inflammation and oxidative stress. Front Pharmacol. 2022;12:801730.

78. Jamzadfard M, Ebrahimi H. Herbal Medicine in the Treatment of Fatty Liver Disease: Evaluation of the therapeutic Effects of some herbs that constitute Magic Liver herbal distillate.

79. Mousavi A, Mahdavi AH, Riasi A, Soltani-Ghombavani MJ. Synergetic effects of essential oils mixture improved egg quality traits, oxidative stability and liver health indices in laying hens fed fish oil. Anim Feed Sci Technol. 2017;234:162-72.

80. Amiri HR, Mohammadi M, Sadatmand S, Taheri E. Study the chemical composition of essential oil of ginger (*zingiber officinale*) and antioxidant and cell toxicity. J Med Plants. 2016 ;15:89-98.

81. Majdi B, Mehrnia MA, Barzegar H, Alizadeh Behbahani B. Determination of the structure, chemical composition, antioxidant activity and the cytotoxic effect of Turmeric essential oil. Iran Food Sci Technol Res J. 2021;17(2):261-71. https://doi.org/10.22067/ifstrj.2020.39272

82. Khoshtinat K, Barzegar M, Sahari MA, Hamidi Z. Encapsulation of Iranian Garlic Oil with β-cyclodextrin: Optimization and its Characterization. J Agric Sci Technol. 2017;19: 97-111.

83. Morshed S, Hossain MD, Ahmad M, Junayed M. Physicochemical characteristics of essential oil of black pepper (*Piper nigrum*) cultivated in Chittagong, Bangladesh. J. Food Qual Hazards Control. 2017;4(3):66-9.
