# Supplementary material for: Application of various mixtures of medicinal herbs in the diet of laying hens: Evaluating preventive approach of fatty liver syndrome
Source: PLoS One. 2025 Oct 24;20(10):e0330363. doi: 10.1371/journal.pone.0330363 (PMC12551855; doi:10.1371/journal.pone.0330363)
Supplement: Attachment — Submitted filename: Review of Article-20250523.docx [file pone.0330363.s003.docx]

**Summary:**

The author conducted this study to evaluate three herbal mixes to prevent fatty liver hemorrhagic syndrome (FLHS) in laying hens fed either a standard diet (SD) or a challenge diet (CD; high-energy, low-protein). Among 384 hens aged 65–77 weeks, those on the CD showed reduced feed intake, egg production, and egg weight, alongside increased feed conversion ratio, liver damage, and blood triglyceride and AST levels. Of the herbal mixes tested, Mix 2 (lemon, black pepper, sumac, chicory) significantly improved all negative effects induced by the CD. Mixes did not alter immune cells or hemoglobin levels. Overall, Mix 2 was the most effective intervention to mitigate CD-induced FLHS and support productive performance. However, there are still some comments that need to be addressed by the author.

**Comments:**

1. Line 37, the author could change “background” to “introduction”, because “background” may be confused as the same one in Abstract.
2. Lines 38–42: The point that the liver synthesizes lipids and is heavily burdened in hens is repeated. The author could try to combine them with a single sentence.
3. The methods mentioned in the manuscript should be cited formally. Such as Line 51-54.
4. Lines 55–64: The paragraph listing all plants and their bio-actives is dense and hard to read. The author could Use a table or bullet points for readability or briefly summarize: "These herbs are rich in flavonoids, alkaloids, sulfur compounds, and dietary fibers, with demonstrated antioxidant, anti-inflammatory, and lipid-lowering properties."
5. Lines 37-69, the author could use clearer topic sentences to guide the reader through each section logically, including liver function, disease description, economic impact, and herbal solutions.
6. Lines 88–90, the author use informal phrasing “running into the experimental trial", which can be corrected to "A two-week adaptation period was implemented to acclimate hens to the diets prior to the 12-week experimental trial."
7. For animal study design, the randomization or blinding details were missing. Please mark those in the experiment design part.
8. No mention of sample size calculation or statistical power. The author could revise as "Sample size was determined based on expected effect sizes from previous studies to achieve 80% power at α = 0.05."
