# Supplementary material for: Application of various mixtures of medicinal herbs in the diet of laying hens: Evaluating preventive approach of fatty liver syndrome
Source: PLoS One. 2025 Oct 24;20(10):e0330363. doi: 10.1371/journal.pone.0330363 (PMC12551855; doi:10.1371/journal.pone.0330363)
Supplement: Attachment — Submitted filename: Respone to Reviewers.docx [file pone.0330363.s008.docx]

**Reviewer #1**: The manuscript is well written and addresses an important question in the field. The authors have responded thoroughly to all previous comments and have made the necessary revisions to improve the clarity, rigor, and impact of the work. I have no further concerns regarding scientific content, ethics, or publication practices. I recommend the manuscript for publication in its current form.

Thank you very much, indeed.

**Reviewer #2**: Dear Authors, Follow my comments in the attached file, in the materials and methods section, give details about cage manufacturer and stocking density per bird.

Thank you. The stocking density has been already provided in the *Materials and Methods* section, as stated in the following sentence:

*“The hens were housed in wire cages (Machine Toyour, Co., Juybar, Mazandaran, Iran) measuring 45 × 45 × 45 cm, with three birds per cage, corresponding to a stocking density of 675 cm² per bird.”*
The name of cage manufacturer has now been added (Page 5, Lines 91-92).

Also remove the model used for THI by Marai et al. as this study focuses on Rabbits and this model is applicable to different species. Remove this model and add the appropriate model suitable for chickens.
Thank you for bringing this up. We have replaced the previous THI model by a new THI model specifically used for poultry (Boonkum et al., 2025).

Improve the logical reasoning of the results.

Thank you for your comment. Based on the available literature, we have improved the logical reasoning of the results by clarifying the arguments, aligning our findings with previous studies, and strengthening the supporting evidence. Please see the changes in the revised manuscript.

In conclusion, add limitations of the study

Thank you for your comment. The limitation of the study had already been acknowledged in the conclusion section (Page 31, Lines 563-566).
